# Linear-Structure Single-Atom Gold(I) Catalyst for Dehydrogenative Coupling of Organosilanes with Alcohols

**DOI:** 10.1021/acscatal.3c03937

**Published:** 2023-11-30

**Authors:** Ravishankar G. Kadam, Miroslav Medved’, Subodh Kumar, Dagmar Zaoralová, Giorgio Zoppellaro, Zdeněk Bad’ura, Tiziano Montini, Aristides Bakandritsos, Emiliano Fonda, Ondřej Tomanec, Michal Otyepka, Rajender S. Varma, Manoj B. Gawande, Paolo Fornasiero, Radek Zbořil

**Affiliations:** † Regional Centre of Advanced Technologies and Materials, Czech Advanced Technology and Research Institute, (CATRIN), Palacký University Olomouc, Šlechtitelu° 27, Olomouc 779 00, Czech Republic; ‡ Department of Chemistry, Faculty of Natural Sciences, Matej Bel University, Tajovského 40, Banská Bystrica 974 01, Slovak Republic; § Department of Inorganic Chemistry, Faculty of Science, Palacký University Olomouc, 17. listopadu 12, Olomouc 779 00, Czech Republic; ∥ IT4Innovations, VŠB−Technical University of Ostrava, 17. listopadu 2172/15, Ostrava, Poruba 708 00, Czech Republic; ⊥ CEET, Nanotechnology Centre, VŠB−Technical University of Ostrava, 17. listopadu 2172/15, Ostrava, Poruba 708 00, Czech Republic; # Department of Chemical and Pharmaceutical Sciences, Center for Energy, Environment and Transport Giacomo Ciamiciam, INSTM Trieste Research Unit and ICCOM-CNR Trieste Research Unit, University of Trieste via L. Giorgieri 1, Trieste I-34127, Italy; ∇ Synchrotron SOLEIL, L’Orme des Merisiers, Saint Aubin 91190, France; ○ Department of Industrial and Engineering, Chemistry Institute of Chemical Technology, Mumbai-Marathwada Campus, Jalna, Maharashtra 431213, India

**Keywords:** single-gold-atom catalysis, cyanographene, dehydrogenative coupling, organosilanes, alkoxysilanes, flow chemistry

## Abstract

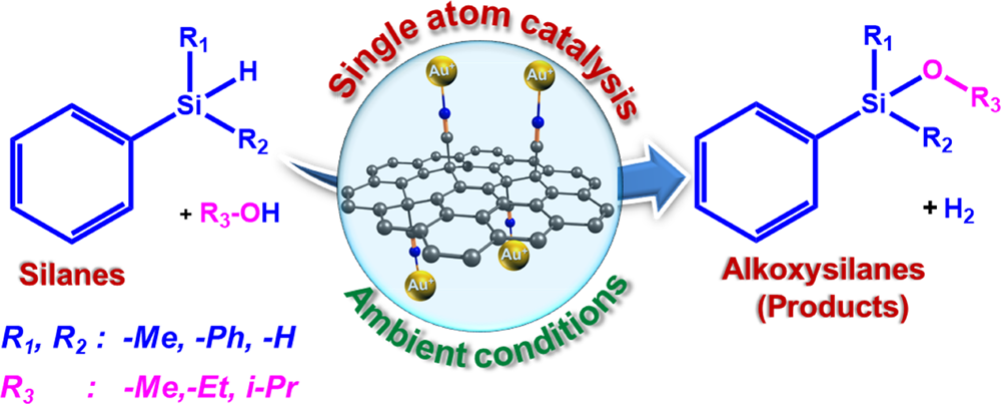

A strategy for the synthesis of a gold-based single-atom catalyst (SAC) via a one-step room temperature reduction of Au(III) salt and stabilization of Au(I) ions on nitrile-functionalized graphene (cyanographene; G-CN) is described. The graphene-supported G(CN)-Au catalyst exhibits a unique linear structure of the Au(I) active sites promoting a multistep mode of action in dehydrogenative coupling of organosilanes with alcohols under mild reaction conditions as proven by advanced XPS, XAFS, XANES, and EPR techniques along with DFT calculations. The linear structure being perfectly accessible toward the reactant molecules and the cyanographene-induced charge transfer resulting in the exclusive Au(I) valence state contribute to the superior efficiency of the emerging two-dimensional SAC. The developed G(CN)-Au SAC, despite its low metal loading (ca. 0.6 wt %), appear to be the most efficient catalyst for Si–H bond activation with a turnover frequency of up to 139,494 h^–1^ and high selectivities, significantly overcoming all reported homogeneous gold catalysts. Moreover, it can be easily prepared in a multigram batch scale, is recyclable, and works well toward more than 40 organosilanes. This work opens the door for applications of SACs with a linear structure of the active site for advanced catalytic applications.

## Introduction

Heterogeneous catalysis epitomizes performance enhancement by decreasing the size of catalytically active particles from nano to subnanoparticles.^1^ Among them, single-atom catalysts (SACs) have garnered protuberant attention due to their maximized atom utilization efficiency and cost-effective nature, while making every active site accessible to the reactants, thus affirming a great potential in catalysis owing to the combined merits of homogeneous and heterogeneous catalysts.^1,2^ To date, nitrogen-doped two-dimensional carbon-supported SACs have been dominantly explored,^3^ displaying prominent catalytic activities^4^ often represented by noble metal-containing SACs such as Pt,^5^ Pd,^5,6^ Rh,^7^ Au,^5^ and Ir^8^ among others.

**Figure 1:**
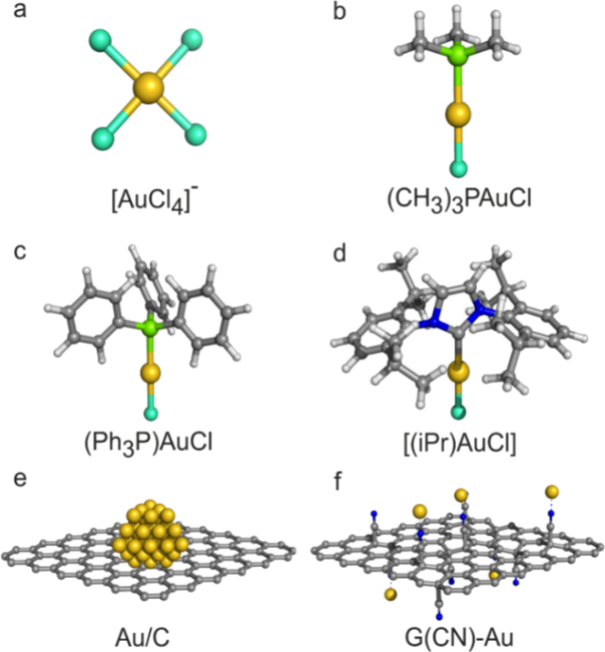
Structural comparison of gold catalysts. Schematic illustration of Au-based catalysts. (a) [AuCl_4_]^−^, (b) (CH_3_)_3_PAuCl, (c) (Ph_3_P)AuCl, (d) [(iPr)AuCl], (e) gold NPs adorned on carbon or graphene, and (f) Au(I) cations anchored on G-CN (this study). Gold atoms are yellow, chlorine is blue-green, phosphorus is green, carbon is gray, nitrogen is blue, and hydrogen is white.

Gold represents the prominent element in catalysis, which can be applied in various forms, including homogeneous Au(I) metal complexes or nanoparticles (NPs) entrapped onto various supports (Figure 1a–e). Gold-supported SACs and NPs have become an integral part of heterogeneous catalysis since the pioneering discoveries by Haruta,^9^ exhibiting size-dependent activity in numerous reactions, namely, CO oxidation,^10^ alkane oxidation,^11^ thiophenol oxidation,^12^ and ethyne hydrochlorination.^13^ Recently, Hutchings et al.^3,4^ presented the extraordinary performance of heterogeneous Au single-site catalysts (SSCs)^3,4^ at a higher oxidation state for acetylene hydrochlorination and three-component coupling reactions.^14^

Dehydrogenative oxidation of the silane via Si–H bond activation has garnered widespread attention in view of its nontoxic attributes as a reactant with hydrogen being the only byproduct, thus rendering these processes clean and environmentally benign. Importantly, the main products—alkoxysilane and silanols—are prominent commodity chemicals and high value-added reagents extensively deployed in advanced material synthesis such as elastomers,^15^ fibers,^16^ biomedical materials, coupling reagents,^17^ and other important organic (protection and deprotection of hydroxyl groups) and biological applications.^18^ Generally, alkoxysilane synthesis is conducted via a reaction of chlorosilanes^19^ with alcohols in the presence of strong bases, but the associated moisture sensitivity, corrosiveness, and the formation of stoichiometric amounts of salts as a byproduct renders these methods environmentally detrimental. To date, several efficient homogeneous metal complexes have been utilized for Si–H bond activation^20^ along with corresponding carbon-based heterogeneous catalysts using pyrolysis strategies owing to the ease in catalyst recycling^21−24^ among which Au NPs supported on various supports have exhibited excellent activity^25^ and selectivity.^26^ However, the high loading of precious metals, longer reaction times, preparation methods of complex catalysts, and smaller substrate scope are some of the lingering major limitations of these systems.

In contrast to Au NP catalysts, SACs with low-valence Au active sites have revealed superior reactivity for the Si–H bond activation.^2^

This work showed the huge potential of a gold(I) SAC as the achieved TOF values (ca. ∼139494 h^–1^) are very promising from the practical point of view and also the unique linear-structure of the single-atom gold(I) catalyst highlights the effect of single-atom (SA) arrangement on the efficiency in Si–H bond activation.

Generally, assorted reducing agents and *N*-doped carbon supports have emerged as ideal candidates to anchor the Au SAs.^27^ However, the immobilization and identification of the selective valence state of Au SA surface-active species^28^ toward their catalytic properties have remained to be a crucial challenge and constraint^4^ to date. To overcome the aforementioned problems, the major key factor, namely the availability of maximum anchoring sites on the support to immobilize the selective oxidation state of Au SAs, is highly endorsed. Further, conforming to sustainable catalysis, it is highly desirable to reduce the cost by minimizing the content of noble metals (e.g., Au, Pd, and Pt) in chemical reactions. Thus, we hypothesize that the integration of atomically dispersed Au SAs with low metal loadings but discerning active oxidation states for catalysis would make them an ideal and sought-after candidate.

Herein, we report a straightforward and clean single-step strategy for the synthesis of Au-based SACs with isolated Au(I) cations keeping the unique linear structure and being uniformly dispersed on the cyanographene (G-CN) support, which is hereafter referred to as G(CN)-Au. Cyanographene with homogeneously distributed cyano groups and ample functionalization (ca. 15%) was synthesized via the chemistry of fluorographene.^29^ The isolated single cationic Au(I) sites of G(CN)-Au keep the linear structure with nitrogen and oxygen atom contributions to linear coordination and coming from CN and water/OH^–^ ligands, respectively. The G(CN)-Au SACs are highly selective with excellent catalytic prowess for silicon–hydrogen (Si–H) bond activation^30^ (Figure 1f), reaching the highest reported TOF of up to 139,494 h^–1^ for the dehydrogenative coupling of organosilane with alcohols (TOF calculation details explained in the Supporting Information). The unique linear structure of Au SACs significantly contributes to record TOF values as proven by detailed DFT study of the reaction mechanism as the SACs are well accessible toward the reactant molecules.

An important salient feature of the approach includes a wide range of substrate compatibility, gram-scale applicability, recyclability, and adaptation for a continuous-flow mode with low Au loadings with the properties highly advantageous for advanced catalytic applications.

## Results and Discussion

### Synthesis and Characterization of the G(CN)-Au catalyst

**Figure 2:**
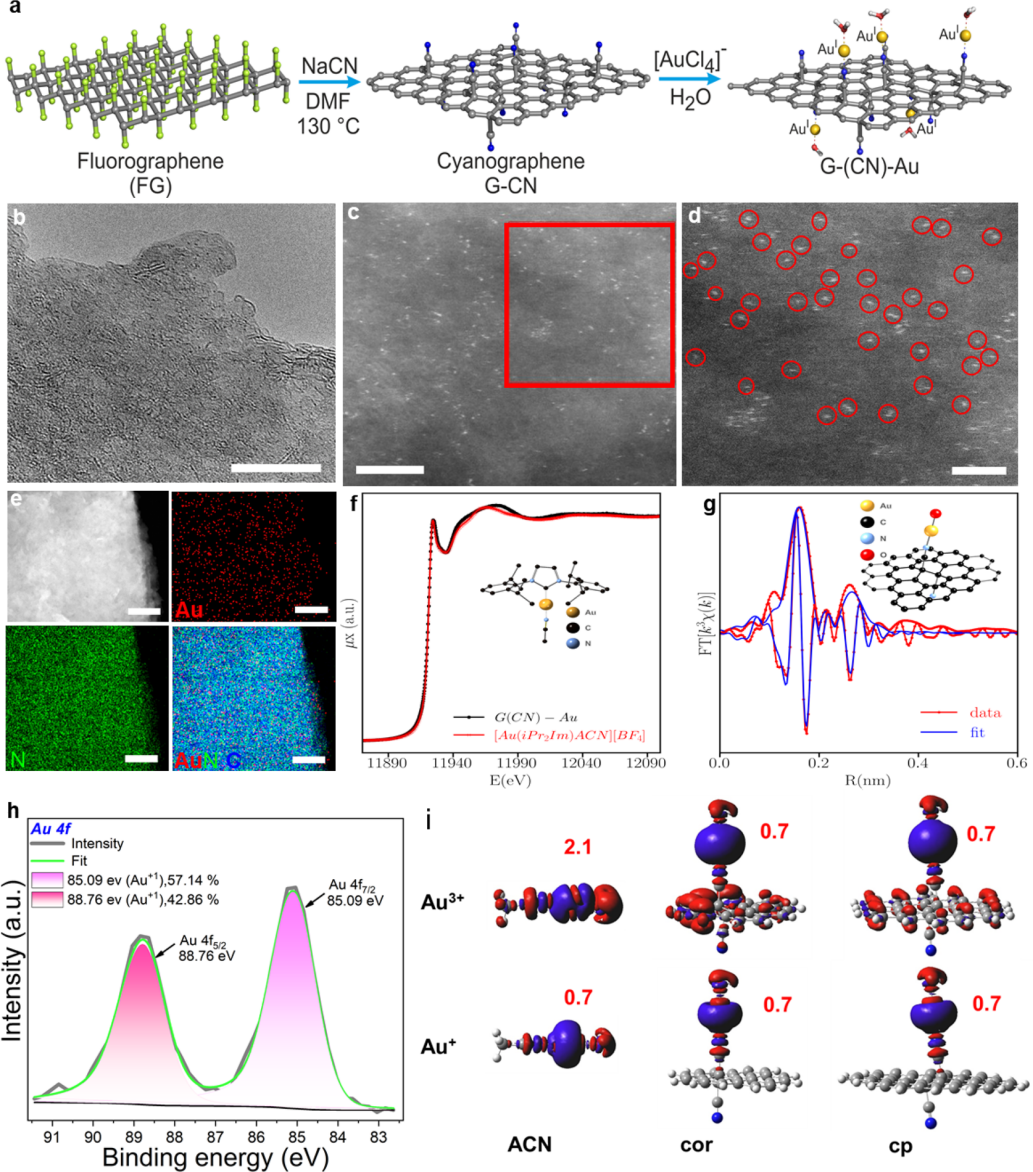
Characterization of the as-prepared catalysts. (a) Schematics of the preparative strategy for single-atom G(CN)-Au catalysts. (b) TEM image of G(CN)-Au (scale bar of 10 nm). (c) Representative high-angle annular dark-field scanning transmission electron microscopy (HAADF-STEM) images from the catalyst showing high-contrast spots from embedded single-atom gold; scale bar: 5 nm. (d) Magnified HAADF-STEM images depicting the presence of single Au atoms (highlighted by a red circle); scale bar: 2 nm. (e) HAADF image of G(CN)-Au and the corresponding energy dispersive X-ray spectroscopy (EDS) chemical mapping of G(CN)-Au displaying the dispersion of Au (red), N (green), and an overlap of Au (red), N (green), and C (blue), respectively; scale bar: 9 nm. (f) Normalized Au L_3_-edge X-ray absorption near-edge structure (XANES) spectra of G(CN)-Au and [Au(iPr_2_Im)ACN][BF_4_] (acetonitrile) confirming the Au(I) linear structure. (g) Fourier-transformed k^2^-weighted extended X-ray absorption fine structure (EXAFS) signal of G(CN)-Au and of the data fitting on the base of the model in the inset. (h) HR-XPS of the G(CN)-Au catalyst proving the exclusive presence of Au(I). (i) Results of DFT calculations: Electron density difference (EDD) plots and natural charges on Au atoms for Au^3+^ and Au^+^ ions (coordinated by a single water molecule) bound to acetonitrile (ACN), cor-2CN (cor = coronene), and cp-2CN (cp = circumpyrene). The red/blue regions in the EDD plots (contour isovalue: 0.003 au) correspond to the decrease/increase of the electron density in the complex with respect to isolated subsystems (i.e., R-CN and Au^*x*+^).

G(CN)-Au was synthesized by simple mixing of an aqueous solution of the HAuCl_4_ (Au(III)) precursor with G-CN dispersed in water without adding any reducing or stabilizing agent, leading to Au SA entrapment on the G-CN sheets (Figure S1) as depicted in the scheme (Figure 2a); the multigram-scale preparation exploits the abundant cyano-functionalities. Powder X-ray diffraction (PXRD) pattern analysis (Figure S2) confirmed the absence of any diffraction peaks assignable to Au NPs, signifying the SA character of gold species.

To probe the existence of Au single sites in G(CN)-Au, TEM (Figure 2b) and high-angle annular dark-field- scanning transmission electron microscopy (HAADF-STEM) was performed (Figure 2c,d); Au SAs are predominantly visible (Figure 2d, highlighted by red circles). Energy-dispersive X-ray spectroscopy (EDS) mapping (Figure 2e) also affirmed the homogeneous dispersion of single Au(I) atoms within the G-CN sheets. The valence state (+1) and coordination environment of single atom sites of gold were further confirmed by X-ray absorption fine structure spectroscopy (XAFS), X-ray absorption near-edge structure (XANES) spectroscopy, electron paramagnetic resonance spectroscopy (EPR), X-ray photoelectron spectroscopy (XPS), and corroborated by DFT calculations.

X-ray absorption near-edge structure (XANES) spectrum of the G(CN)-Au sample (Figure 2f) upon comparison with that of relevant standard compounds suggests that the G(CN)-Au sample contains essentially Au(I). Among the reference materials, a strikingly good match of XANES spectra (Figure 2f) is found only for an Au(I) compound where gold is coordinated to two light atoms with one being N from a linearly coordinated acetonitrile: [Au(iPr_2_Im)ACN][BF_4_] [(acetonitrile) [1,3-bis(2,6-diisopropylphenyl)imidazol-2-ylidene] gold(I) tetrafluoroborate] provided by Sigma-Aldrich.

The coordination of Au(I) to N from a CN group of G-CN is perfectly consistent with what has previously been proposed for Pt^31^ and Ag.^32^ Refinement of the EXAFS signal of the G(CN)-Au sample based on the structure sketched in the inset of Figure 2g (Figure S3) confirmed that the Au environment is close to that of the reference material with two atoms of light elements directly coordinated to the Au center. Assuming that Au(I) coordinated to N of a CN group in G-CN, a reasonably good fit of the first shell of FT k^2^-weighted EXAFS signals of G(CN)-Au (Figure 2g) is obtained with another light atom in the opposite position of the cyano group. While it is impossible to exclude the attribution to C or N as well, the best fit was obtained by introducing an O atom, reasonably from coordinated H_2_O or OH^–^ ions. The results of the fitting of the FT-k^2^ weighted EXAFS spectrum of G(CN)-Au are summarized in Table S1.

To perform the fit, coordination numbers have been fixed to unity; nevertheless, results are always close to these values if the fit is performed without restrictions. Notably, the Au–N distance is close to that found in the literature for similar compounds,^33^ where the Au–N (from acetonitrile) distance comprised between 0.196 and 0.200 nm. The C–N distance of the cyano group can be obtained, fitting the second shell of the FT-k^2^ weighted EXAFS spectrum, estimating a C–N distance, assuming a perfect linear coordination, of 0.113(4) nm in line within the experimental error to the value reported in the literature, that is, 0.113 nm.^34^ Multiple scattering paths originating from the cyano group coordination have been included and contribute to matching the FT of the EXAFS spectrum up to the apparent distance of 0.3 nm (Figure 2g).

The survey XPS spectrum (Figure S4) and binding state of G(CN)-Au, as investigated by X-ray photoelectron spectroscopy (XPS), engrained the presence of the two peaks with binding energies of 85.09 and 88.76 eV, corresponding to 4f_7/2_ and 4f_5/2_ to gold in an oxidation-state Au(I)^35^ (Figure 1h). The reducing capacity of G-CN is computationally manifested by natural population analysis, electron density difference (EDD) plots, and frontier molecular orbital (MO) analysis (Figure 1i) (Figures S5 and S6 and Tables S2 and S3) for model systems (in their singlet states) ACN-Au(I/III)-H_2_O (ACN = acetonitrile), cor-2CN-Au(I/III)-H_2_O (cor = coronene), and cp-2CN-Au(I/III)-H_2_O (cp = circumpyrene) representing aliphatic (ACN) and π-conjugated substrates (cor and cp); the triplet state of G(CN)-Au(III) in water was found to be less stable than the singlet state by ca. 18 kcal/mol.^36^ Whereas in the case of ACN-Au(III)-H_2_O, the partial charge on the gold atom is approximately +2.1e, it decreases to +0.7e for π-conjugated substrates, thus reaching the value corresponding to Au(I) sites. A similar role of G-CN was observed in the case of the G(CN)-Cu system (Figure S6), wherein the G-CN substrate reduced some of the Cu(II) sites to Cu(I), resulting in a mixed-valence copper catalyst.^37^ Analysis of the HR-XPS N 1s envelope experimentally corroborated the charge transfer and the interaction between the N atoms from G-CN and Au cations, as explained in (Figure S7a). To conclude this part, the G-CN substrate is clearly capable of efficient binding of single Au cations and stabilizing the linear structure of the Au(I) state by surface-to-metal charge transfer.

### Selective Dehydrogenative Coupling of Organosilanes

Dehydrogenative coupling of organanosilanes with alcohol was explored to evaluate the efficiency of various Au-based catalysts compared to G(CN)-Au. The synergic effect of the Au(I) species and the G-CN platform constituting the G(CN)-Au SAC was demonstrated by performing the reaction in the absence of the catalyst in the presence of G-CN without Au as well as using widely deployed homogeneous Au-based catalysts, that are, Au(I) salts (PPh_3_)AuCl, [(iPr)AuCl], and Au(III) acid HAuCl_4_ (Table S4, entries 1–5). Essentially, the reactions without the catalyst with sole G-CN as well as in the presence of Au(I) salts or HAuCl_4_ showed nearly no conversion. Apparently, the inactivity of Au(III) species can be due to further easy oxidation, which is a mandatory step for Si–H bond activation. On the other hand, the failure of Au(I) homogeneous salt performance implies that the G-CN-bound Au(I) active sites significantly enhanced the catalytical activity. These observations validate that the Si–H bond activation of silanes occurs on the surface of the solid G(CN)-Au catalyst.^2,38^

The deployment of G(CN)-Au for the catalytic reaction was realized via threefold sequential experiments for each reaction to ensure the reaction uniformity with consideration of the average conversion values of individual experiments. The loading of gold was confirmed with inductively coupled plasma mass spectrometry (ICP-MS) amounting to ∼0.6 wt %. Under the test conditions, the designed G(CN)-Au SAC offered equivalent alkoxysilane conversion (>99%) and selectivity (99%) (Table S4, entry 6) quantitatively reaching the highest TOF up to 96,378 h ^–1^ (Figure S8a,b) based on total gold content. To investigate the intrinsic role of catalytically active Au(I) species, G(CN)-Au was treated under the H_2_/Ar atmosphere at 200 °C, leading to a reduced catalyst referred to as G(CN)-Au-R, which was characterized with TEM, XPS, and XRD (Figure S9). The XPS results (Figure S9c) revealed that only 38% of Au(I) remained after the reduction and most of Au(I) converted into Au(0). Further, the G(CN)-Au-R catalyst afforded conversion only up to 20% (Table S4, entry 7) for the reaction signifying the key role of the Au(I) species in the catalytic process.

To compare the G(CN)-Au with the broadly utilized graphene oxide (GO) as a carbon-based support, GO-supported gold (Au/GO) was prepared using an analogous procedure (see the Supporting Information). However, GO could not stabilize Au in the Au(I) oxidation state, and zero-valent gold was formed, as confirmed with TEM, HR-XPS, and XRD (Figure S10). Therefore, under the optimized conditions, Au/GO showed only trace conversions and GO displayed no conversions at all (Table S4, entries 8 and 9). The highly active single atomic Au(I) sites and their uniform dispersion in G(CN)-Au are thus the two key factors for the excellent performance of this catalyst in the dehydrogenative coupling of silanes with alcohols. This catalyst even surpasses the performance of almost all reported catalysts for the dehydrogenative coupling of organosilanes with alcohols in the literature (Table S5).

### Substrate Scope for Selective Dehydrogenative Coupling of Organosilanes and Alcohols

To investigate the general applicability of the G(CN)-Au catalyst, we selected important aromatic organosilanes to react with several primary alcohols under the developed optimized conditions. Notably, dimethylphenylsilane upon coupling with various alcohols to the corresponding silyl ethers showed uniquely high conversion and selectivity as high as >99% in a short time (Table 1, entries **2a**–**2c**, **2g**, and **2h**) and attained 1 order of magnitude higher of a TOF of up to 139,494 h^–1^ (based on the total Au content) than reported top catalysts (Table S5). Diphenylsilane with two Si–H bonds efficiently underwent selective monoalcoholysis to afford monoalkoxy silylethers with high conversion while retaining selectivity even under a prolonged reaction time toward monoalcoholysis, thus demonstrating the excellent feature of G(CN)-Au relative to previously reported catalysts^39^ (Table 1, entries **2g** and **2h**). Furthermore, bulky organosilanes such as triphenylsilane worked equally well and furnished the corresponding silyl ethers with excellent conversion and selectivity (Table 1, entries **2i**–**2k**). To shed more light on the catalytic activity of G(CN)-Au, silanes with three Si–H bonds were allowed to react with primary alcohols, which turned out to be very reactive thus affording dialkoxysilane (Table 1, entry **2la**) and trialkoxysilane (Table 1, entries **2lb**, **2m**, and **2n**) with >99% conversion and excellent selectivity comparable to previous reports.^40^ G(CN)-Au also performed well for etherification of alicyclic silanes with alcohols to give excellent conversion and selectivity of the corresponding ethers (Table 1, entries **2o**–**2q**).

**Table 1:**
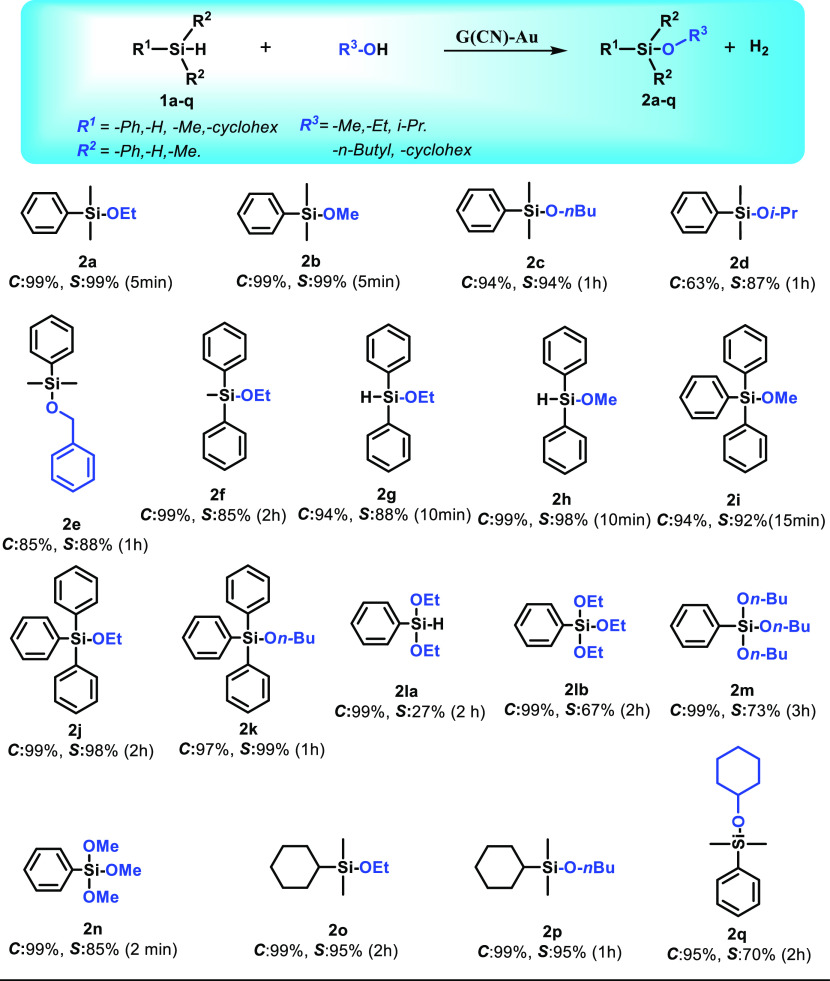
Single-Atom G(CN)-Au-Catalyzed Dehydrogenative Coupling of Organosilanes with Alcohols^a^

a Reaction conditions: 1 mmol substrate (silane) and 10 mg of G(CN)-Au (0.6 wt % based on Au) dispersed in 1.5 mL of alcohols at 25 °C. Conversions (*C*) and selectivities (*S*) were determined and confirmed by GC and GC-MS. The selectivity was determined upon the maximum conversion monitored by GC and GC-MS.

The potential activity of G(CN)-Au encouraged us to investigate the scope of the reaction for alkoxysilanes, which are highly demanded as significant intermediates in polymer chemistry as well as coupling agents for natural fibers.^41^ Triethylsilane upon a reaction with different alcohols offered respective products with high selectivity and conversion reaching up to >99% (Table 2, entries **3a**–**3e**). Long-chain alkylsilanes such as butylsilane also efficiently provided the selective products dialkoxybutylsilanes (Table 2, entries **3fa**, **3g**, and **3h**) and trialkoxybutylsilanes (Table 2, entry **3fb**). Due to the very low boiling point of butylaniline, it was hard to identify small quantities of unreacted butylsilane; in the case of butylsilane, we continued the reaction for a longer time to achieve maximum conversion. Hexylsilane also underwent a reaction to provide dialkoxyhexylsilanes (Table 2, entry **3ja**) and trialkoxyhexylsilanes (Table 2, entries **3i** and **3jb**), which are important precursors with extensive applicability as inorganic surface modifiers via a hydrolysis process in assorted advanced fields. Likewise, bulky organosilanes, namely, dimethyloctadecylsilane and trioctylsilane, were smoothly converted into desired products (Table 2, entries **3k**–**3p**). Silanols are important building blocks and are used as monomers for silicone polymers,^42^ often requiring stoichiometric amounts of strong oxidants such as permanganate,^43^ osmium tetroxide,^44^ and peracids,^45^ which generate siloxanes and other toxic and detrimental byproducts.^46^ Recently, several Au-based heterogeneous catalysts, for example, Au_1_/mpg-C_3_N_4_^2^_,_ KCC-1-APTS/Au,^25^ AuCNT,^47^ and Au/SiO_2_,^26^ have also been explored for this transformation, but the limited substrate scope, longer reaction times, and preparative batch methods restrict their applicability. Next, the activity of G(CN)-Au toward oxidation of organosilanes with water was explored further by choosing dimethylphenylsilane as a substrate; G(CN)-Au again manifested high activity and afforded corresponding silanol with conversion of >99% and selectivity of >99% (Table S6, entry **5a**). To sum up, G(CN)-Au is an ideal catalyst for the transformation of most of the prominent organosilanes into silanol (Table S6).

**Table 2:**
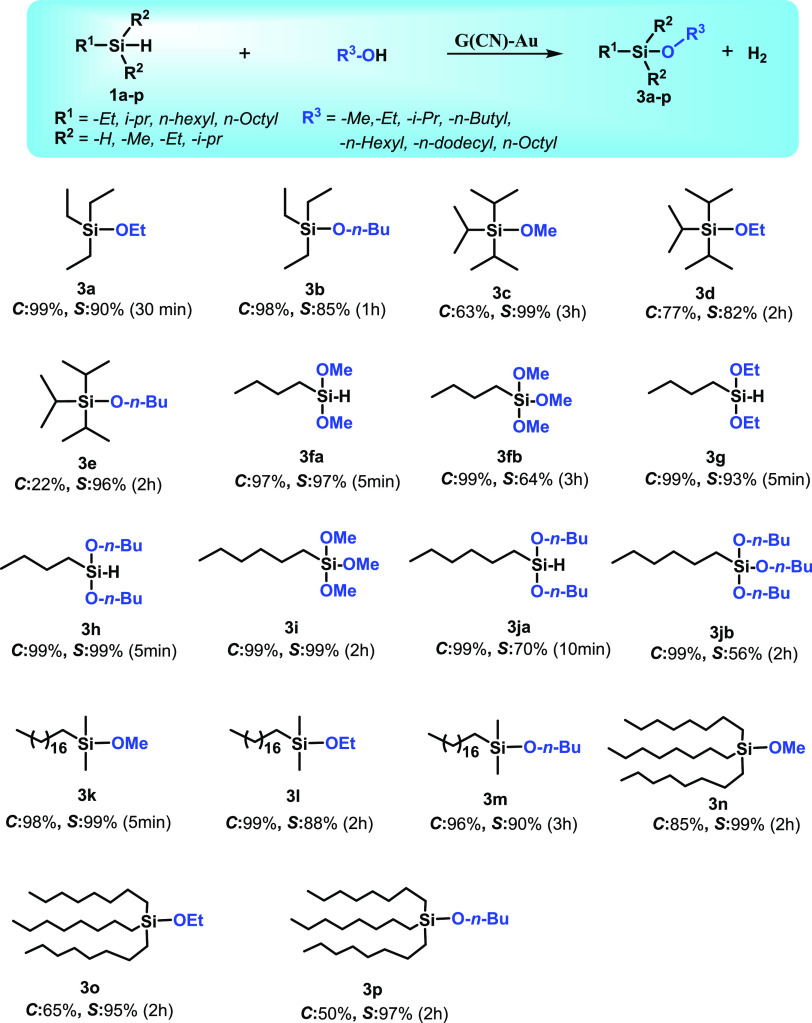
Single-Atom G(CN)-Au-Catalyzed Dehydrogenative Coupling of Long-Chain Organosilanes with Alcohols^a^

a Reaction conditions: 1 mmol substrate (silane) and 10 mg of G(CN)-Au (0.6 wt % based on Au) dispersed in 1.5 mL of alcohols at 25 °C; percentage conversions (*C*) and selectivities (*S*) were determined and confirmed by GC and GC-MS. Selectivity was determined upon the maximum conversion monitored by GC and GC-MS.

To validate the G(CN)-Au, a catalyzed gram-scale approach for the oxidation of dimethylphenylsilane was conducted. For example, a 10 mmol batch produced the corresponding product with excellent conversion and selectivity (Figure S11), and the amount of H_2_ generated during the reaction was measured using the water displacement method, employing an inverted measuring cylinder (Video S1). Furthermore, to broaden the scope of applicability, the batch reaction was effectively extended to a continuous flow process; reactions proceeded remarkably well in very excellent conversions and selectivities for both the dehydrogenative coupling of various silanes with alcohols (Table 3, entries **4a**–**4d**). G(CN)-Au remained stable almost for four consecutive cycles, indicating good recyclability and stability of the catalyst; a minor drop in activity after the third cycle necessitated a longer reaction time for full conversion. The recyclability study for G(CN)-Au catalysts, as shown in Figure S12a, encompassed a thorough characterization of the recycled catalysts (Figure S12b–g). The stability of G(CN)-Au was also appraised under a continuous flow by performing the reaction for a longer time (25 h). The reaction product was collected and evaluated every 5 h to investigate the change in conversion and selectivity (Figure S13). It is evident from the results that the catalyst was significantly active even after 25 h, thus showing a stable nature. However, a variation in conversion and selectivity was observed during the course of time that could be due to the many reasons such as reduction of the Au(I) active site to Au(0), which is less active, covering active sites and leaching active sites under a continuous flow.^48,49^ The catalyst has exhibited better stability in terms of recyclability under batch than the flow reactions for the long run, but the results of batch and flow reactions cannot be compared as they differ in terms of various parameters (contact time, surface-to-volume ratio, mixing, heat and mass transfer, and autogenous pressure).^50^ These parameters can significantly alter not only the rate of the reaction but also the selectivity of the reaction.

**Table 3:**
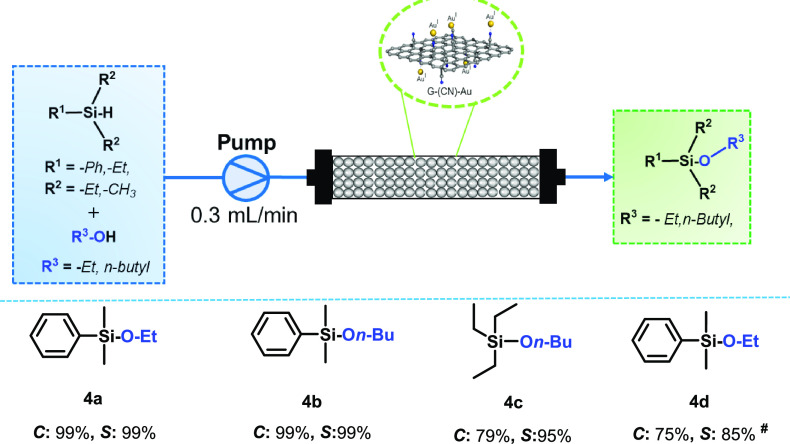
Single-Atom G(CN)-Au-Catalyzed Dehydrogenative Coupling of Organosilanes with Alcohols Employed under the Continuous-Flow Reaction Mode^a^

a Reaction conditions: 30 mg of the G(CN)-Au catalyst placed in a catalyst holder size (24 mm × 4 mm), 60 μL of the substrate in 40 mL of solvent, 0.3 mL/min flow rate, 10 min under autogenous pressure, 50 °C. Conversions (*C*) and selectivities (*S*) were determined and confirmed by GC.

b Reaction streamed for 25 h.

### Insights from EPR Spectroscopy Measurements

**Figure 3:**
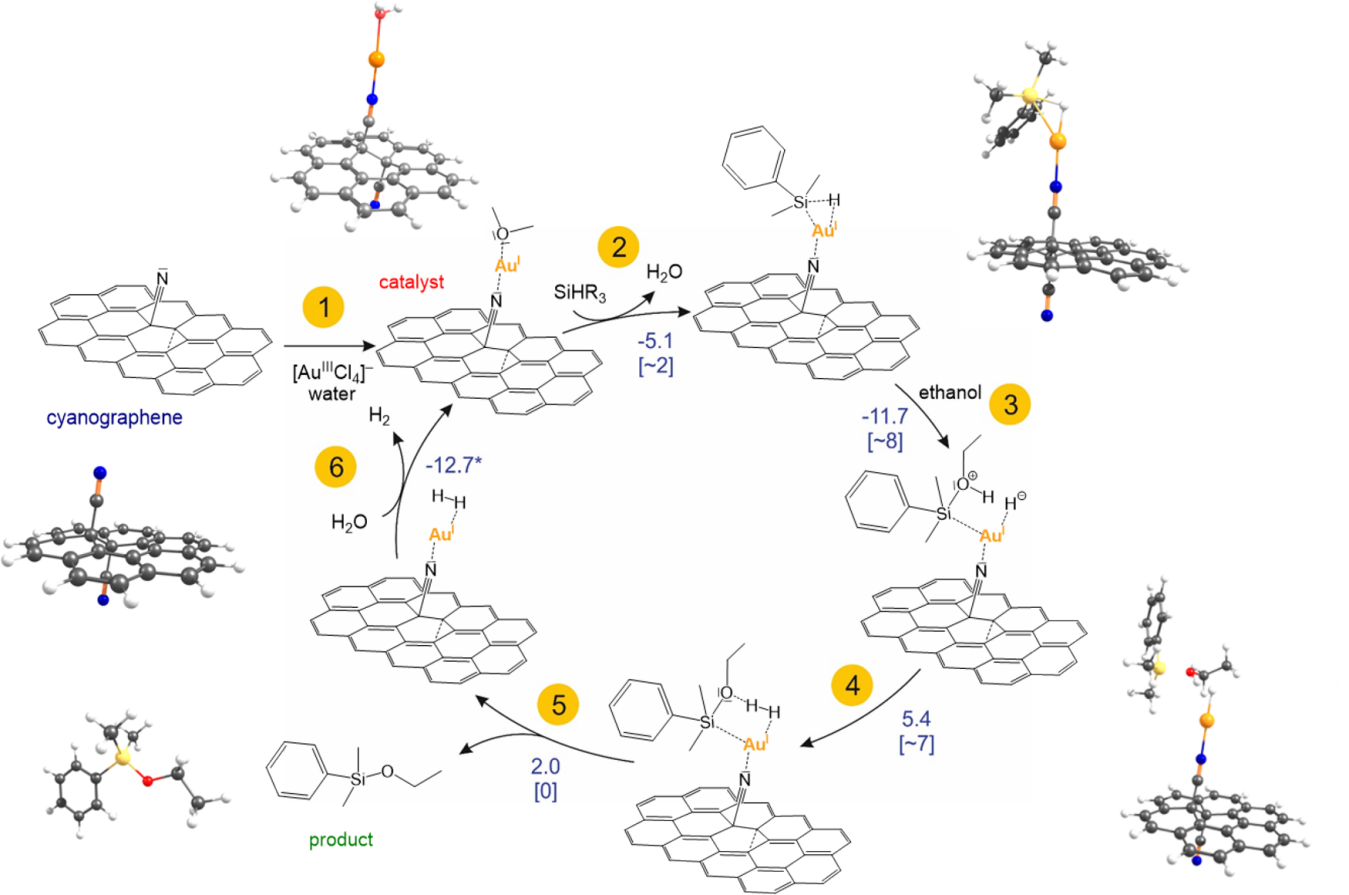
Proposed mechanism for the dehydrogenative coupling of silanes with alcohols catalyzed by G(CN)-Au. Reaction energies (in kcal/mol) and activation barriers (in parentheses) were obtained at the PBE0/DEF2-TZVP/SMD level of theory. The value for step 6 includes an estimated entropic contribution (see text). Energy profiles related to the mechanism are presented in Figures S23–S28. Key optimized model structures are displayed along the cycle (carbon atoms are gray, nitrogen is blue, hydrogen is white, oxygen is red, gold is orange, and silicon is yellow).

The detailed electronic/magnetic characteristics of the G(CN)-Au SACs were analyzed by the EPR spectroscopic technique as explained in Figures S14 and S15–S17. The EPR signal of neat G-CN is given in Figure S14A(b) and exhibits a strong resonance signature centered at a *g* value of 1.9960. This signal arises from the presence of spin-containing defects embedded in the organic G-CN support. The spin density of neat G-CN evaluated against a CuSO_4_ standard accounts for 2.0 × 10^19^ spin/g. After incubation of G-CN with Au(III) cations, the spin density of the so-formed catalyst G(CN)-Au falls to 1.57 × 10^19^ spin/g. Thus, a substantial reduction (∼21%) of the spin concentration is observed in the active catalyst compared to neat G-CN. The result can be interpreted in terms of an electron transfer process occurring from the G-CN support to the entrapped Au(III) cations during synthesis of G(CN)-Au, forming Au(I) sites ([Xe]4f^14^5d^10^) coordinated to the CN groups present in the organic support. To probe the changes, if any, in the EPR features of the G(CN)-Au after catalysis, the material was sequentially collected after one, two, and three reaction cycles and dried each time from the reaction mixture ((Figures S14B and S18–S20). After each cycle, the EPR signal associated with the broad wings developing underneath the strong signature at *g* = 1.9960 appeared to increase. Taking into consideration the EPR features shown in Figure S14A(a) in which the presence of small Au(0) nanoclusters in HAuCl_4_ salt gives weak and broad EPR resonance features at *g* of ∼2.00, we evoke that, in G(CN)-Au, the witnessed increase upon recycling of the broad wings mirrors the slow accumulation of small Au(0) clusters. It should be noted that only the former Au(I) sites may function as catalytically active centers in the H-abstraction process from silyl substrates because only such sites can facilitate the Au(I)···H^–^ interaction (see step 3 in Figure 3).

### Theoretical Insights into the Reaction Mechanism

Based on the experimental observations and DFT calculations, a sequential reaction mechanism for the oxidative coupling of silanes with alcohols involving the G(CN)-Au SAC can be proposed (Figure 3). In the first step, G(CN)-Au is formed via a redox reaction of [Au(III)Cl_4_]^−^ anions with the G-CN substrate, which functions as an efficient reducing agent due to its high electron-donor strength arising mainly from high-lying π orbitals spread over the graphene lattice. Single-ion linear-structure Au(I) centers of the G(CN)-Au catalyst represent suitable binding sites for dimethylphenylsilane (DMPS).

The binding affinity of DMPS to G(CN)-Au(I)-H_2_O (step 2) is equal to −5.1 kcal/mol with a very small activation barrier (∼2 kcal/mol) related to the release of a coordinating water molecule (Figure S23). Importantly, the Si–H bond in the formed G(CN)-Au···DMPS complex is notably longer (1.77 Å) compared to that in DMPS (1.49 Å), indicating the weakening (i.e., activation) of the Si–H bond, which is one of the key factors for the catalytic process. Simultaneously, the hydrogen atom interacts with the Au(I) site (R(Au–H) = 1.61 Å), thus forming a suitable configuration for a later reaction with alcohol (step 3). The ethanol molecule preferentially binds directly to silane with the BE equal to −11.7 kcal/mol and the activation barrier of ∼8 kcal/mol (step 3; Figures S24–S27). A positively charged ethanol–silane complex can be deprotonated with a low activation barrier (∼7 kcal/mol), leading to the formation of an intermediate with the hydrogen molecule bound to the Au(I) site (step 4; Figure S28). Both the product and hydrogen molecules are subsequently released in energetically feasible steps 5 and 6 to recover the catalyst (Figure 3). As the evolution of hydrogen gas is connected with significant changes of entropy, we estimated the entropic contribution to Gibbs reaction energy assuming that the dominant entropy change is related to translation and rotational degrees of freedom of the formed hydrogen molecule. At room temperature, the corresponding entropic contribution is −9.5 kcal/mol, which brings down the reaction energy from −3.4 to −12.7 kcal/mol. Taking into account the fact that hydrogen is continuously released from the reaction mixture (Video S1), this step is highly favorable. Based on the EPR and XPS analyses, a minor decrease in the catalytic efficiency of G(CN)-Au after few cycles was attributed to the presence of zerovalent Au nanoclusters, which did not appear to be catalytically active. To corroborate these findings, the binding of a model Au_20_ nanocluster on the graphene substrate was also modeled by combining the periodic boundary condition (PBC) and finite-size DFT calculations (Figure S29 and Table S7), showing that the Au nanoclusters represent a stable form on the G-CN lattice, but the reaction of silane with ethanol is sterically unfavorable, and thus the catalytic process predominantly takes advantage of the presence and nature of the single Au(I) ions occupying the nitrile groups of G-CN.

## Conclusions

In summary, the linear-structure single-atom G(CN)-Au catalyst was first developed via a simple and cost-effective method under mild conditions. We have illustrated that the G-CN support not only functions as a chemically active reducing platform stabilizing the valence state of a single gold atom (+1) but also enables the efficient dispersion and stabilization of catalytic Au(I) sites in the structure of the two-dimensional graphene matrix, thus providing an extremely favorable arrangement for the catalytic performance of the catalyst. The catalytic Au(I) sites were proven to be highly active in the Si–H bond activation, resulting in efficient dehydrogenative coupling of organosilanes with alcohols and water to silyl ethers and silanol, respectively, with record TOF values among all reported catalysts. The reaction mechanism was explored by exploiting both experimental and theoretical data. It was proven that a high electron donor strength arises mainly from high-lying π orbitals spread over the graphene lattice. Moreover, single-ion linear-structure Au(I) centers of the G(CN)-Au catalyst represent favorable binding sites for dimethylphenylsilane.

Importantly, the ensuing products are in high demand as an important backbone in polymer chemistry, while generating hydrogen as a clean byproduct renders the protocol highly atom economic and cost-effective. Additionally, the strategy exploiting the chemically active 2D graphene support for chemical control of SA electronic properties and contributing to the unique coordination environment with a linear structure of metal ions can be efficiently applied on a large scale and under continuous flow appliance, which trigger their applicability in versatile reactions mainly due to the superior affinity of such linear coordination to reactants. We anticipate that the strategy employed to design a linear-structure single-metal atom G(CN)-Au catalyst can be extended to design analogous SACs for other catalytic applications relying on specific valence states of single atoms in the unique coordination of SAs.

## Supporting Information Available

Water displacement method employing an inverted measuring cylinder measuring the amount of H_2_ generated in the reaction (MP4)
Detailed experimental procedures of catalyst preparation, characterization, catalytic evaluation, computational methods, additional data, corresponding discussion, and supporting references (PDF)
