# Supplementary material for: Linear-Structure Single-Atom Gold(I) Catalyst for Dehydrogenative Coupling of Organosilanes with Alcohols
Source: ACS Catal. 2023 Nov 30;13(24):16067–77. doi: 10.1021/acscatal.3c03937 (PMC10729017; doi:10.1021/acscatal.3c03937)
Supplement: Supplementary file 2 — cs3c03937_si_002.pdf [file cs3c03937_si_002.pdf]

Supporting Information for

Linear-Structure Single-Atom Gold(I) Catalyst for Dehydrogenative Coupling of Organosilanes with Alcohols

Ravishankar G. Kadam,[1] Miroslav Medveď,[1,2] Subodh Kumar,[3] Dagmar Zaoralová,[1,4] Giorgio Zoppellaro,[1,5] Zdeněk Baďura,[1,5] Tiziano Montini,[6] Aristides Bakandritsos,[1,5] Emiliano Fonda,[7] Ondřej Tomanec,[1] Michal Otyepka,[1,4] Rajender S. Varma,[1] Manoj B. Gawande,*[1,8] Paolo Fornasiero,*[6] Radek Zbořil*[1,5]

[1]Regional Centre of Advanced Technologies and Materials, Czech Advanced Technology and Research Institute, (CATRIN), Palacký University Olomouc, Šlechtitelů 27, 779 00 Olomouc, Czech Republic. E-mail: radek.zboril@upol.cz

[2]Department of Chemistry, Faculty of Natural Sciences, Matej Bel University, Tajovského 40, 974 01 Banská Bystrica, Slovak Republic

[3]Department of Inorganic Chemistry, Faculty of Science, Palacký University Olomouc, 17. listopadu 12, 779 00 Olomouc, Czech Republic.

[4]IT4Innovations, VŠB–Technical University of Ostrava, 17. listopadu 2172/15, 708 00 Ostrava- Poruba, Czech Republic.

[5]CEET, Nanotechnology Centre, VŠB–Technical University of Ostrava, 17. listopadu 2172/15, 708 00 Ostrava-Poruba, Czech Republic.

[6]Department of Chemical and Pharmaceutical Sciences, Center for Energy, Environment and Transport Giacomo Ciamician, INSTM Trieste Research Unit and ICCOM-CNR Trieste Research Unit, University of Trieste via L. Giorgieri 1, I-34127 Trieste, Italy, E-mail: pfornasiero@units.it

[7]Synchrotron SOLEIL, L'Orme des Merisiers, 91190 Saint Aubin, France

[8]Department of Industrial and Engineering Chemistry, Institute of Chemical Technology, Mumbai-Marathwada Campus, Jalna, Maharashtra 431213, India.
Email : mb.gawande@marj.ictmumbai.edu.in

Table of Contents

1. Experimental section…………………………………………………………………..3

1.1 Materials and chemicals…………………………………………………………….. …3

1.2 Catalysts preparation……………………………………………………………………3

Synthesis of Cyanographene (G-CN)…………………………………………………..3

Synthesis of G(CN)-Au…………………………………………………………………...3

Synthesis of Au/GO……………………………………………………………………....4

2. Characterization details..5

2.1 Computational details...7

3. Supplementary tables...9

4. Supplementary figures………………………………………………………………..16

5. Supplementary video…………………………………………………………………50

6. NMR and Mass spectra of compounds..51

7. References 72

1. Experimental section

1.1 Materials and chemicals

Graphite fluoride (>61 wt.% F, $C_1F_{1.1}$), NaCN (p.a. ≥97%), *N*,*N*-Dimethylformamide (≥99.8%), $HAuCl_4$ (99.99%), $(Ph_3P)AuCl$, IprAuCl, Na_2SO_4 anhydrous (≥99%), ethyl acetate anhydrous (99.8%), 1,4-Dioxane anhydrous (99.8%), Graphene oxide (GO) (C112/GOB013/Pw), all aromatic silanes, were purchased from Sigma Aldrich and TCI chemicals used as received without further purification. All solvents were HPLC grade. All aqueous solutions were prepared with ultrapure water (UPW) (18 MΩ cm^{-1}).

1.2 Catalysts preaprations

Synthesis of Cyanographene (G-CN)

Cyanographene was synthesized by following our previously described method.[1] Briefly; fluorinated graphite (120 mg, ~4 mmol of C-F units) was added to 15 mL of DMF and stirred for 2 days. Then sonicated (Bandelin Sonorex, DT 255H type, frequency 35 kHz, power 640 W, effective power 160 W) for 4 h under nitrogen atmosphere. Then 800 mg of NaCN (~16 mmol) was added and the mixture was heated at 403 K with a condenser under stirring (500 rpm). After 2 days, the mixture was left to cool to room temperature. After washing and isolation of the pure product, the material was suspended in UPW.

Synthesis of G(CN)-Au:

The G(CN)-Au was prepared by adding the 0.012 M $HAuCl_4$ (0.5 ml) into the G-CN aqueous dispersion (100 mg G-CN dispersed in 20 mL of UPW and sonicated for 10 minutes). The corresponding mixture was stirred at room temperature for 24 h. The resulting material was separated via centrifugation at 21000 RCF for 15 min, then mixture was washed twice with UPW and centrifuged to isolate the final gold-loaded GCN. Finally, the G(CN)-Au was re-dispersed in 4 ml of UPW and freeze dried and stored for further use. The determination of the gold content in the solid catalysts performed by inductively coupled plasma mass spectrometry (ICP-MS). The BET surface area of G(CN)-Au catalyst was found to be 96 ± 0.8 m^2/g.

Synthesis of Au/GO

The graphene oxide (GO) loaded gold, material was prepared by identical procedure as used for G(CN)-Au. Brifely, the GO aqueous dispersion (100 mg GO dispersed in 20 mL of UPW and sonicated for 10 minutes). The corresponding mixture was stirred at room temperature for 24 h. The resulting material was separated via centrifugation at 21000 RCF for 15 min, then mixture was washed twice with UPW and centrifuged to isolate the final gold-loaded Au/GO. Finally, the G(CN)-Au was re-dispersed in 4 ml of UPW and freeze dried and stored for further use.

Representative procedure for dehydrogenative coupling of organosilane with alcohol

In the typical experimental procedure, a 10 ml vial was charged with dimethylphenylsilane (1 mmol), 1.5 ml ethyl alcohol and 10 mg of G(CN)-Au (0.6 *wt*%) and the resulting reaction mixture was stirred at 25 °C for 5 min. thereby yielding the desired dimethylethoxyphenylsilane product. The progress of the reaction was monitored with gas chromatography (GC) and gas chromatography-mass spectrometry (GC−MS). After completion of the reaction, catalyst was isolated by centrifugation. For ^{1}H and ^{13}C NMR analysis products were purified by column chromatography on silica gel with anhydrous ethyl acetate/hexane mixture followed vacuum drying process.

Representative procedure for dehydrogenative coupling of organosilane with water

In the typical experimental procedure, a 10 ml vial was charged with dimethylphenylsilane (1mmol), 2 ml acetone, 0.1 ml water and 10 mg of G(CN)-Au (0.6 *wt*%) and the resulting reaction mixture was stirred at 25 °C, thereby yielding the desired dimethylphenylsilanol product. The reaction progress, conversion and selectivity were determined and confirmed by GC-FID and GC-MS.

Representative procedure for oxidation of alkyl silane with alcohol

In the typical experimental procedure, a 10 ml vial was charged with triethyl silane (1mmol), 2ml ethyl alcohol and 10 mg of G(CN)-Au (0.6 *wt*%) and the resulting reaction mixture was

stirred at 25 °C for 5 min., thereby yielding the desired ethoxytriethylsilane product. The reaction progress was monitored with GC-FID and GC-MS.

Representative procedure for gram scale dehydrogenative coupling of organosilane with alcohol

In the typical experimental procedure, a 100 ml round botton flask was charged with Dimethylphenylsilane (10 mmol), 20 ml ethyl alcohol and 100 mg of G(CN)-Au and reaction mixture was stirred at 25 °C. The reaction progress was monitored with GC-FID and GC-MS.

Representative procedure for dehydrogenative coupling of silanes with alcohol under continues flow

In the typical experimental procedure, 30 mg of G(CN)-Au catalyst placed in a catalyst holder size (24 mm x 4 mm) and 60 µl dimethylphenylsilane in 40 ml ethanol was flown through the column under autogenous pressure for 10 min. The reaction progress was monitored by GC-FID and GC-MS.

TOF calculation

The TOFs were calculated based on the conversion, selctivity of silanes and total loading of gold. Dispersity of single atomic surface gold species considered as 1.

TOF = [Mol(substrate)*(conversion*selectivity)]/[Mol(Au)*dispersity*reaction time (hour)]

2. Characterization details

X-ray diffraction (XRD) patterns for G-CN, G(CN)-Au and G(CN)-Au-R samples were measured on Aeris diffractometer, Co-Kα radiation: λ = 0.178901 nm, scanned in the 2θ range from 5° to 105°, step size 0.022° 2θ, 63 min in total. Au/GO sample was measured using Empyrean diffractometer, Co-Kα radiation: λ = 0.178901 nm, scanned in the 2θ range from 5° to 80°, step size 0.026° 2θ, 78 min in total.

Microscopic TEM images were obtained by HRTEM TITAN 60-300 with X-FEG type emission gun, operating at 80 kV. This microscope is equipped with Cs image corrector and a STEM high-angle annular dark-field detector (HAADF). The point resolution is 0.06 nm in

TEM mode. The elemental mappings were obtained by STEM-Energy Dispersive X-ray Spectroscopy (EDS) with acquisition time 20 min. For HRTEM analysis, the powder samples were dispersed in ethanol and ultrasonicated for 5 min. One drop of this solution was placed on a copper grid with holey carbon film. XPS surface investigation has been performed on the PHI 5000 VersaProbe II XPS system (Physical Electronics) with monochromatic Al-Kα source (15 kV, 50 W) and photon energy of 1486.7 eV. Dual beam charge compensation was used for all measurements. All the spectra were measured in the vacuum of 1.3×10^{-7} Pa and at the room temperature of 21 °C. The analyzed area on each sample was spot of 200 µm in diameter. The survey spectra were measured with pass energy of 187.850 eV and an electron-volt step of 0.8 eV while for the high-resolution spectra was used pass energy of 23.500 eV and electronvolt step of 0.2 eV. The spectra were evaluated with the MultiPak (Ulvac - PHI, Inc.) software. All binding energy (BE) values were referenced to the carbon peak C 1s at 284.80 eV. EPR spectra were recorded on JEOL JES-X-320 operating at X-band frequency (~ 9.0 - 9.1 GHz), equipped with a variable temperature control ES 13060DVT5 apparatus or with a CT470 liquid He variable temperature apparatus operating at T = 77 K. The cavity Q quality factor was kept above 5000 in all measurements. The powder materials ($HAuCl_4$ precursor, G-CN and G(CN)-Au) were loaded into polypropylene VSM holders (P125E) and inserted directly into highly-pure quartz tubes, and air was purged by fluxing with nitrogen gas (Suprasil, Wilmad, ≤ 0.5 OD). Spin density was evaluated by using the $CuSO_4 \times 5H_2O$ (99.999%, CAS Number: 7758-99-8), which was loaded into the VSM holder in powder form. Au L_3 edge of sample G(CN)-Au has been measured at SAMBA beamline of synchrotron SOLEIL[2] Pd mirrors have been set at 4mrad to reject harmonics, the monochromator was equipped with Si (220) crystals, measurements have been performed in fluorescence mode with a 35 pixels HPGe detector (Mirion/Canberra) in conjunction with DxMap DSP (XIA), while ionization chambers where filled with nitrogen at 1 bar (7.5% absorption at 12keV), the machine current was 16 mA (single bunch mode). Data analysis has been performed via Athena and Artemis[3] codes.

Measurements of reference materias have been performed in transmission. In order to refine EXAFS, a model structure has been built and relaxed with empirical potentials via the software CrystalMaker and then fed to FEFF in Artemis. The S_0^2 has been deduced from a fitting on a gold foil and set to 0.9 as usual. The metal content of G(CN)-Au catalyst metal loadings was determined using inductively coupled plasma mass spectrometry (ICP-MS) (Agilent 7700x, Agilent, Japan). The remaining Au was dissolved with freshly prepared aqua regia. The obtained solution was transferred to a volumetric flask and diluted with Milli-Q water, and the Au concentration was measured using ICP-MS. The reaction products conversion and selectivity were analyzed by gas chromatography (Model: Agilent 6820) equipped with an Agilent DB-5 capillary column (30 m × 0.32 mm, 0.5 m) and mass spectrometry (GC-MS). Nuclear magnetic resonance (NMR) spectra ^1H and ^{13}C NMR spectra of ensued compounds from the catalytic reactions were recorded on 400 MHz NMR Varian spectrometer (Varian, Santa Clara, CA, USA) and on an JNM-ECA600II NMR spectrometer (JEOL, Japan) at 298 K, using $CDCl_3$/DMSO-d_6 as a solvent.

2.1 Computational details

To analyze reaction energies, structural features, spin and charge distributions and other characteristics of species involved in the oxidative coupling of silanes with alcohols catalyzed by the G(CN)-Au catalyst, we performed density functional theory (DFT) calculations applying the PBE0 method[4] with the effective core potential (ECP) DEF2-TZVP basis set.[5] The choice of the exchange-correlation functional and the basis set was based on the benchmark study by Pasteka et al.[6] comparing the performance of a wide range of DFT functionals and various basis sets for evaluating the binding energies of coinage metals with lone-pair ligands taking the CCSD(T) results as the reference. For the open-shell system, the spin unrestricted formalism was applied. The solvent effects were included by using the universal continuum solvation model based on solute electron density (SMD).[7] While the structures of small species (H_2O,

ethanol, silane, product, …) were fully relaxed in geometry optimizations, to mimic the semilocal rigidity of graphene sheets, the G(CN)-Au and alike structures were obtained by constrained geometry optimizations keeping the edge carbon atoms fixed. The G-CN substrate was modeled as a finite-size system containing two cyanogroups bound to the central part of cor (Figure S1a). To check the appropriateness of the model size, the binding characteristics for the G(CN)-Au catalyst were also evaluated for a larger model derived from circumpyrene (cp) (Figure S1b). Optimized structures of Au(I) atoms coordinated by acetonitrile (ACN), cor-2CN, and cp-2CN along with a single water molecule are displayed in (Figure S1d-f). The population analysis based on Mulliken, natural and extended Hirshfeld Charge Model 5 (CM5).[8] Charges (Table S3) was carried applying the second-order Douglas-Kroll-Hess (DKH2) approach[9-11] in combination with the all-electron relativistic ANO-RCC-VDZP basis set.[12,13] All finite-size calculations were performed with the Gaussian09 program.[14] To obtain the optimized structures of a gold nanocluster adsorbed on the graphene substrate, periodic boundary condition (PBC) calculations were performed in combination with PBE-D2 functional[15] as implemented in the Vienna Ab-initio Simulation Package (VASP).[16,17] A minimum cutoff of 300 eV and 3 × 3 × 3 Γ-centered Monkhorst-Pack *k*-point mesh[18] per conventional 6 × 6 triclinic cell containing 72 carbon atoms and 20 gold atoms were used. In the first step, a unit cell containing a gold nanocluster of twenty atoms (Au_{20}) and 72 carbon atoms corresponding to the graphene substrate was fully optimized at the PBE-D2 level[15] using the VASP program.[16,17,19,20] A particular choice of the size and shape of the model nanocluster was based on the EXAFS data predicting the size of about 20 atoms and previous study by Li *et al.* arguing based on photoelectron spectroscopy and relativistic DFT calculations that Au_{20} has a tetrahedral geometry similar to a fragment of bulk face-centered cubic (fcc) gold.[21] The optimized structure was then used to construct a finite-size model (Figure S1c) which was further applied in calculations related to the binding of the gold nanocluster on the graphene substrate as well as

possible interactions between reactants (i.e., silane and ethanol) with the nanocluster (Table S7 and related comments).

3. Supplementary Tables

Table S1. Structural parameters resulting from fitting of the EXAFS signal of G(CN)-Au sample.

**Path**	**N**	**R (nm)**	**σ^2 (nm^2 x 10^{-5})**
Au-N	1	0.202(3)	4(1)
Au-O	1	0.195(2)	
Au-C	1	0.315(3)	
C-N	(Au-C)-(Au-N) if linearly coordinated	0.113(4)	

Table S2. Selected bond lengths (in Å), binding energies (BE, in kcal/mol), atomic spin densities (in a.u.) and Mulliken charges on the gold atom of model R-CN-Au systems in water computed at the SMD/PBE0/DEF2-TZVP level of theory.

Model	ACN-Au	Coronene-2CN-Au	cp-2CN-Au
**R_{C-N}/R_{N-Au}**			
R-CN-$Au^{(0)}$ (M=2)	1.149/ 2.196	1.154/ 2.252	1.154/2.247
R-CN-Au^{+} (M=1)	1.146/ 1.965	1.146/ 1.964	1.146/ 1.965
R-CN-Au^{3+} (M=1)	1.170/ 1.965	1.144/ 1.966	1.144/ 1.963
**Binding energy**			
R-CN-$Au^{(0)}$ (M=2)	-1.7	-1.2	-1.1
R-CN-Au^{+} (M=1)	-25.9	-24.2	-24.1
R-CN-Au^{3+} (M=1)	-69.7	-197.6	-206.0
**Spin density on Au**			
R-CN-$Au^{(0)}$ (M=2)	0.98	0.95	0.96
**Mulliken Charge density on Au**			
R-CN-$Au^{(0)}$ (M=2)	-0.13	-0.06	-0.07
R-CN-Au^{+} (M=1)	0.85	0.88	0.88
R-CN-Au^{3+} (M=1)	1.61	0.92	0.90

Comment: *The calculations revealed that the binding energy of Au(0) to G-CN is significantly smaller (-1.2 kcal/mol) than that of Au(I) (ca. -24 kcal/mol) suggesting that in the case of complete reduction of gold ions, the zero-valent atoms would easily be released from the nitrile sites and could group into nanoclusters.*

Table S3. Selected bond lengths (in Å) and partial atomic charges (Mulliken: q_{Mul}, natural: q_{nat}, and extended Hirshfeld Charge Model 5: q_{CM5}) on the gold atom of model R-CN-Au-H_2O systems in water computed at the all-electron relativistic DKH2-PBE0/ANO-RCC-VDZP/SMD(solvent=water) level of theory using the structures optimized at the PBE0/DEF2-TZVP(+ECP)/SMD(solvent=water) level.

**Model**	**ACN-Au-H_2O**	**cor-2CN-Au-H_2O**	**cp-2CN-Au-H_2O**
**R_{C-N}/R_{N-Au}/R_{Au-O}**			
R-CN-Au^+-H_2O (M=1)	1.145 / 1.945 / 2.066	1.146 / 1.943 / 2.061	1.145 / 1.943 / 2.061
R-CN-Au^{3+}-H_2O (M=1)	1.144 / 1.943 / 1.952	1.144 / 1.939 / 2.059	1.144 / 1.939 / 2.063
**Charge density on Au**	**q_{Mul} / q_{nat} / q_{CM5}**	**q_{Mul} / q_{nat} / q_{CM5}**	**q_{Mul} / q_{nat} / q_{CM5}**
R-CN-Au^+-H_2O (M=1)	0.49 / 0.68 / 0.50	0.51 / 0.69 / 0.52	0.51 / 0.69 / 0.52
R-CN-Au^{3+}-H_2O (M=1)	1.88 / 2.17 / 1.94	0.55 / 0.72 / 0.55	0.53 / 0.71 / 0.54

Table S4. Dehydrogenative coupling of dimethylphenylsilane (1a) and ethanol in the presence of various catalysts.[a]

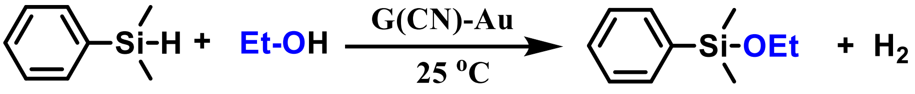

Entry	Catalysts	Conv.(%) / Sel.(%)	TOF (h^{-1})
1	-	n.d.	n.d.
2	G-CN	n.d.	n.d.
3[b]	$(Ph_3P)AuCl$	n.d	n.d.
4[c]	[(iPr)AuCl]	n.d.	n.d.
5	$HAuCl_4$	n.d.	n.d.
**6**	**G(CN)-Au**	**>99/99**	**96378**
7	G(CN)-Au-R	20/99	7895
8	GO	n.d.	n.d.
9	Au/GO	09/98	n.d..

[a]Reaction conditions unless specified otherwise: Dimethylphenylsilane (1 mmol), 10 mg of G(CN)-Au (0.6*wt*% based on Au) dispersed in 1.5 ml of ethanol, reaction for 5 min, 25 °C, Selectivity was determined upon the maximum conversion monitored by GC and GC-MS.
[b]5 mg of metal salt used, [c]10 mg of metal salt used, n.d. -Not detected.

Table S5. Oxidative coupling of organosilanes with alcohols; a comparison of performance.

Catalysts	Silane	Alcohols	Time (min/h)	TOF (h^{-1})	Atmo-sphere	Reference [a]
**G(CN)-Au**	Me_2PhSiH	MeOH	1 min	**139494 h^{-1}**	**air**	**This work [a, b]**
	Me_2PhSiH	EtOH	1 min	**96378 h^{-1}**		
	Me_2PhSiH	*n*-BuOH	5 min	**25000 h^{-1}**		
AgCr@CN-800	Me_2PhSiH	EtOH	3 min	327 h^{-1}	air	*ACS Sustainable Chem. Eng.*[22]
Co SAs/2D N–C	Me_2PhSiH	EtOH	2 hr	3858 h^{-1}	Ar	*Chem. Commun.*[23]
KCC-1-APTS/Au	Me_2PhSiH	EtOH	24 h	21625	air	*J. Mater. Chem. A*[24]
$Cu_3(BTC)_2$	Me_2PhSiH	*n*-BuOH	5 min	266	Ar	*Chem. Comm.*[25]
Cu(B)G	Me_2PhSiH	EtOH	24 h	4.16	air	*Angew. Chem., Int. Ed.*[26]
Au/HAPnano (667)	Me_2PhSiH	EtOH	1 h.	128	air	*Chem. Eur. J.*[27]
10Dod-AuNP	Me_2PhSiH	*n*-BuOH	1 h.	55000	air	*Adv. Mater.* [28]
[Au]-SMAP-Rh (75000)	Me_2PhSiH	EtOH	16 h	3750[c]	air	*Angew. Chem., Int. Ed.*[29]

[a]In batch mode. [b]In continuous mode. [c]TOF calculated from the TON, which is provided in the original article.

Reaction conditions for this work: Dimethylphenylsilane (1 mmol), 10 mg of G(CN)-Au (0.6 *wt*% based on Au) dispersed in 1.5 ml of ethanol, reaction for 5 min, 25 °C.

Table S6. Single-atom G(CN)-Au catalyzed oxidation of various organosilanes with water.[a]

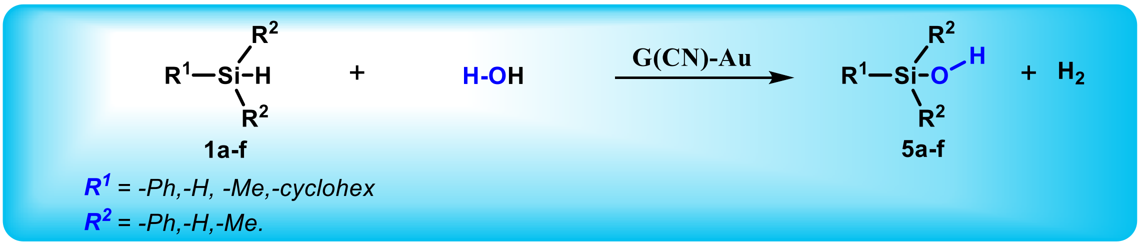

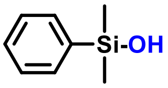

5a
C:99%, ***S***:99% (1h)

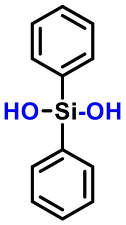

5b
C:99%, ***S***:80% (2h)

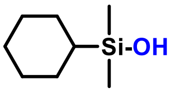

5c
C:99%, ***S***:99% (1h)

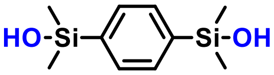

5d
C:99%, ***S***:90% (2h)

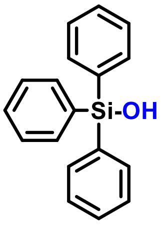

5e
C:99%, ***S***:90% (2h)

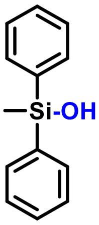

5f
C:50%, ***S***:99% (2h)

---

[a]**Reaction conditions:** 1 mmol substrate (silane),10 mg G(CN)-Au (0.6*wt* % based on Au) dispersed in 2 ml acetone and 0.1 ml water at 25 °C, percentage conversions (***C***) and selectivities (***S***) were determined and confirmed by GC and GC-MS. Selectivity was determined upon the maximum conversion monitored by GC and GC-MS.

Table S7. Binding energies (in kcal/mol), selected bond lengths (in Å), and Mulliken charges on the gold nanocluster of model Gr-Au_{20}…X systems (X = silane, ethanol) in water computed at the SMD/PBE0-D3/DEF2-TZVP level of theory. Corresponding structures are shown in Supplementary Fig. S29.

System	Binding energy	Bond lengths	Comments
**Gr…Au_{20}**		**R_{C-N}/R_{C-Au}**	**q(Au_{20})**
cp-$Au_{20}^{(0)}$ (M=1)	-53.1	2.96[a]	-0.58
cp-Au_{20}^{+} (M=2)	-56.2	2.96[a]	0.42
**(Gr-)Au_{20}…$HSiR_3$**		**R_{Au-H}/R_{H-Si}**	
$Au_{20}^{(0)}$…$HSiR_3$	-30.2	1.93/1.56	binding through H
Au_{20}^{+}…$HSiR_3$	-36.5	1.80/1.59	binding through H
cp-$Au_{20}^{(0)}$…$HSiR_3$	-17.3	1.88/1.56	binding through H
cp-Au_{20}^{+}…$HSiR_3$	-26.3	1.78/1.60	binding through H
**((Gr-Au_{20}…$HSiR_3$)…ethanol**		**R_{Au-H}/R_{Si-O}**	
($Au_{20}^{(0)}$…$HSiR_3$)…ethanol	-20.2	1.99/7.23	
(Au_{20}^{+}…$HSiR_3$)…ethanol	-15.2	1.79/6.13	large distance between silane and ethanol
(cp-$Au_{20}^{(0)}$…$HSiR_3$)…ethanol	-18.8	1.96/6.10	
(cp-Au_{20}^{+}…$HSiR_3$)…ethanol	-9.8	1.79/4.35	

[a]*The geometry of the neutral system optimized with PBC/PBE-D2.*

4. Supplementary Figures

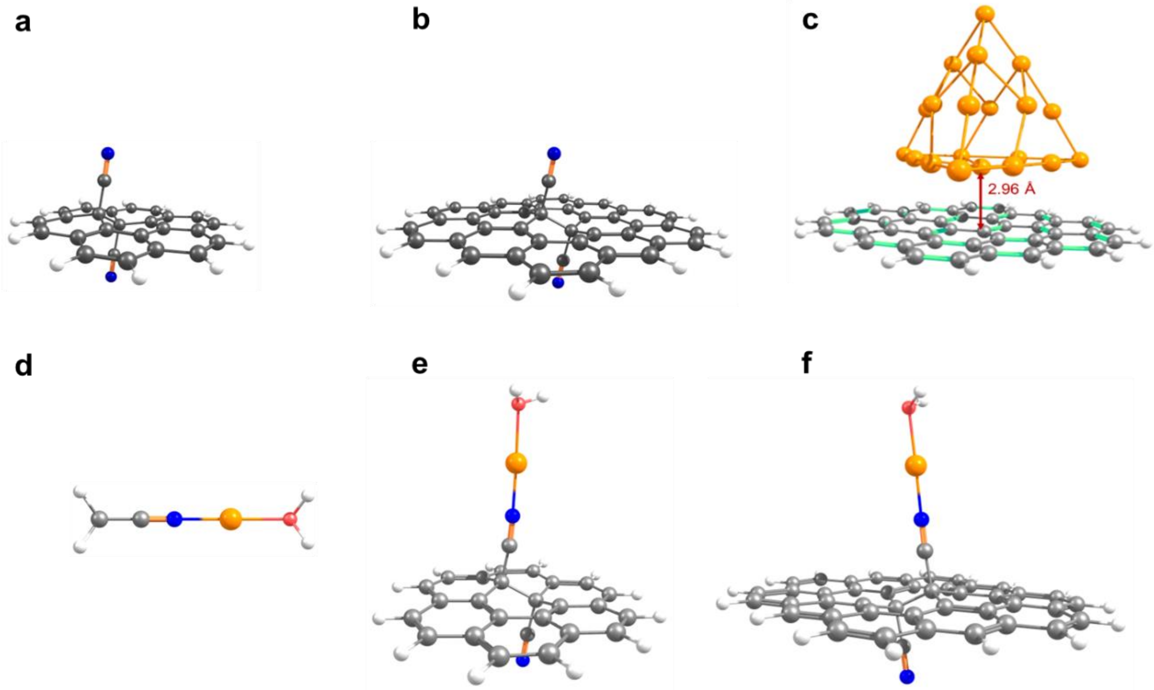

Figure S1. Model structures of (a) dicyano-coronene (cor-2CN), (b) dicyano-circumpyrene (cp-2CN), (c) Au20 nanocluster on the graphene substrate, (d) ACN-Au(I)-H_2O (ACN = acetonitrile), (e) cor-2CN-Au(I)-H_2O, and (f) cp-2CN-Au(I)-H_2O. Structures a, b, d-f were optimized at the PBE0/DEF2-TZVP/SMD level of theory. Structure c was obtained by a combined periodic and finite-size approach (see text for details). Carbon atoms are grey, nitrogen blue, hydrogen white, and gold orange.

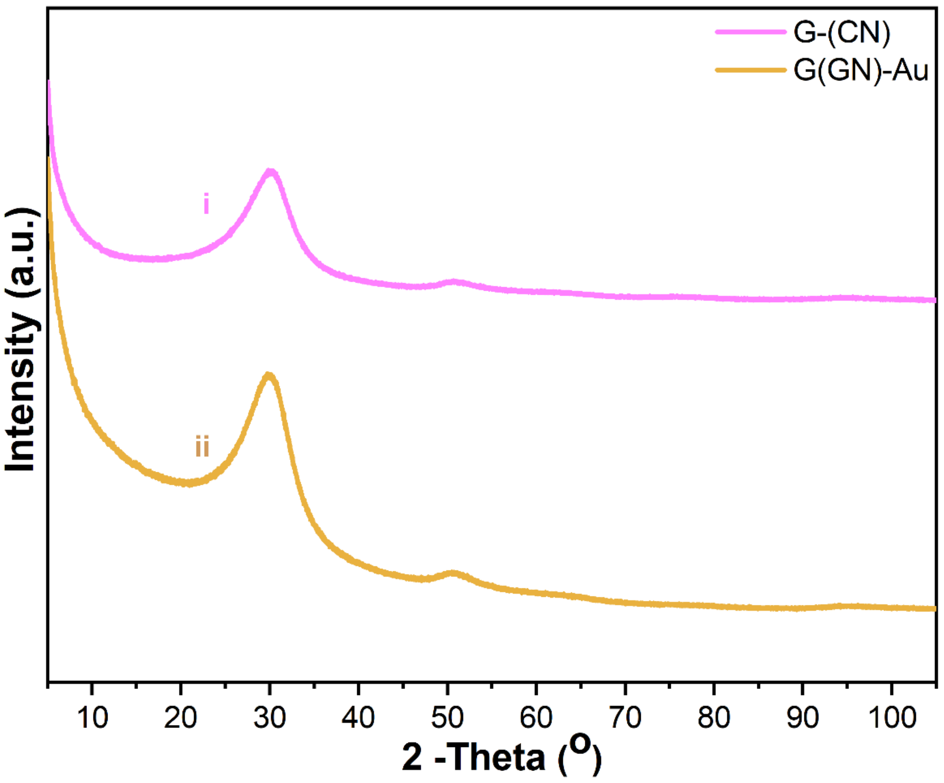

Figure S2. X-ray diffraction pattern of G(CN)-Au. i) pristine cyanographene (G-CN) and ii) the gold-loaded cyanographene (G(CN)-Au). X-Ray radiationused: Co K-alpha (1.789 Å).

Comment*: The catalyst (as well as the rest of the solids) lacks completely reflections from any inorganic nanoparticles. Only the presence of very broad reflection at 30.4 degrees was observed in G-CN and G(CN)-Au, typical of non-restacked graphene powders present in carbon materials.*

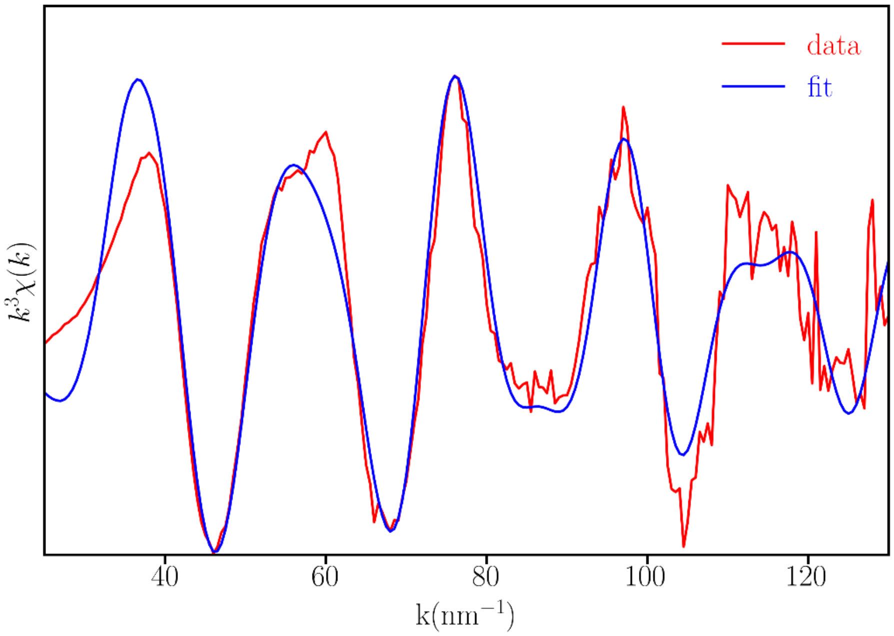

Figure S3. Fitting of k²-weighted EXAFS signal for the G(CN)-Au sample.

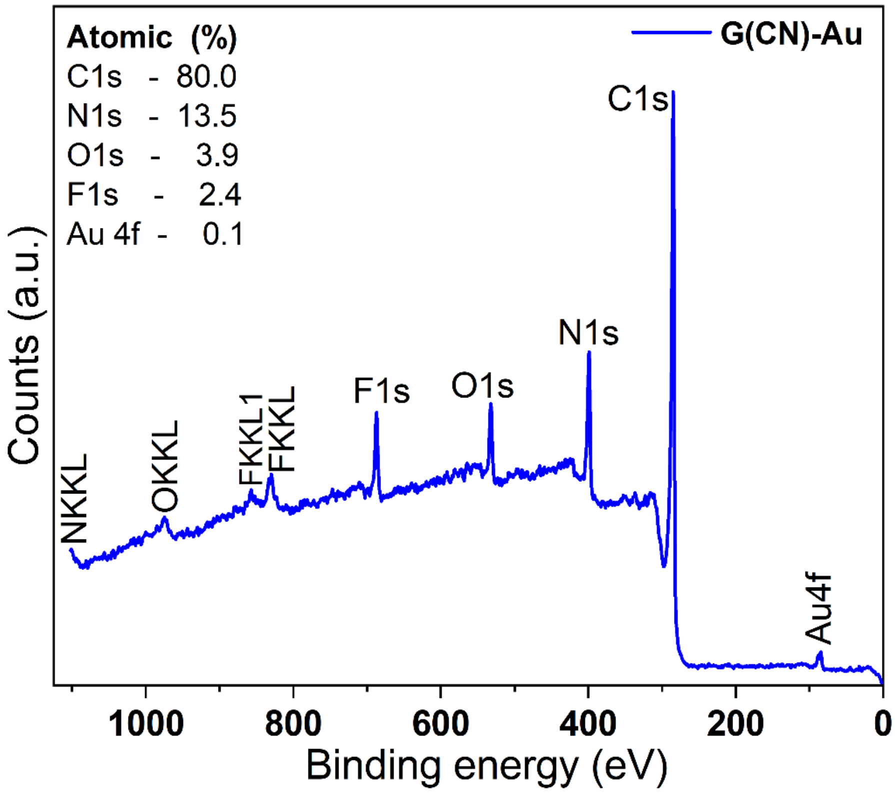

Figure S4. Survey XPS spectrum for G(CN)-Au

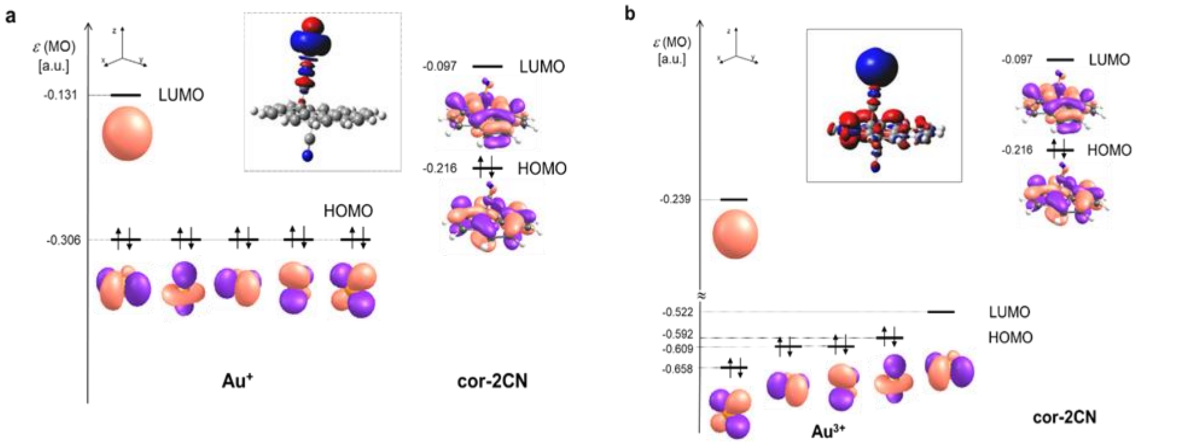

Figure S5. Frontier molecular orbitals (contour isovalue 0.08) of Au(I/III) cations and cor-CN with EDD plots (shown as insets; contour isovalue 0.003 e) of a) G(CN)-Au(I) and b) G(CN)-Au(III) complexes in water obtained at the PBE0/DEF2-TZVP/SMD level of theory.

Comment: *Whereas the energy of the LUMO in* Au(I) *lies higher compared to the HOMO of cor-2CN, for* Au(III) *the ordering is reversed. Consequently, the* Au(III) *cation can be readily reduced by the cor-2CN type of substrates.*

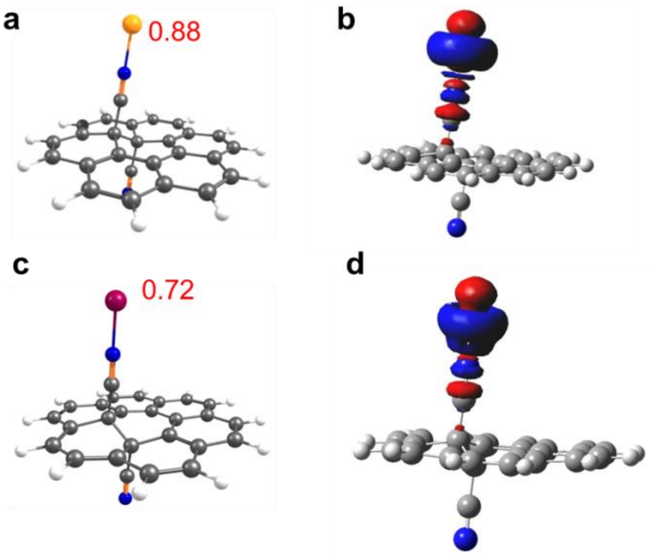

Figure S6. Comparison of the binding in a, b) cor-2CN… Au(I) (PBE0/DEF2-VTZP) and c, d) cor-2CN-Cu (I). (B3LYP/6-31+G(d)) (contour isovalue in EDD plots: 0.003 a.u.) in water. Similarities of Mulliken charges on metal ions as well as electron density changes accompanying the formation of G(CN)…metal bonds involving polarization of the CN bond and the increase of the electron density on the metal ion in the plane perpendicular to the G(CN)…metal σ bond suggest that the binding in cor-2CN… Au(I) is analogous to that in cor-2CN...Cu(I). Carbon atoms are grey, nitrogen blue, hydrogen white, gold yellow, and Cu purple.

Discussion for the HR-XPS of N1s envelope

The N1s spectra of G-CN and G(CN)-Au were deconvoluted into three components (Figure S7a). The two main components (N_1 and N_2) in the spectra could be accounted to the attachment of nitrile (-CN) groups in different local environments (i.e., environments with and without nearby defects in the graphene skeleton). The relative areas under the curve of the two main components showed some change, where the N_1/N_2 ratio altered from 1.5 to 1.9 after the Au(I) ions were bound onto the material. Particularly, the lower binding energy N_1 component was enriched after coordination of the Au(I) ions, in line with the theoretical calculations of the N1s binding energies for nitrile nitrogens in G-CN (399.3 eV)[30] and G(CN)-Au (398.8 eV, this work). Intuitively, the Au(I) cation should attract electron density from its ligands. For instance, both experimental and computational studies have shown that the binding energies of nitrogen atoms in N-doped graphene shift to higher values upon the complexation with Co(II) ions.[31]

The increased area of the lower binding energy component after coordination was thus a clear indication of charge-transfer toward the -C≡N groups in this case. The shift in the binding energy also confirms the interactions (i.e. the formation of coordinative bonds) between the nitrile groups and Au cations.

The two main components in the N1s XPS spectra can be ascribed to the attachment of nitrile groups in different local environments (i.e. environments with and without nearby defects in the graphene skeleton). This is also clearly reflected in the IR band of the nitrile groups, which is asymmetric and was fitted with three C≡N components (Figure S7b).

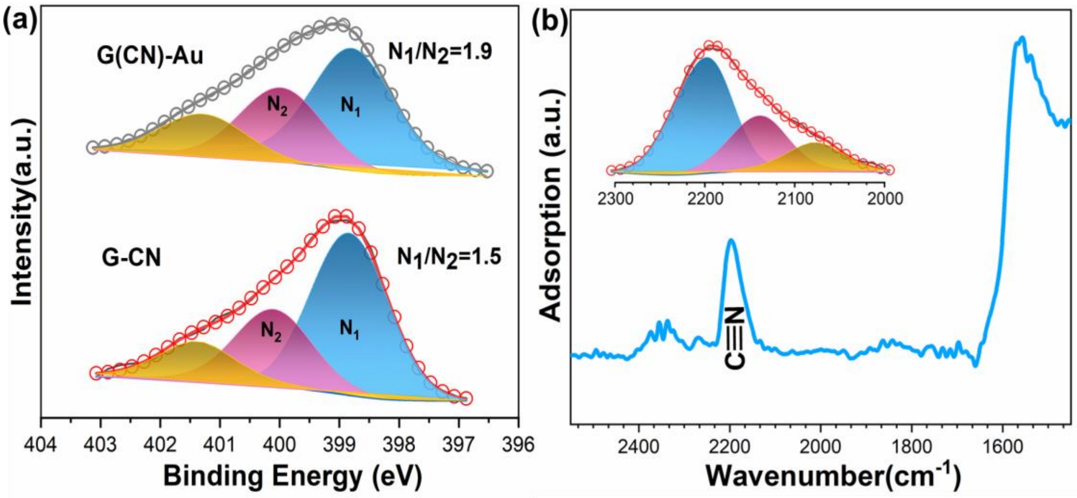

Figure S7. N1s XPS spectrum and FT-IR spectrum of G(CN)-Au. a) Deconvoluted HR-XPS N1s spectra for G-CN and G(CN)-Au samples. Circles represent experimental data and solid lines the fitting results. b) IR spectrum of G-CN showing the asymmetric nitrile band at 2200 cm^{-1}. The inset shows this asymmetric band after deconvolution into three components, supporting the hypothesis that the material contains nitrile groups in distinct local environments.

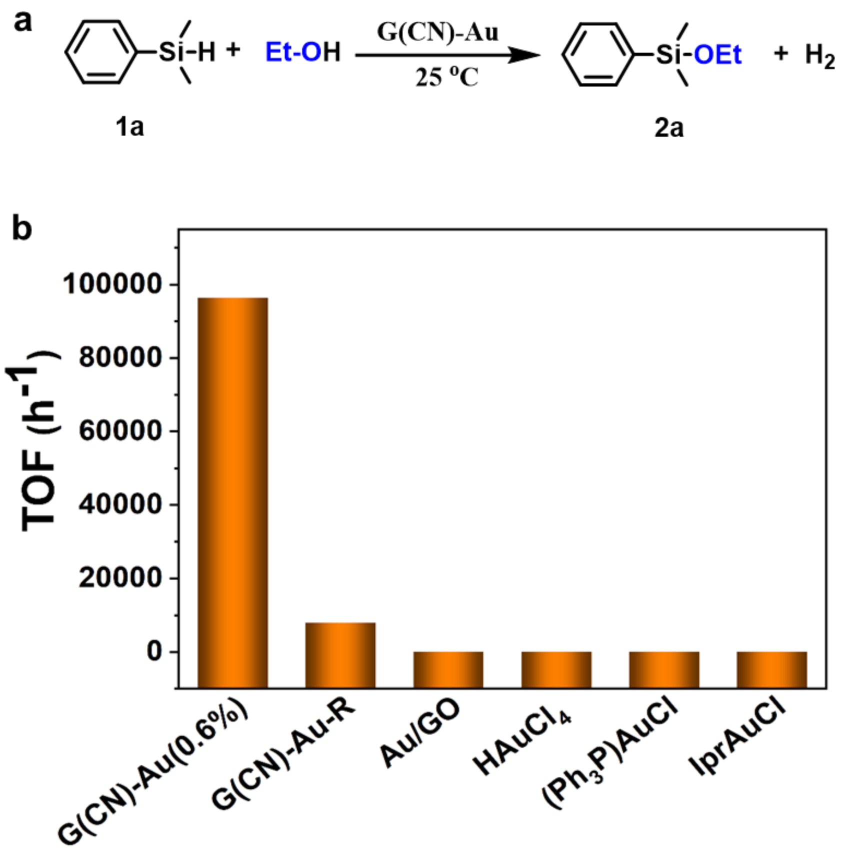

Figure S8. Dehydrogenative oxidation of silanes. a) Dehydrogenative coupling of silanes with ethanol. b) Comparison of TOF values among different commercial Au catalysts, G(CN)-Au and G(CN)-Au-R (reduced catalyst).

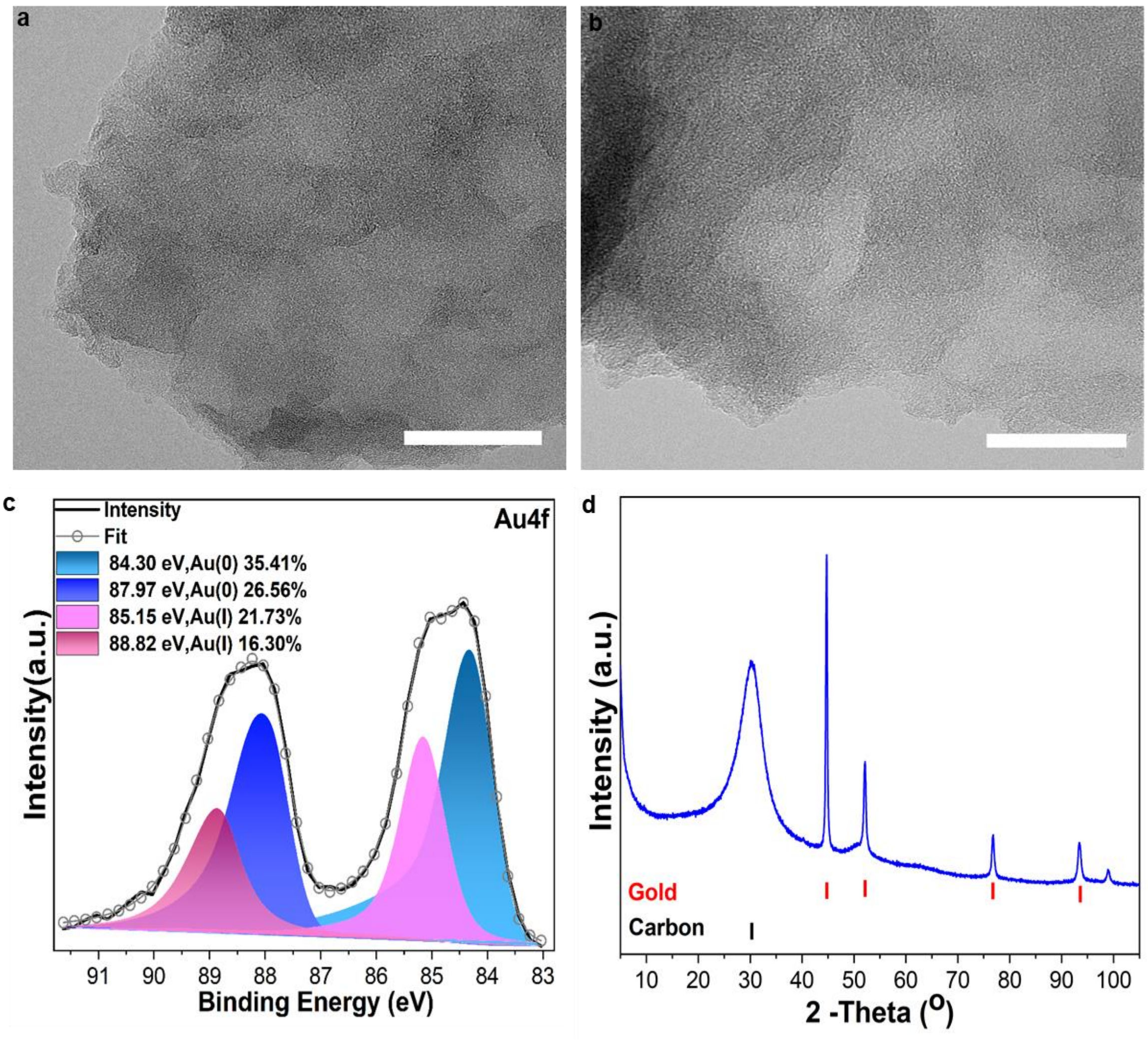

Figure S9. a, b) TEM images of G(CN)-Au-R, (scale bar 50 nm and 20 nm). c) HR-XPS shows the presence of Au (0) and Au(I). d) The X-ray powder diffraction (XRD) spectrum displays the presence of very broad reflection at 30.4 degrees, typical for non-restacked graphene powders and present in carbon materials (PDF card 00-041-1487) and charescristic peaks of gold (PDF card 04-003-3089).

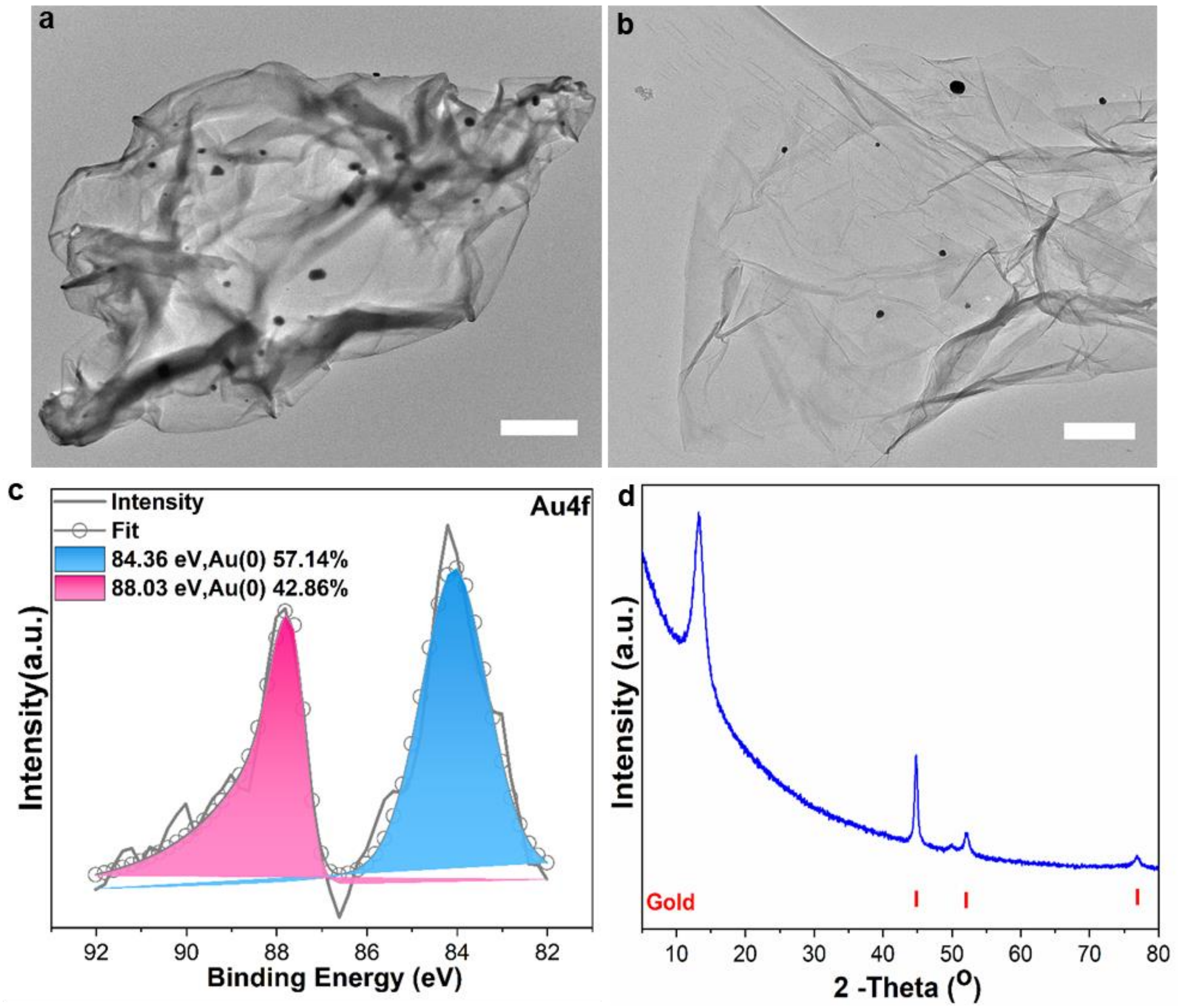

Figure S10. a, b) TEM images of Au/GO, (scale bar 1μm and 500 nm) displayed the small nanoparticles with size (40-60 nm). c) HR-XPS shows the presence of Au (0) species. d) X-ray diffraction demonstrates the peak at 44.71° (space group Fm-3m JCPDS card number 01-071-4615) corresponding to gold nanoparticles.[32]

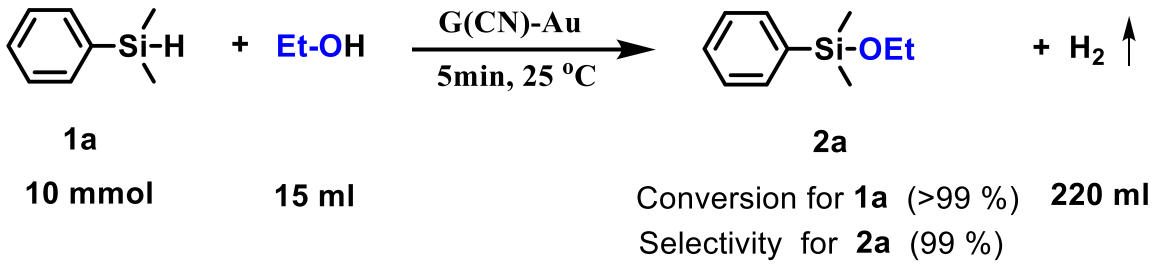

Figure S11. Gram-scale dehydrogenative coupling of dimethylphenylsilane with ethyl alcohol.

Characterisation of recycled catalysts

The valence state of recycled catalyst, as analysed by XPS, retained up to 21 % Au(I), though four peaks with binding energies of 83.95, 87.62, 84.99, 86.66 eV corresponding to Au(0) $4f_{7/2}$, Au(0) $4f_{5/2}$, Au(I) $4f_{7/2}$, and Au(I) $4f_{5/2}$, respectively were observed[33] (Figure. S12b) revealing that Au(0) species are generated in recycled catalyst. Post-recycling TEM of G(CN)-Au after three recycling experiments revealed the formation of small Au(0) nanoparticles probably due to the reduction of active Au(I) sites or cationic species by the silane substrates[34] during the reaction (Figure. S11c-g).

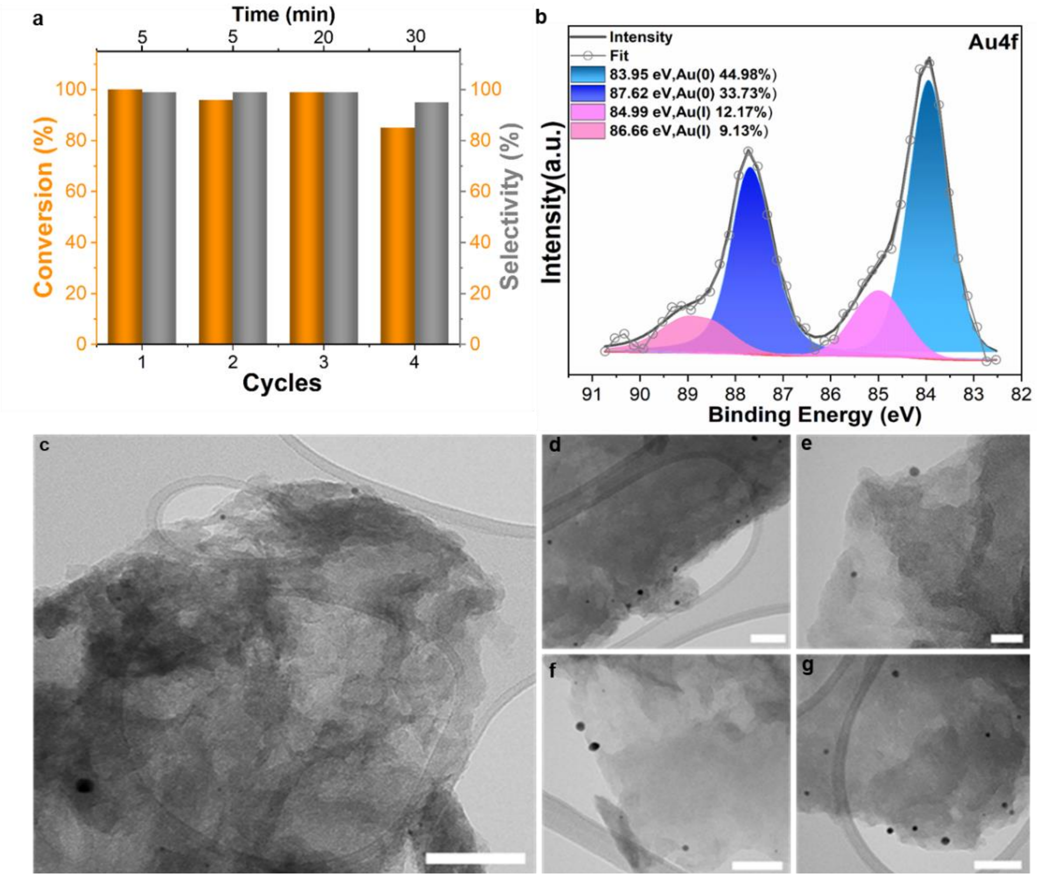

Figure S12. Characterizations of recycled G(CN)-Au.

a) Catalytic recyclability of G(CN)-Au catalyst for dehydrogenative coupling of dimethylphenylsilane with ethanol. **Reaction conditions:** Dimethylphenylsilane (1 mmol), 10 mg of G(CN)-Au (0.6wt% based on Au) dispersed in 2 mL of ethanol, reaction for 5 min, 23 °C. b) HR-XPS shows the presence of Au(I). c-g) c), scale bar 100 nm, d, e) scale bar 20 nm and f, g) scale bar 50 nm.

Stability of G(CN)-Au catalyst under continuous flow

Dimethylphenylsilane conversion (90-99%) and the product selectivity (70-96% to dimethylethoxyphenylsilane) was maintained after streaming for 5 to 25 hours affirming the stability of G(CN)-Au catalyst under continuous flow (Figure S13).

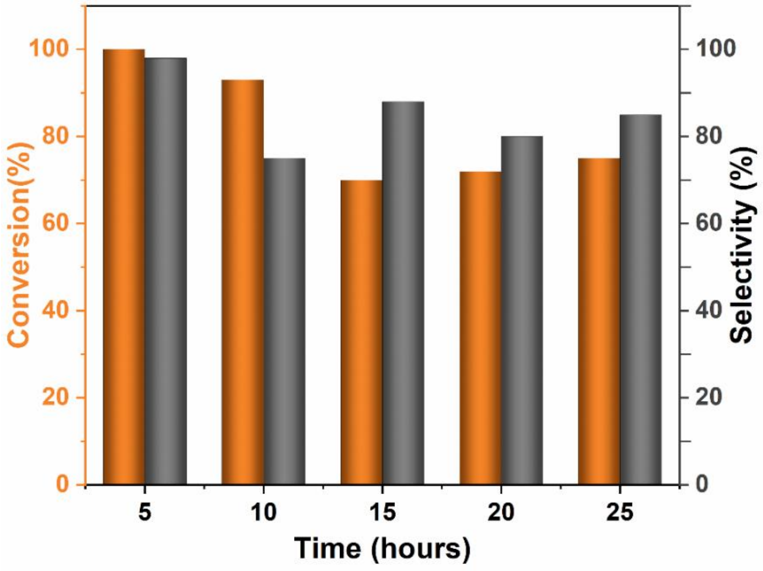

Figure S13. Stability performance of G(CN)-Au for the conversion of dimethylphenylsilane under continuous flow in function of time-on-stream.

Insights from continuous wave (CW) EPR spectroscopy measurements

The X band EPR resonance envelopes of the solid $HAuCl_4$ metal precursor (Figure. 14A(a)) neat G-CN compound (Figure S14A(b)), and the G(CN)-Au active catalyst (Figure S14A(c)), were recorded at T = 143 K. Screen shots of the EPR resonance spectra obtained, as recorded, from the EPR spectrophotometer are given in Figure S15-S17. The Au(III) chloride salt used for the synthesis of the active catalyst does not show any strong resonance signal (Figure S14A(a)), due to the diamagnetic nature of the Au(III) electronic configuration ([Xe]$4f^{14}5d^{8}$). Only a very weak resonance emerges upon closer inspection of the EPR spectrum, around g = *2.00* (Figure S14A), upper red-spectrum. This resonance is expected to arise from small paramagnetic Au(0) nanoclusters present/entrapped within the solid gold precursor, $HAuCl_4$.[35] The EPR signal of neat G-CN is depicted in Figure S14A(b), that exhibits a strong resonance signature centered at g value of 1.9960, arising from the presence of spin containing defects embedded in the organic G-CN support. Furthermore, the signal cannot be simply described by a single Lorentzian or Gaussian signal and shows broad resonance tails developing slightly asymmetrically with respect to the central (and narrow) $d\chi^{II}/dB$ resonance line. Such feature indicates the existence of spin defects with different nature embedded in the G-CN backbone, some of which solely localized on C centers and some place nearby the CN groups, hence containing effective nuclear hyperfine terms (A) originating from the ^{14}N nuclei (I_N = 1) that perturbs their electronic transitions ($H = g\,\mu_B\,B_0\,S + \Sigma\,A{\cdot}S{\cdot}I$). The spin density of neat G-CN, evaluated against $CuSO_4$ standard, accounts for 2.0×10^{19} spin/g. The EPR signal of the active catalyst G(CN)-Au is shown in Figure S14A(c), it displays similar features as those seen in G-CN Figure S14A(b), similarly to G-CN with a strong derivative signal at g = 1.9960. However, the recorded EPR signature of G(CN)-Au is also characterized by slightly broader wings compared to the G-CN resonance. The recorded G(CN)-Au EPR spectrum indicates that the gold cations adopt either the diamagnetic Au(III) or the diamagnetic Au(I) oxidation states. Although the minor presence of Au(0) cannot be totally excluded here, as we noticed the

presence of Au(0) in the starting salt material, but its contribution (and concentration) to the observed EPR spectrum in G(CN)-Au Figure S14A(c) is negligible. This appears in agreement with the XPS results, in which Au(0) was not observed. The spin density of G(CN)-Au, evaluated against $CuSO_4$ standard, falls to 1.57×10^{19} spin/g. Thus, a substantial reduction (~21%) of the spin concentration is observed in the active catalyst compared to neat G-CN. The result can be interpreted in terms of an electron transfer occurring from the G-CN support to the Au(III) cations during the synthesis of G(CN)-Au, forming Au(I) centers ([Xe]$4f^{14}5d^{10}$) entrapped/coordinated to the CN groups of the organic matrix. In addition, the possible generation of diamagnetic Au(I)O^- specie from Au(0) cannot be excluded.[36]

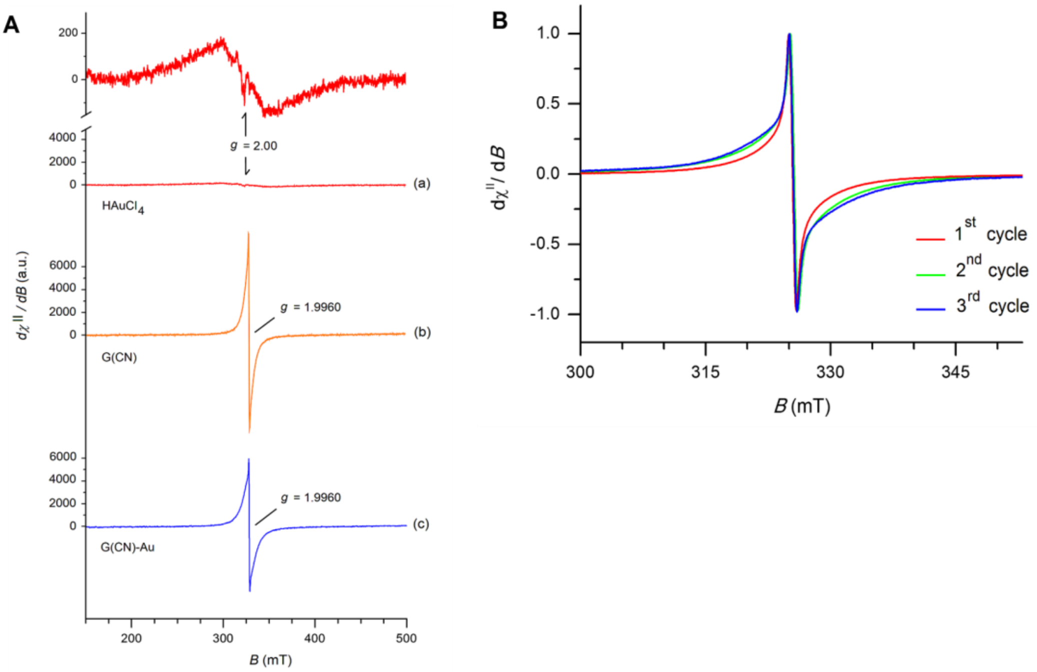

Figure S14. X-band EPR spectra of powder materials recorded in a polypropylene VSM holders. Panel A. a) $HAuCl_4$ salt precursor, b) neat G-CN and c) G(CN)-Au. The VSM holder background signal has been subtracted from the resonance traces shown in a) to c). Experimental parameters: 9.14 GHz, T = 143 K, 100 kHz modulation frequency, microwave power of 0.8 mW (a), 0.6 mW (b), and 0.6 mW (c). Sweep time of 4 min and time constant (t) of 0.03 sec. Sweep time of 4 min and time constant (t) of 0.03 sec. **Panel B.** X-band EPR spectra (9.09 GHz, T = 77 K) of recovered powder materials of G(CN)-Au, after one, two and three reaction cycles. Experimental parameters: 100 kHz modulation frequency, 0.8 mT modulation width, microwave power of 2.0 mW, sweep time of 2 min and time constant (t) of 0.03 sec.

These observations from the EPR analysis appears in harmony with the XPS results reported earlier, which indicated two different types of embedded Au(I) cations in G-CN, some being coordinated to the CN groups and the rest electrostatically entrapped as $Au(I)O^-$ specie by positively charged vacancies in the G-CN basal plane. It should be noted that only the former Au(I) site may function as catalytically active center in the H-abstraction process from silyl substrates, because only such sites can facilitate the $Au(I)\ldots H^-$ interaction (see the Step 3 in (Figure. 3, main text) within turnover, while interactions between $Au(I)O^-$ and H^- should be hampered by electrostatic repulsions.

We also assessed the extent to which EPR features of G(CN)-Au catalyst changed after one, two and three reaction cycles, upon collecting and drying the material from the reaction mixture (Figure S14B). The as recorded EPR spectra (screen shots) taken from the EPR instrument are given in Supplementary Figure S18-S20. From the sequential catalyst's recycling, increase in the EPR signal associated to the broad wings underneath the strong $g = 1.9960$ signature. Taken into consideration the EPR features shown in (Figure S14A(a)), in which the presence of small Au(0) nanoclusters in $HAuCl_4$ salt gives weak and broad EPR resonance at $\sim g = 2.00$, we suggest that in G(CN)-Au the witnessed increase, upon recycling, of the broad wings is associated to a slow accumulation of small Au(0) clusters, which do not contribute to catalysis. For further reference, a model system made of GO and gold salt was assembled, in the same manner as G(CN)-Au wherein Au/GO composite displayed EPR features associated with the formation of large Au(0) nanoclusters entrapped in the organic framework (Figure S21-S22). Clearly, the Au(0) cluster being formed in G(CN)-Au after some reaction cycles are much smaller (in dimension) than those observed in the model system Au/GO, and should comprise only few Au atoms interacting together. These results are consistent with the observed mild decrease in the catalytic efficiency of G(CN)-Au after sequential re-use, and with the XPS results obtained from the recovered catalysts.

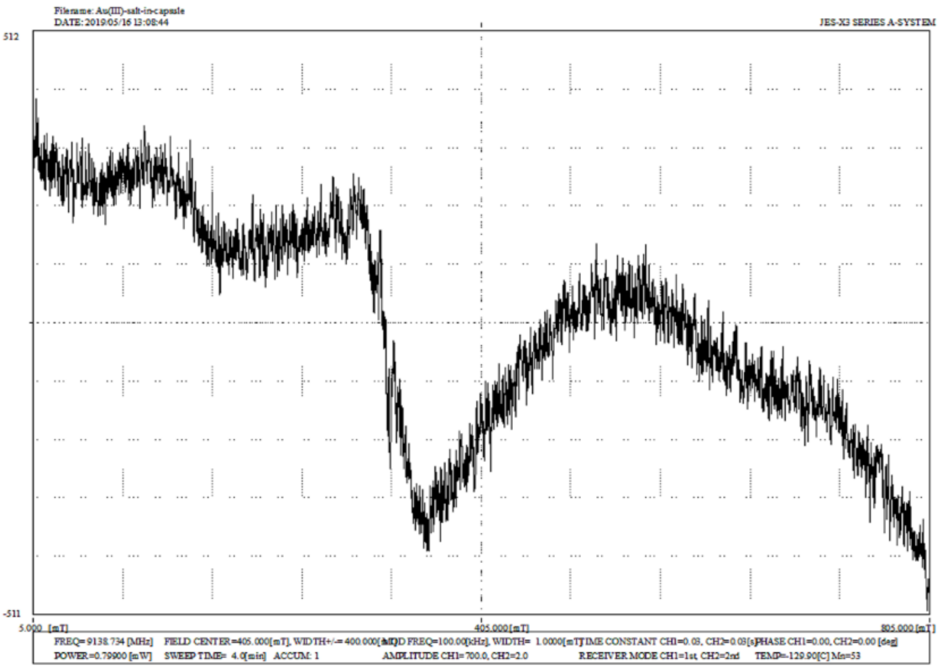

Figure S15. X-band EPR spectrum of $HAuCl_4$ precursor recorded at T = 143 K. Details of the experimental settings used within signal acquisition are given in the spectrum plot, which is reported as recorded.

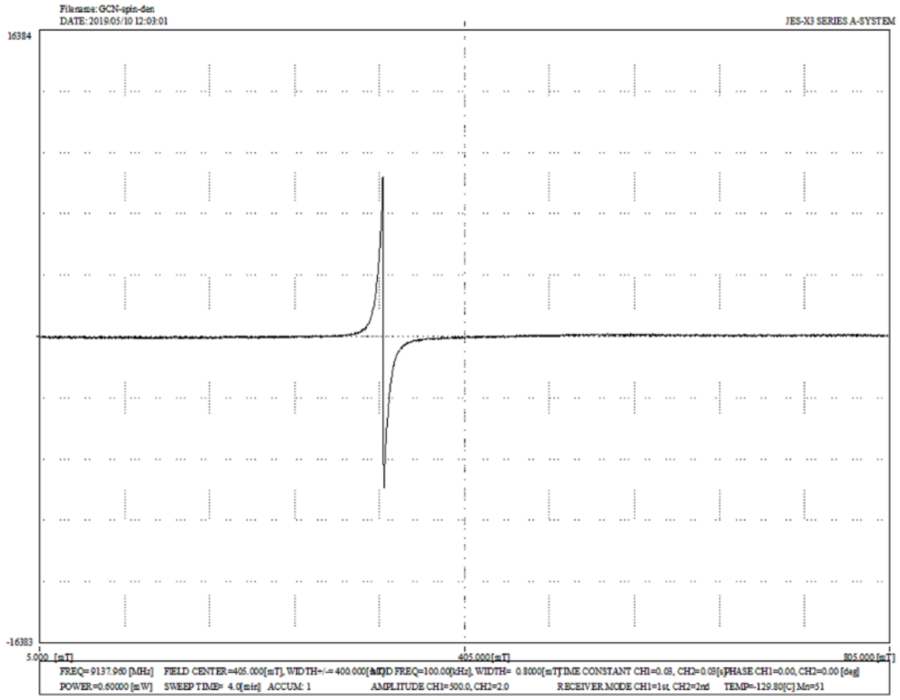

Figure S16. X-band EPR spectrum of neat G-CN recorded at T = 143 K. Details of the experimental settings used within signal acquisition are given in the spectrum plot, which is reported as recorded.

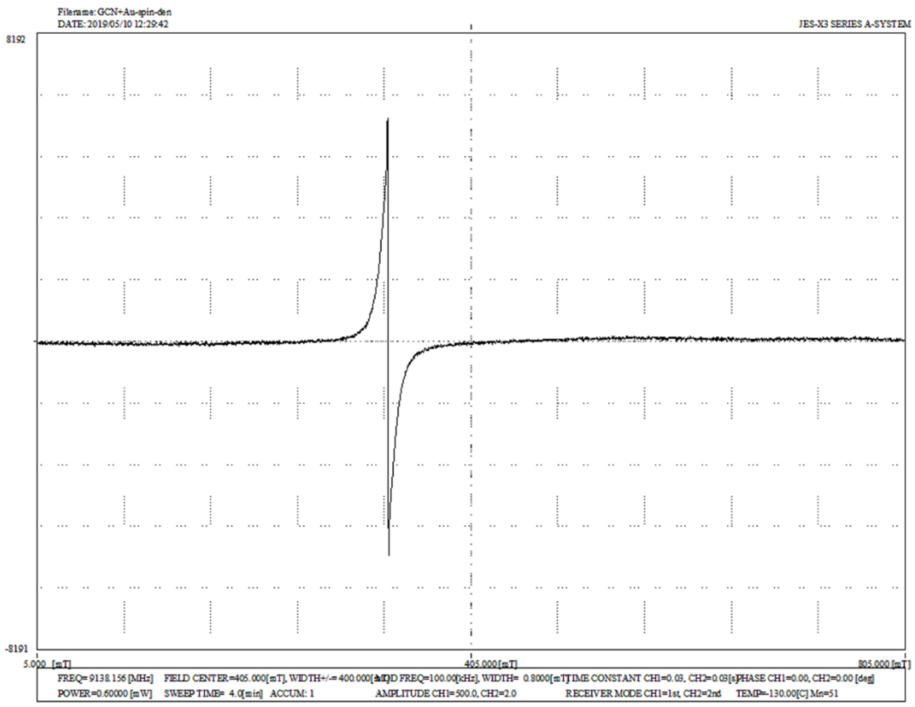

Figure S17. X-band EPR spectrum of G(CN)-Au precursor recorded at T = 143 K. Details of the experimental settings used within signal acquisition are given in the spectrum plot, which is reported as recorded.

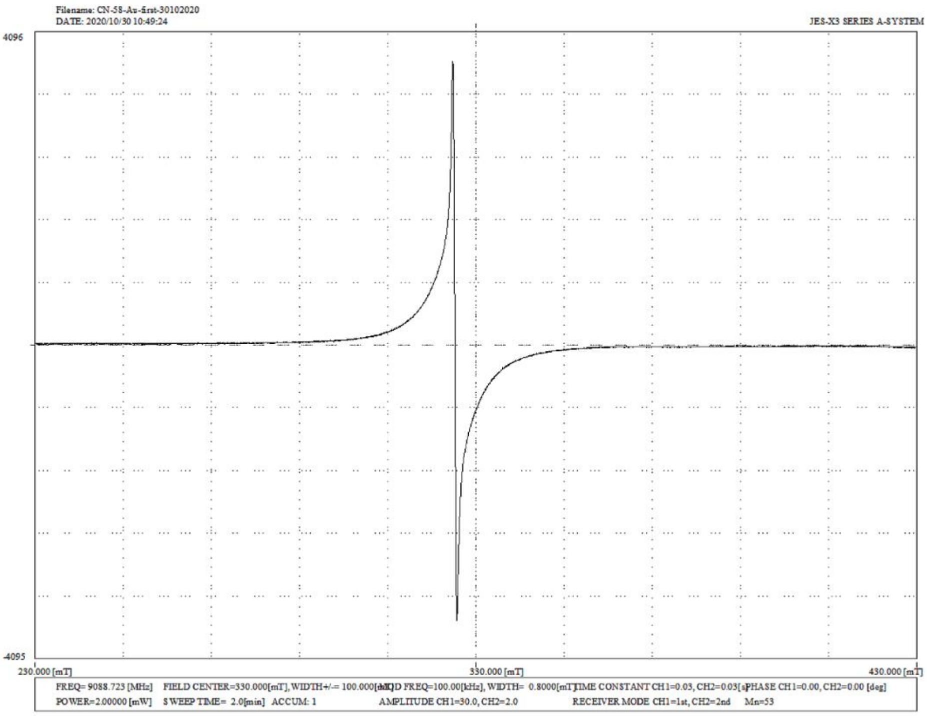

Figure S18. X-band EPR spectrum of G(CN)-Au precursor recorded at T = 77 K as recovered powder material after the first catalytic cycle. Details of the experimental settings used within signal acquisition are given in the spectrum plot, which is reported as recorded. The EPR signal intensity has not been divided by the mg of material placed in the EPR tube.

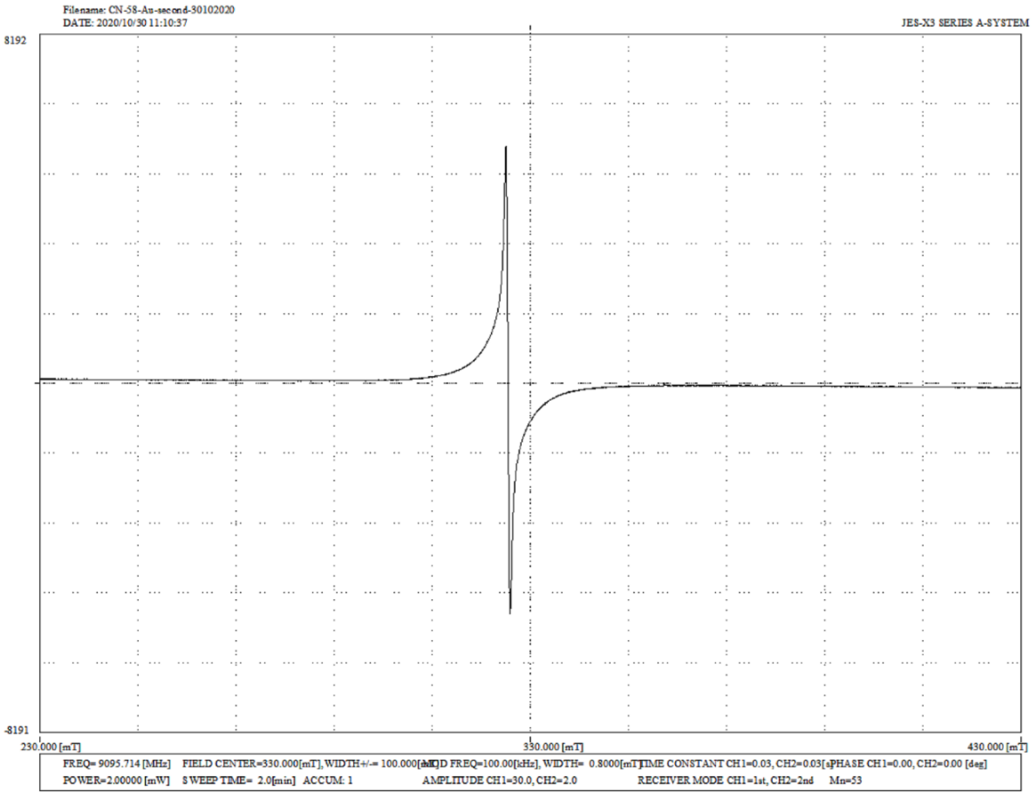

Figure S19. X-band EPR spectrum of G(CN)-Au precursor recorded at T = 77 K as recovered powder material after the second catalytic cycle. Details of the experimental settings used within signal acquisition are given in the spectrum plot, which is reported as recorded. The EPR signal intensity has not been divided by the mg of material placed in the EPR tube.

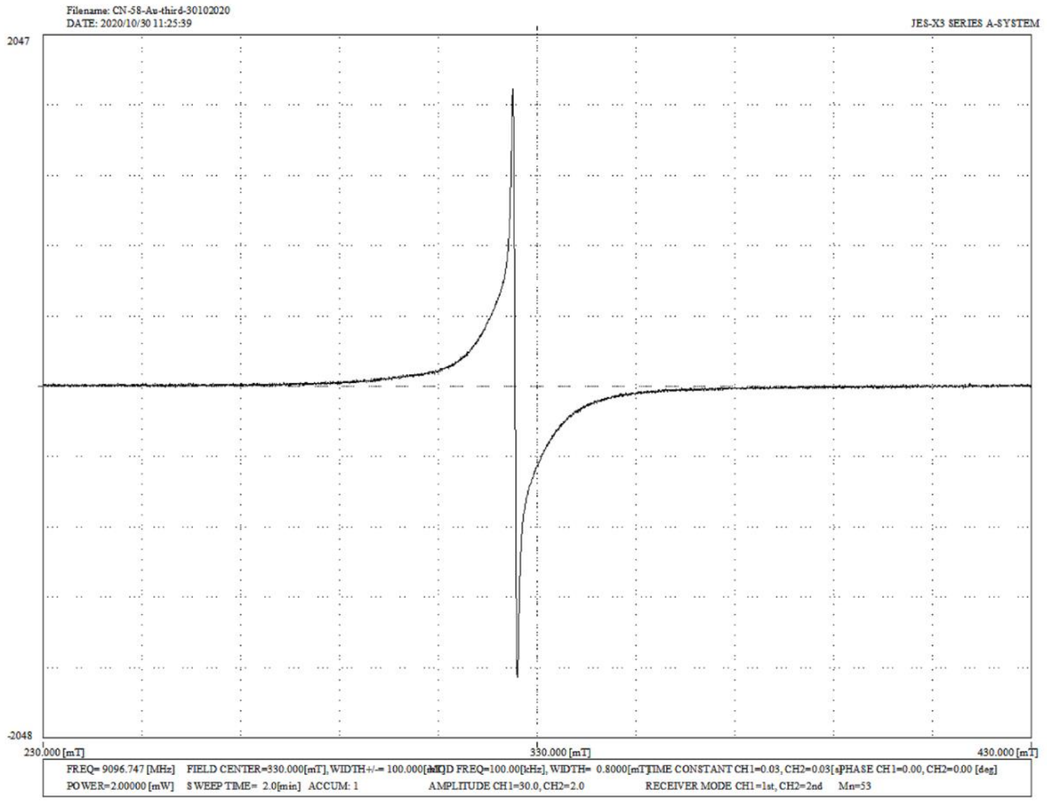

Figure S20. X-band EPR spectrum of G(CN)-Au precursor recorded at $T = 77$ K as recovered powder material after the third catalytic cycle. Details of the experimental settings used within signal acquisition are given in the spectrum plot, which is reported as recorded. The EPR signal intensity has not been divided by the mg of material placed in the EPR tube.

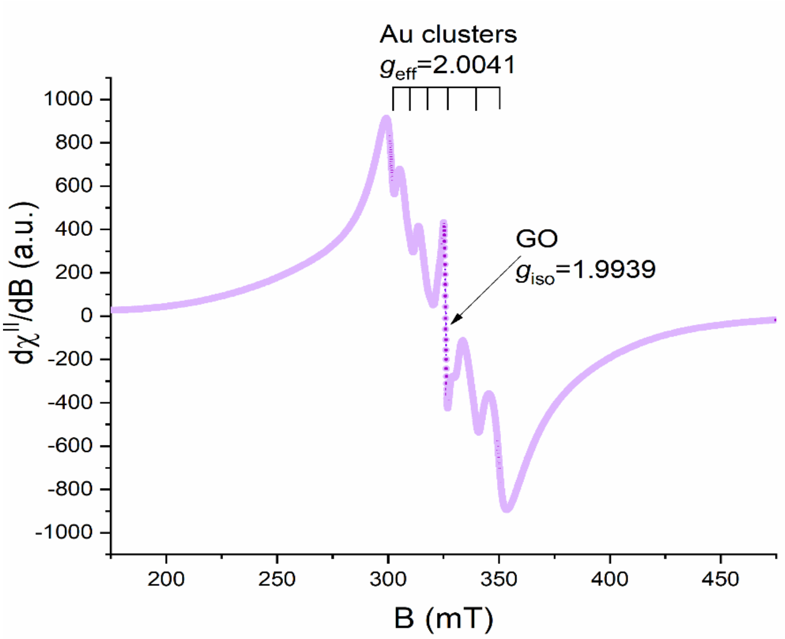

Figure S21. X-band EPR spectra (9.09 GHz, T = 77 K) of graphene oxide (GO) with gold nanoclusters. Experimental parameters: 100 kHz modulation frequency, 0.7 mT modulation width, applied microwave power of 1.0 mW, sweep time of 2 min. with 3 signals accumulations and time constant (t) of 0.03 s. The screen-shot of this measurement taken from the EPR spectrophotometer is given in Figure S22.

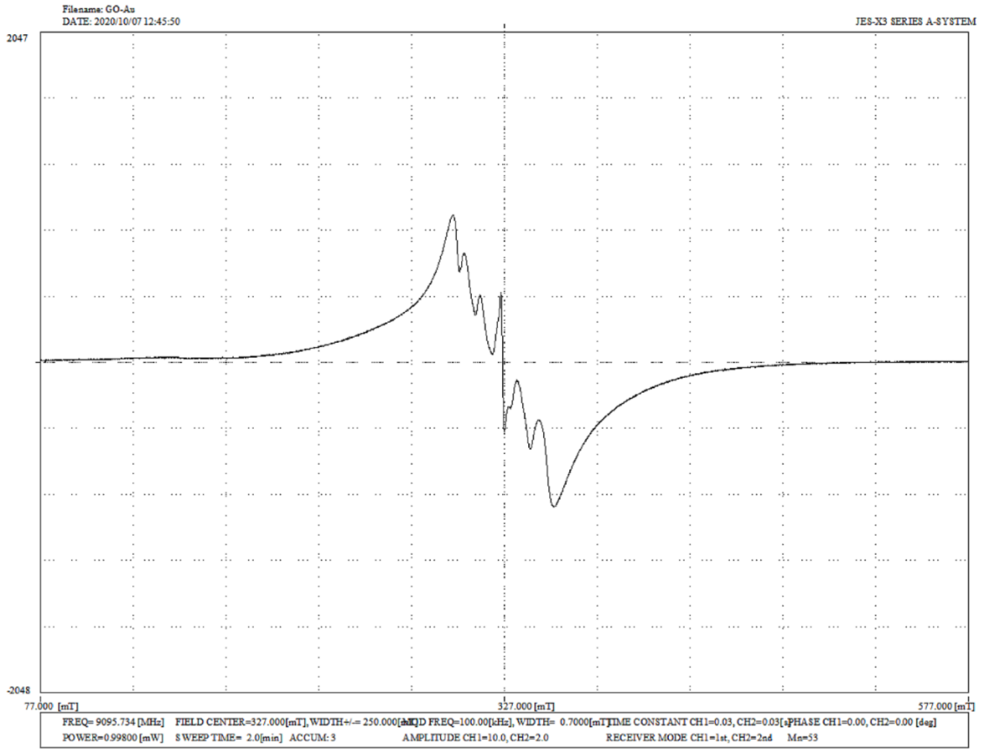

Figure S22. X-band EPR spectrum of GO with Au nanoclusters. Details of the experimental settings used within signal acquisition are given in the spectrum plot, which is reported as recorded.

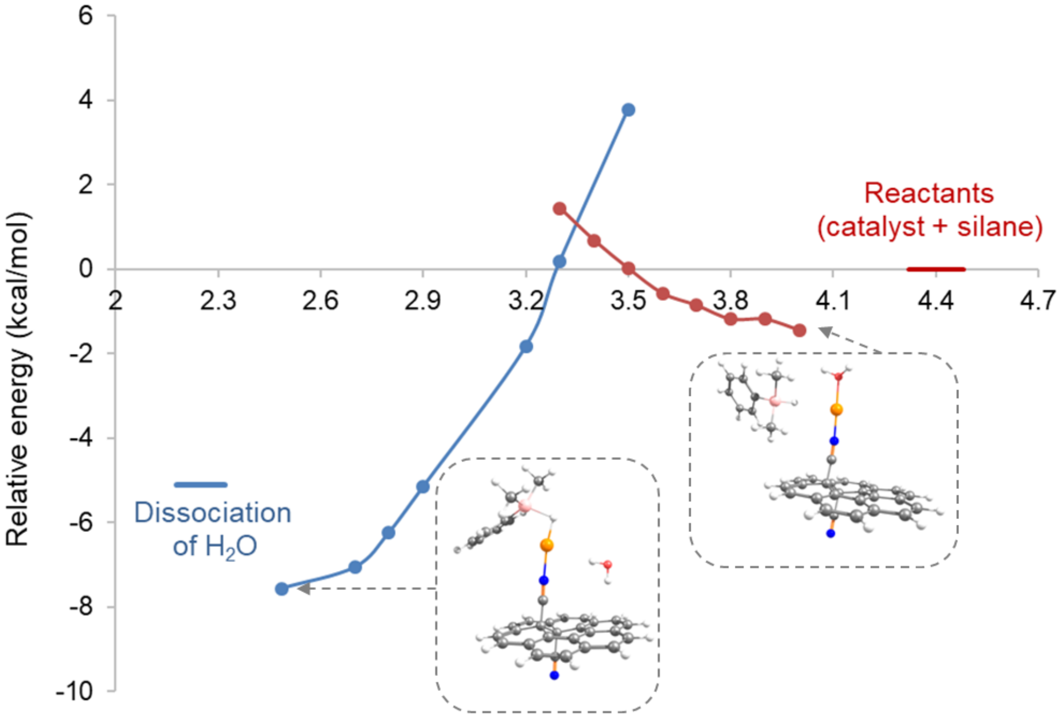

Figure S23. An attack of a silane molecule on the GCN-Au(I)-H_2O site in water (step 2 in Scheme 1). Reaction energies (in kcal/mol) related to the energy of separated reactants were obtained at the PBE0/Def2TZVP/SMD level of theory. Carbon atoms are grey, nitrogen blue, hydrogen white, gold orange, and silicon yellow.

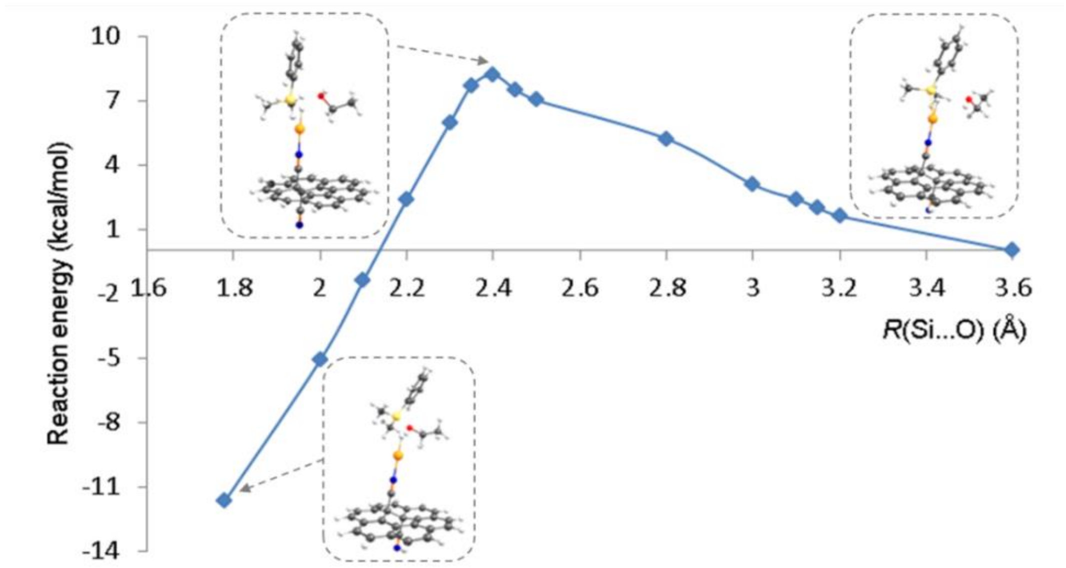

Figure S24. A direct attack of an ethanol molecule on the silane ligand in water (step 3 in Fig. 3). Reaction energies (in kcal/mol) were obtained at the PBE0/Def2TZVP/SMD level of theory. Carbon atoms are grey, nitrogen blue, hydrogen white, oxygen red, gold orange, and silicon yellow.

Comment: *The alcohol can alternatively bind to the Au(I) site with the BE of -3.4 kcal/mol (practically with no barrier; see (Figure S24), adopting a square planar configuration with the ethanol molecule in the trans position with respect to the silane moiety (Figure S25). The barrier to bind to silane from this configuration is 12.0 kcal/mol (Figure S26).*

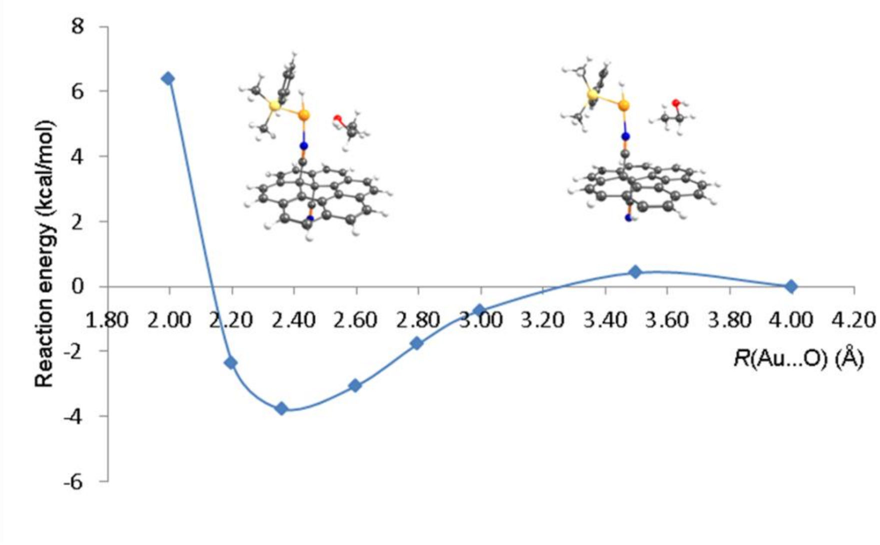

Figure S25. Binding of an ethanol molecule with G(CN)-Au(I)...DMPS (attack on the Au(I) site) in water leading to a square planar complex. Reaction energies (in kcal/mol) were obtained at the PBE0/Def2TZVP/SMD level of theory. Carbon atoms are grey, nitrogen blue, hydrogen white, oxygen red, gold orange, and silicon yellow.

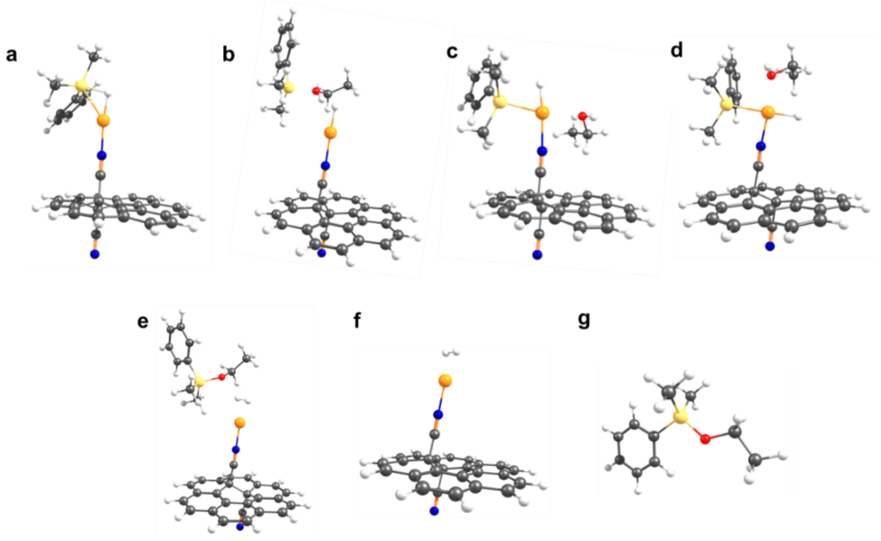

Figure S26. Optimized structures of (a) G(CN)-Au(I)...DMPS, (b-d) G(CN)-Au(I)...DMPS…ethanol, (e) G(CN)-Au(I)...H_2…product, (f) G(CN)-Au(I)...H_2 and (g) the product in water obtained at the PBE0/DEF2-TZVP/SMD level of theory. Carbon atoms are grey, nitrogen blue, hydrogen white, oxygen red, gold orange, and silicon yellow.

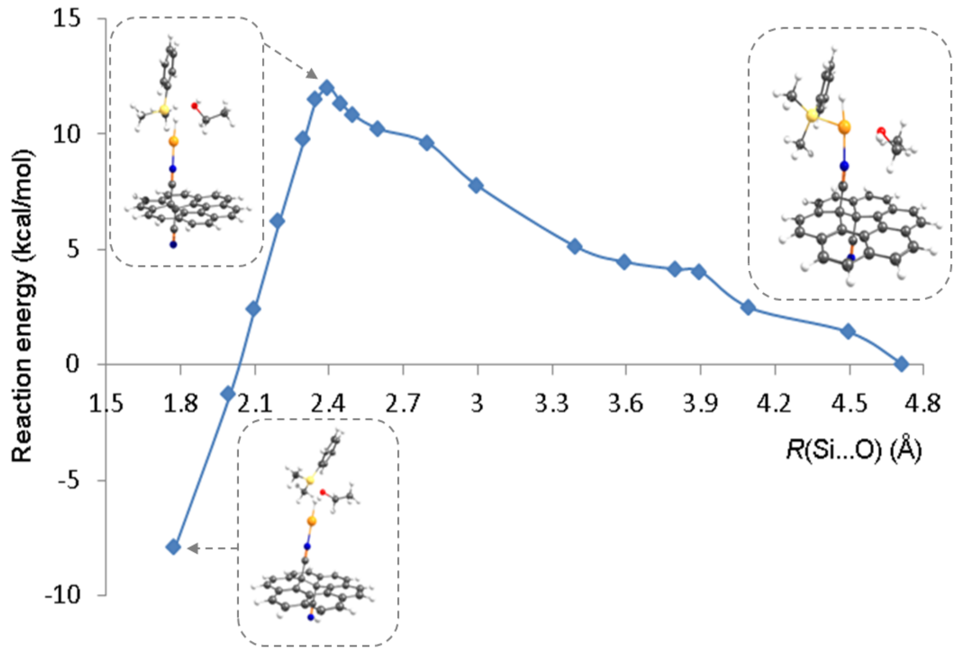

Figure S27. An indirect attack of an ethanol molecule on the silane ligand starting from a square planar configuration of the G(CN)-Au(I)...DMPS…ethanol complex in water (alternative to step 3 in Figure 3). Reaction energies (in kcal/mol) were obtained at the PBE0/Def2TZVP/SMD level of theory. Carbon atoms are grey, nitrogen blue, hydrogen white, oxygen red, gold orange, and silicon yellow.

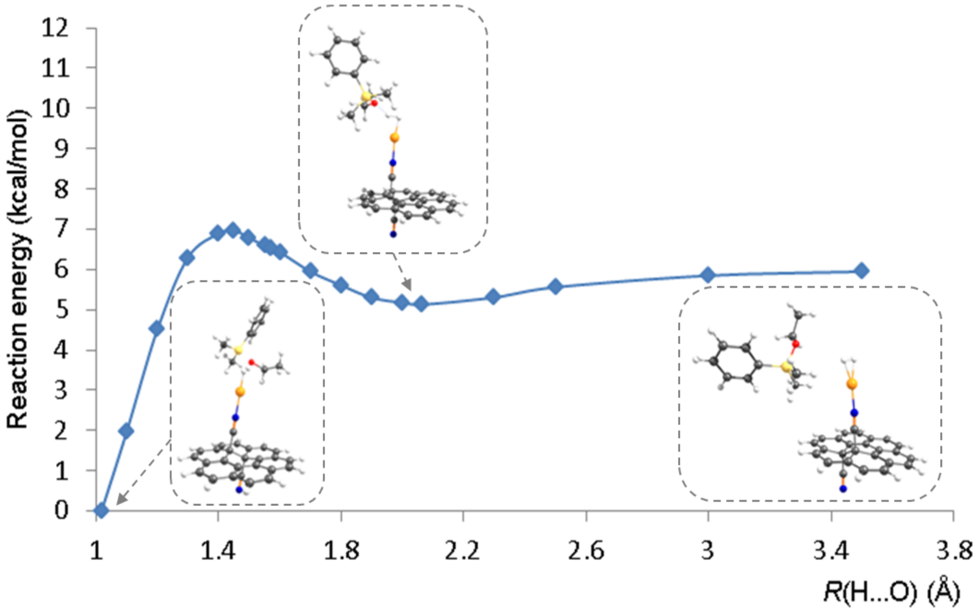

Figure S28. Deprotonation of a positively charged ethanol-silane complex in water leading to the formation of an intermediate with the hydrogen molecule bound to the Au(I) site (step 4) followed by the release of the product (step 5). Reaction energies (in kcal/mol) were obtained at the PBE0/Def2TZVP/SMD level of theory. Carbon atoms are grey, nitrogen blue, hydrogen white, oxygen red, gold orange, and silicon yellow.

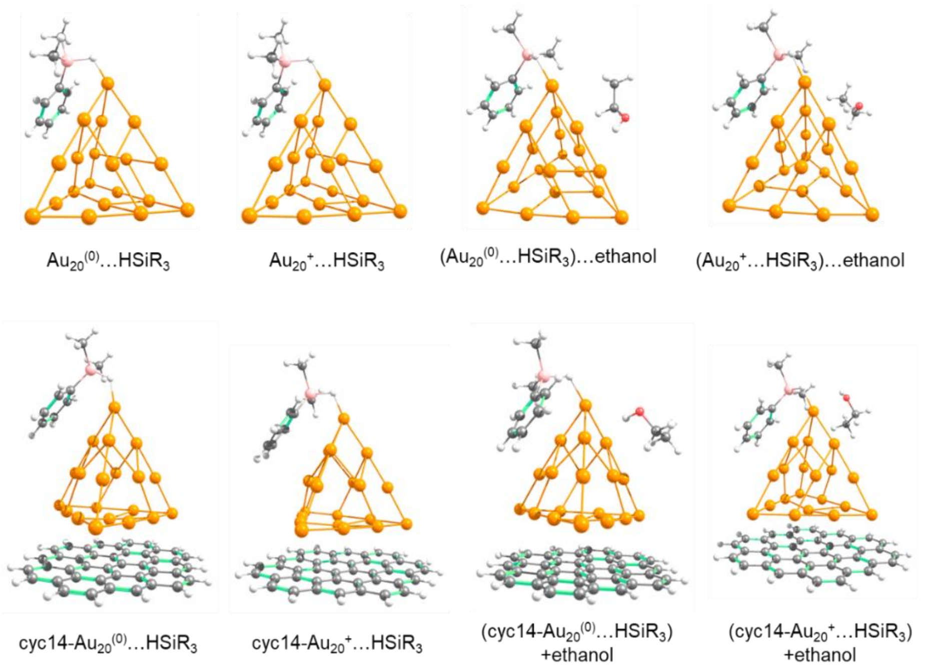

Figure S29. Structures of (Gr-)…Au_{20} interacting with silane and ethanol in water obtained at the SMD/PBE0-D3/DEF2-TZVP level of theory. Carbon atoms are grey, hydrogen white, oxygen red, gold orange, and silicon pink.

Commentary:

The calculations showed that the binding of Au_{20} nanoclusters on the graphene substrate is energetically favorable, and the charge of the nanocluster only slightly affects the BE (-53.1 and -56.2 kcal/mol for Au_{20} and Au_{20}^{+}, respectively). In both neutral and charged systems, charge transfer from the substrate to the nanocluster of ca. 0.6e was observed. The decrease of the charge of nanoclusters is reflected in the BE values for an attack by a silane molecule, which are by ~10-15 kcal/mol higher (more negative) for free solvated Au_{20} and Au_{20}^{+} compared to their counterparts grafted on graphene (Table S7). Let us recall that BE for the binding of silane on G(CN)-Au(I) is -32.5 kcal/mol, i.e., the binding of silane on catalytic centers is more favorable than that on nanoclusters stuck to the substrate. In the next reaction step, an ethanol molecule is supposed to come into contact with the silane moiety. Although the binding of ethanol on gold nanoclusters is energetically feasible, it preferably binds to a facet of the Au_{20}

tetrahedron rather than to its upper vortex which is occupied by the silane molecule (Figure S29) making the reaction sterically problematic. To sum up, the Au nanoclusters represent a stable form on the G-CN lattice, but the catalytic process predominantly takes advantage of the presence and nature of the single Au(I) ions occupying cyano groups of G-CN.

5. Supplementary Video

Supplementary Video 1. Hydrogen evolution Experiment. Experimental setup used for monitoring quantitative analysis of hydrogen for dehydrogenative coupling reaction with an inverted burette.

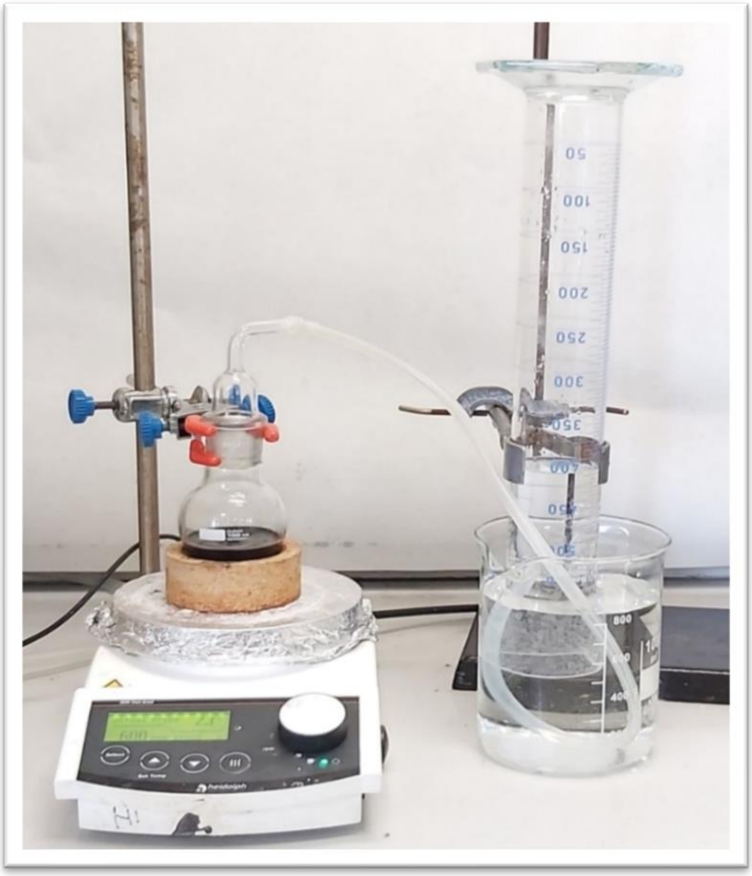

Hydrogen Evolution-Video.mp4

6. NMR and Mass spectra of compounds

^{1}H-NMR and ^{13}C-NMR spectra of products

Ethoxydimethylphenylsilane (2a)

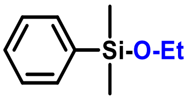

^{1}H-NMR (400 MHz, $CDCl_3$) δ 7.61 (td, J = 4.1, 1.2 Hz, 2H), 7.41 (q, J = 2.4 Hz, 3H), 3.70 (q, J = 7.0 Hz, 2H), 1.21 (t, J = 7.1 Hz, 3H), 0.41 (s, 6H) **^{13}C-NMR** (101 MHz, $CDCl_3$) δ 138.11, 133.58, 129.67, 127.95, 18.55, -1.62.

2a. Ethoxydimethylphenylsilane **^{1}H NMR** (400 MHz, $CDCl_3$)

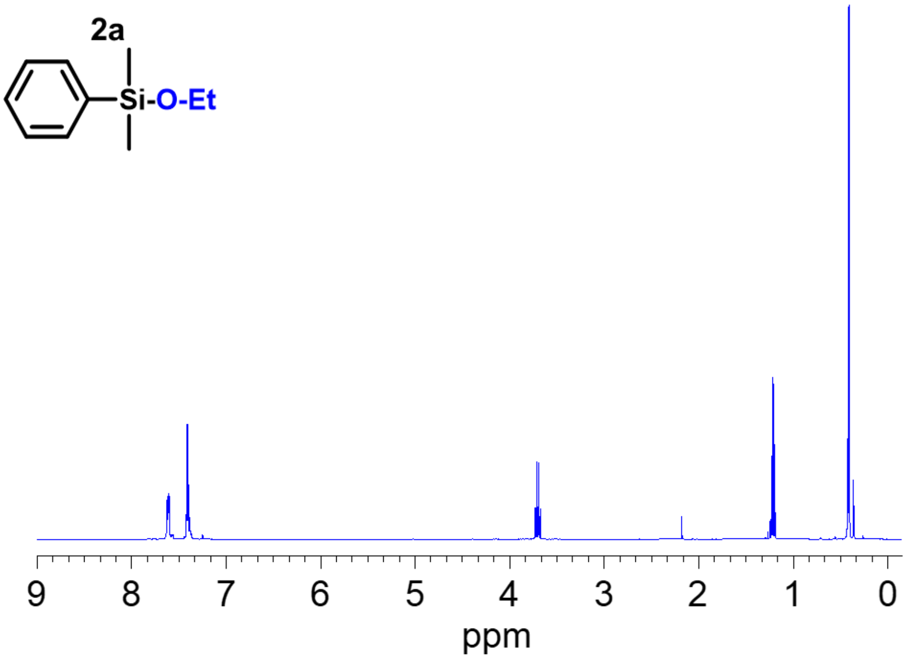

2a. Ethoxydimethylphenylsilane **^{13}C-NMR** (101 MHz, $CDCl_3$)

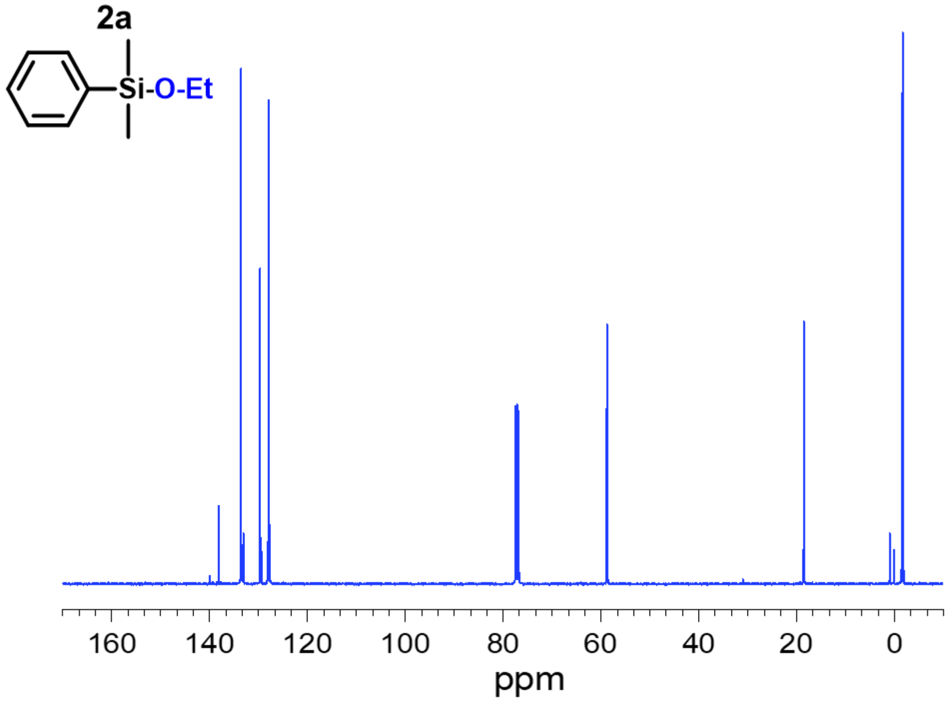

GC-MS spectra

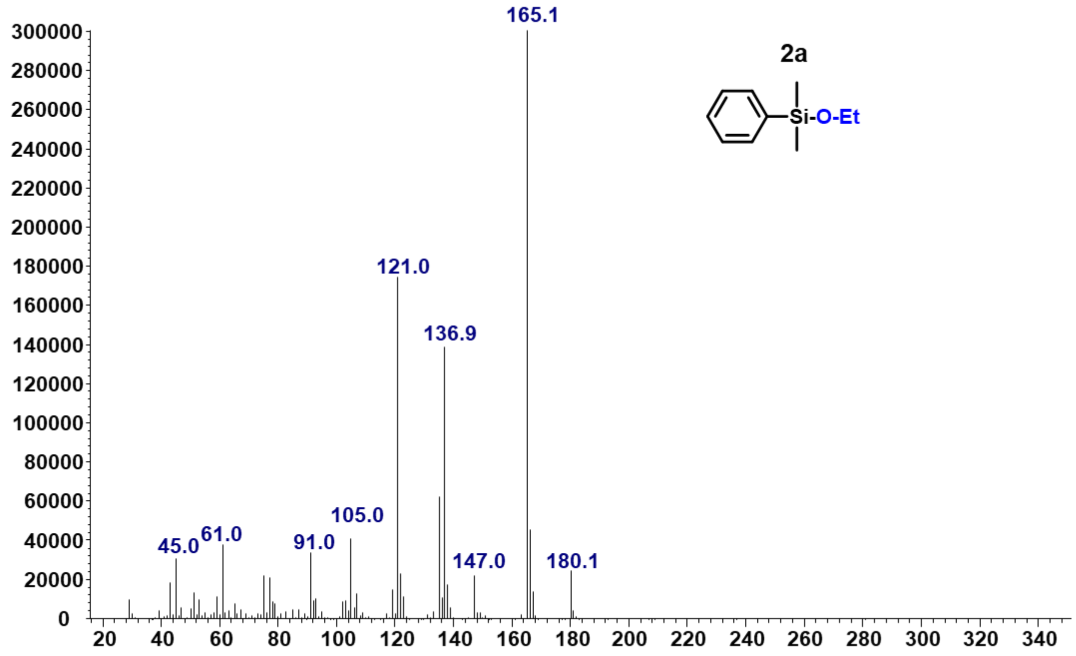

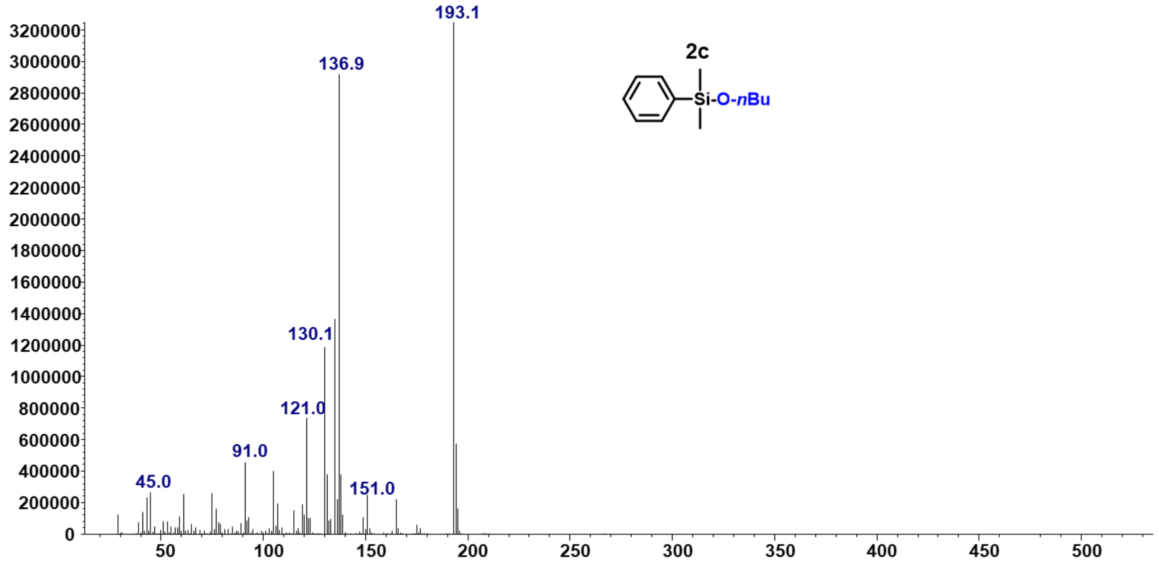

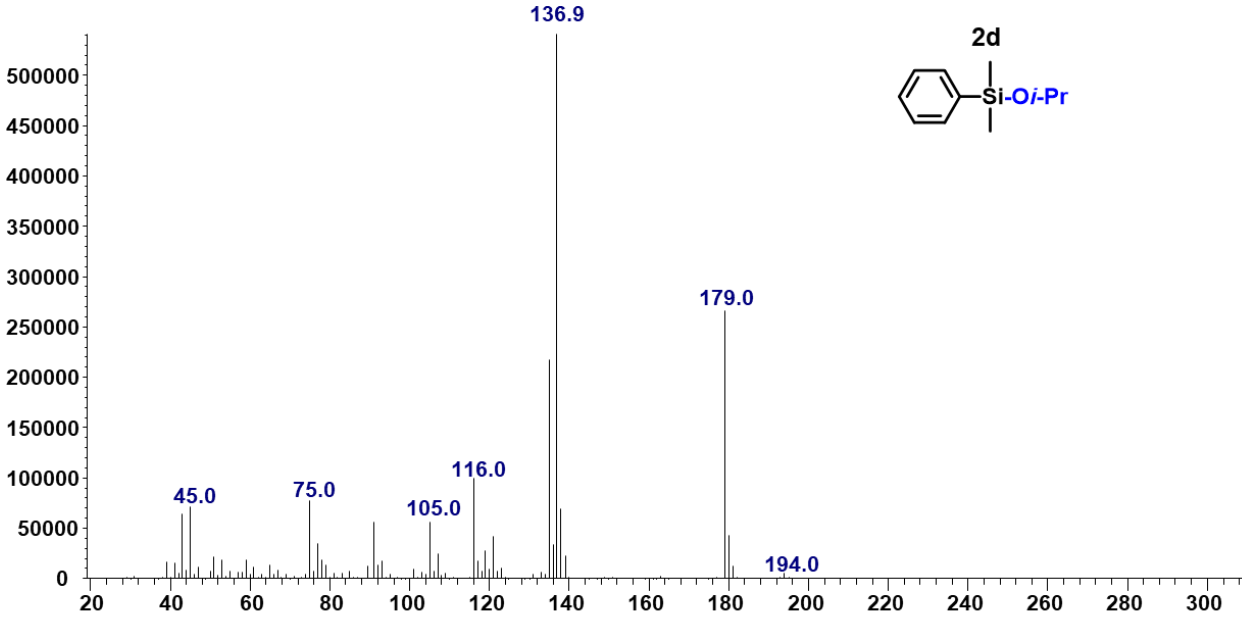
136.9
2d
Si-Oi-Pr
179.0
116.0
75.0
45.0
105.0
194.0

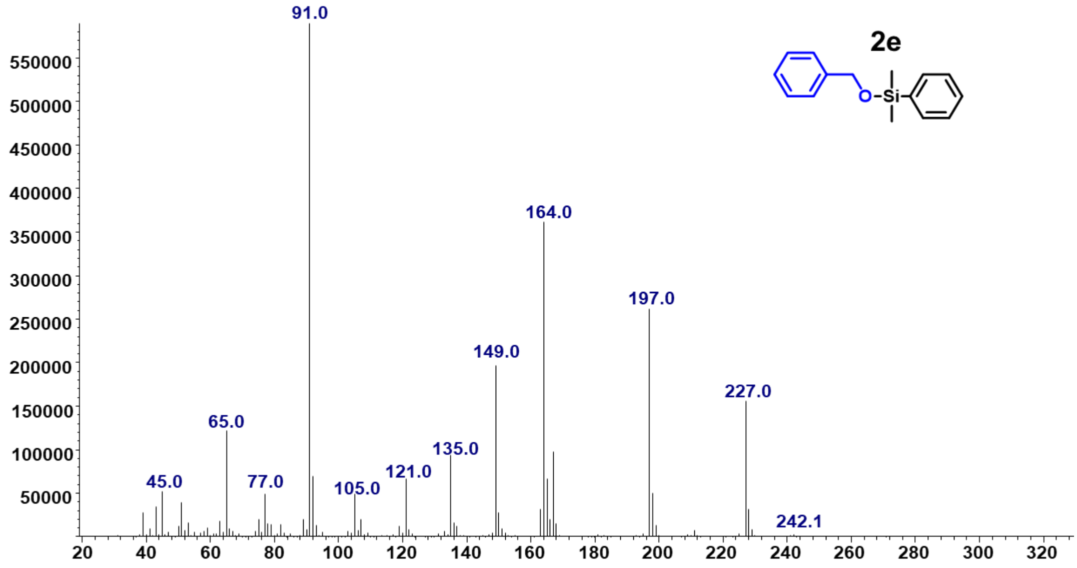
91.0
2e
O-Si
164.0
197.0
149.0
227.0
65.0
135.0
45.0
77.0
105.0
121.0
242.1

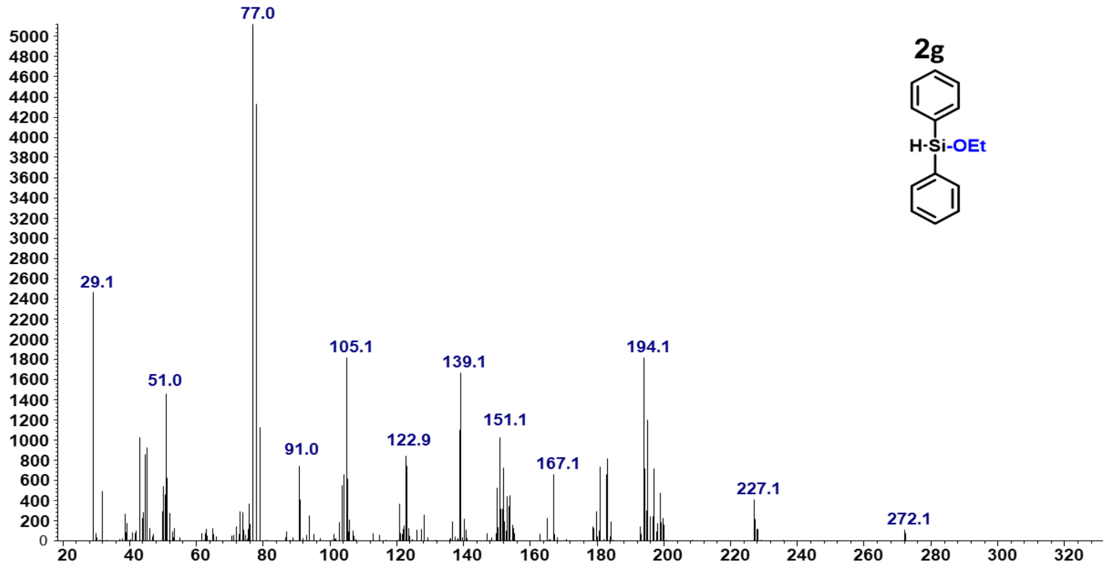
77.0
29.1
51.0
91.0
105.1
122.9
139.1
151.1
167.1
194.1
227.1
272.1
2g
H-Si-OEt

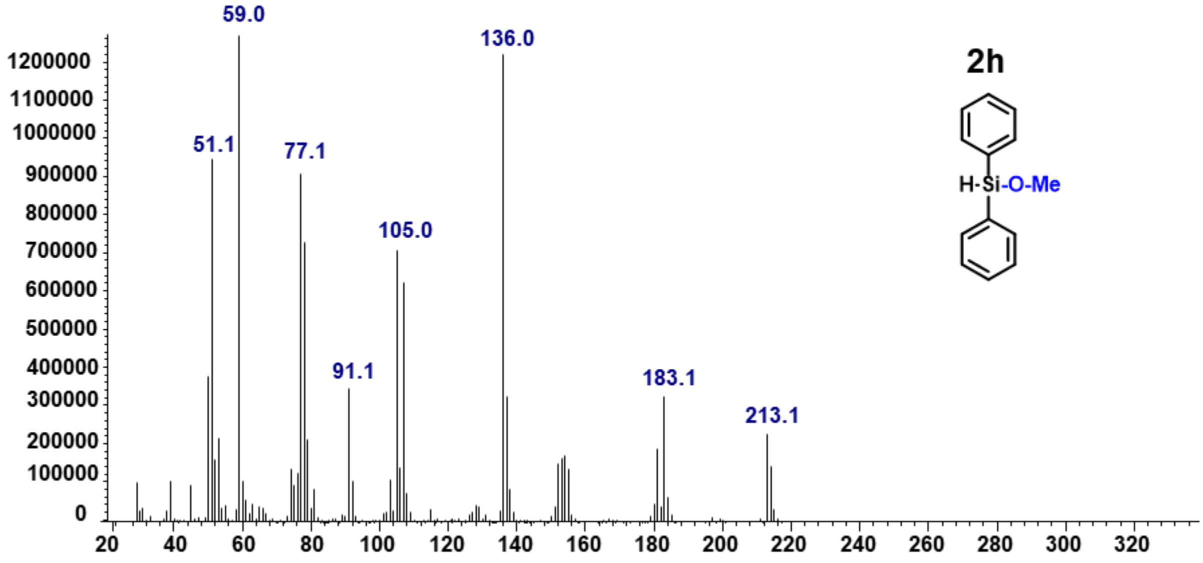
59.0
51.1
77.1
91.1
105.0
136.0
183.1
213.1
2h
H-Si-O-Me

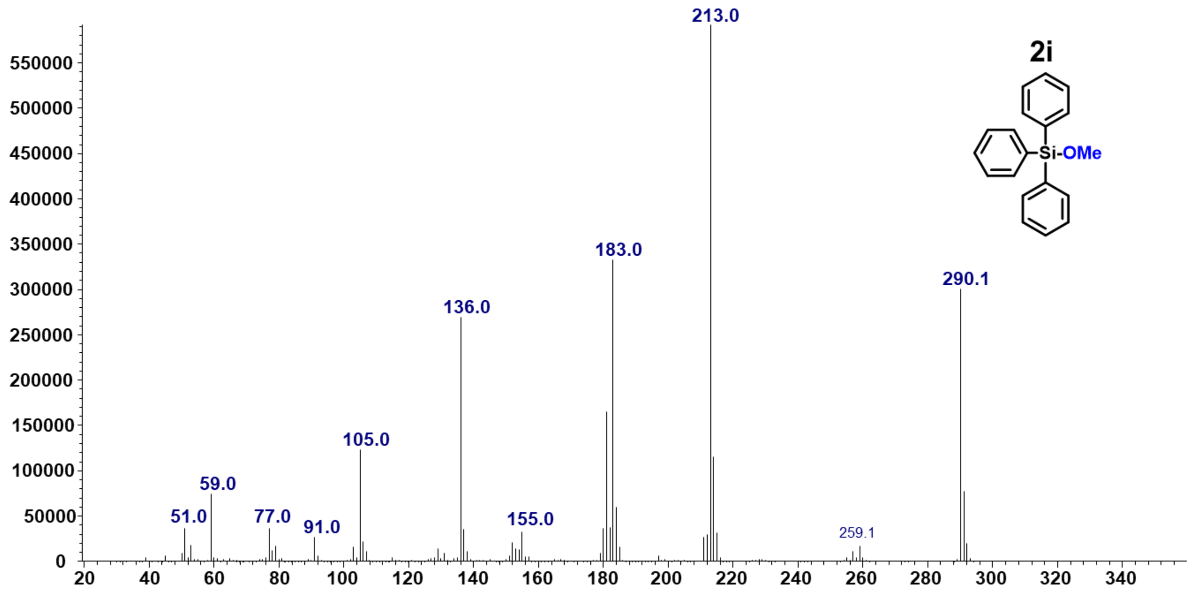
2i
Si-OMe
213.0
183.0
290.1
136.0
105.0
59.0
51.0
77.0
91.0
155.0
259.1

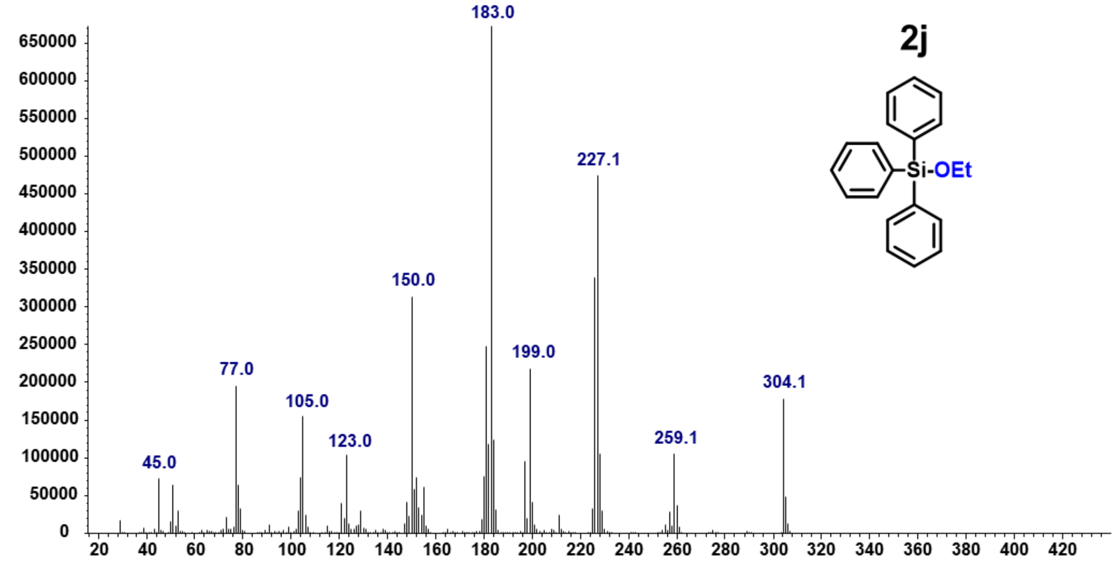
2j
Si-OEt
183.0
227.1
150.0
199.0
77.0
304.1
105.0
123.0
259.1
45.0

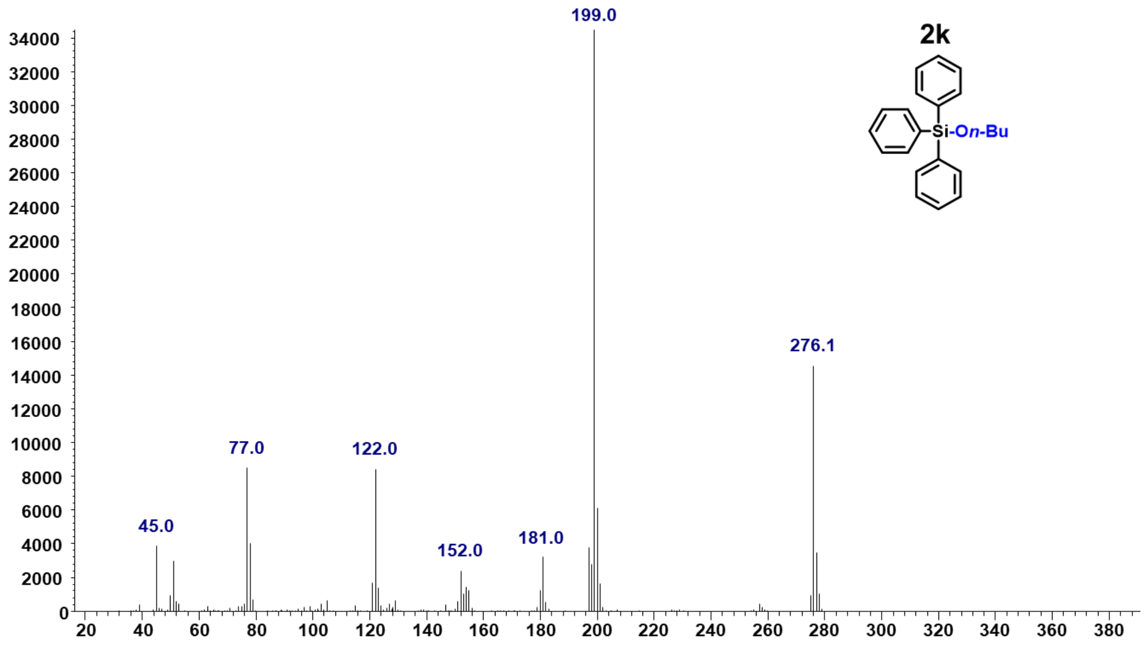
2k
Si-On-Bu
199.0
276.1
77.0
122.0
45.0
152.0
181.0

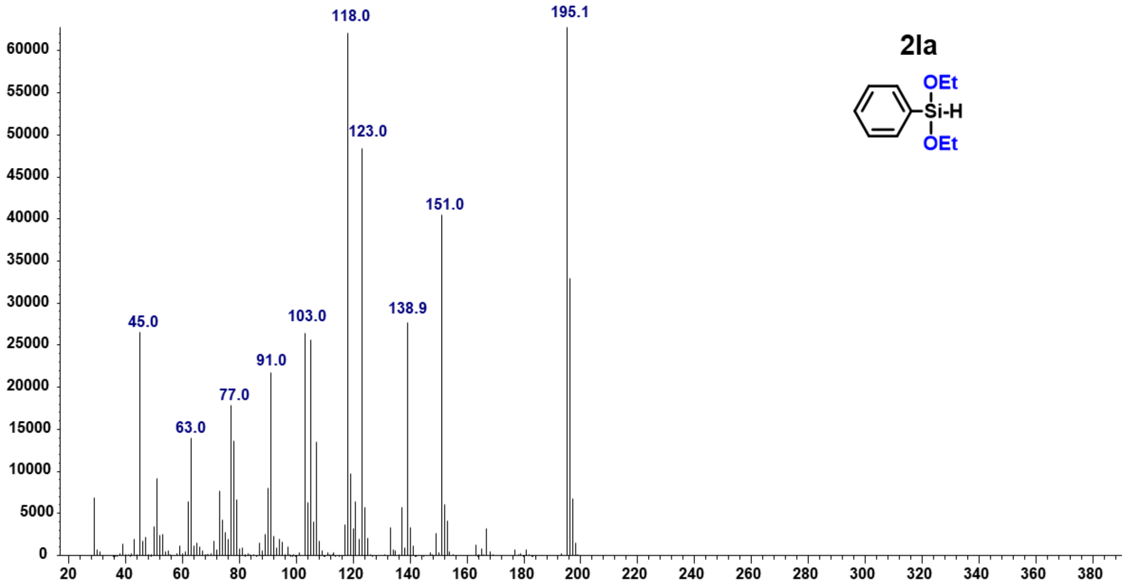
2la
OEt
Si-H
OEt
118.0
195.1
123.0
151.0
45.0
103.0
138.9
91.0
77.0
63.0

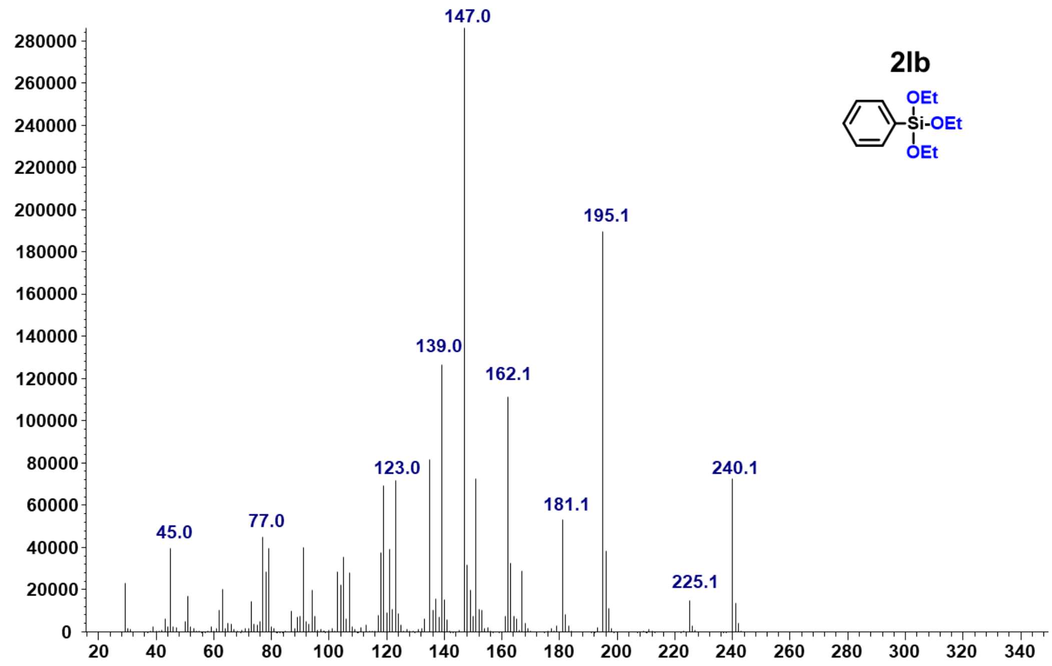
2lb
OEt
Si-OEt
OEt
147.0
195.1
139.0
162.1
123.0
240.1
181.1
77.0
45.0
225.1

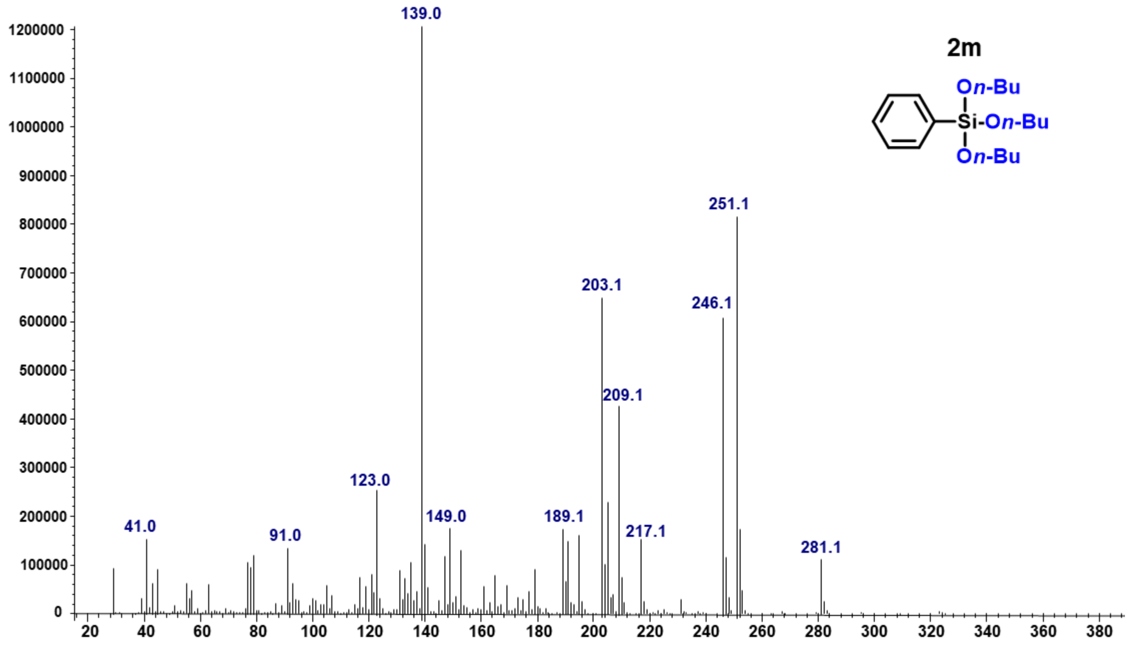
2m
On-Bu
Si-On-Bu
On-Bu
139.0
251.1
203.1
246.1
209.1
123.0
41.0
91.0
149.0
189.1
217.1
281.1

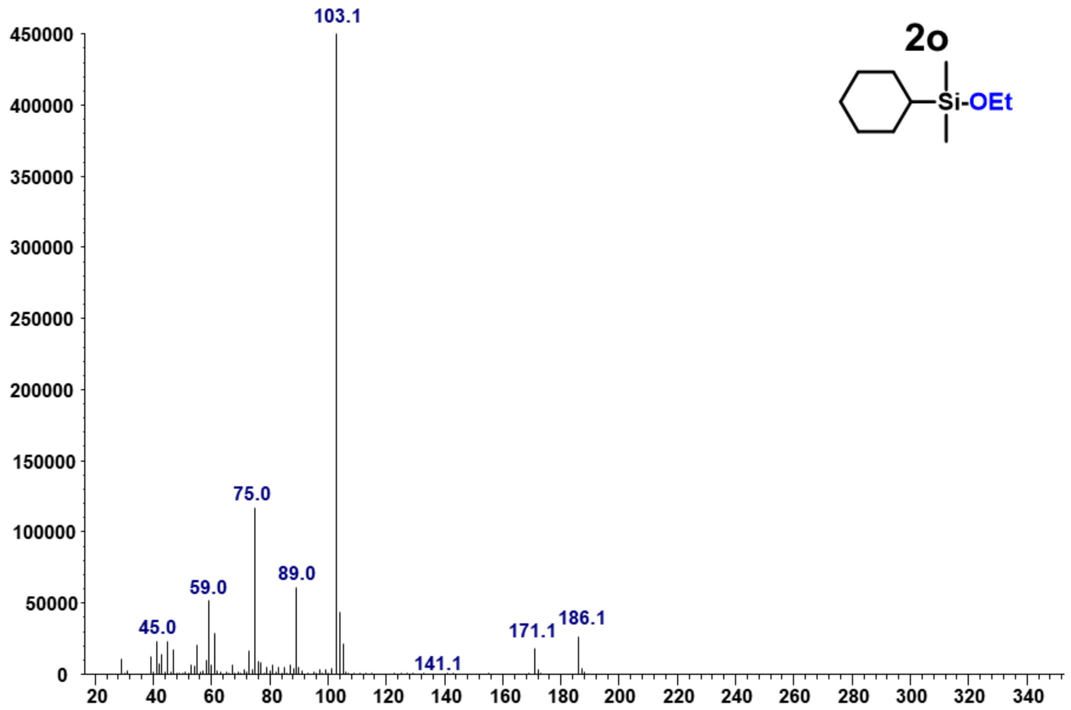
2o
Si-OEt
103.1
75.0
89.0
59.0
45.0
141.1
171.1
186.1
450000
400000
350000
300000
250000
200000
150000
100000
50000
0
20 40 60 80 100 120 140 160 180 200 220 240 260 280 300 320 340

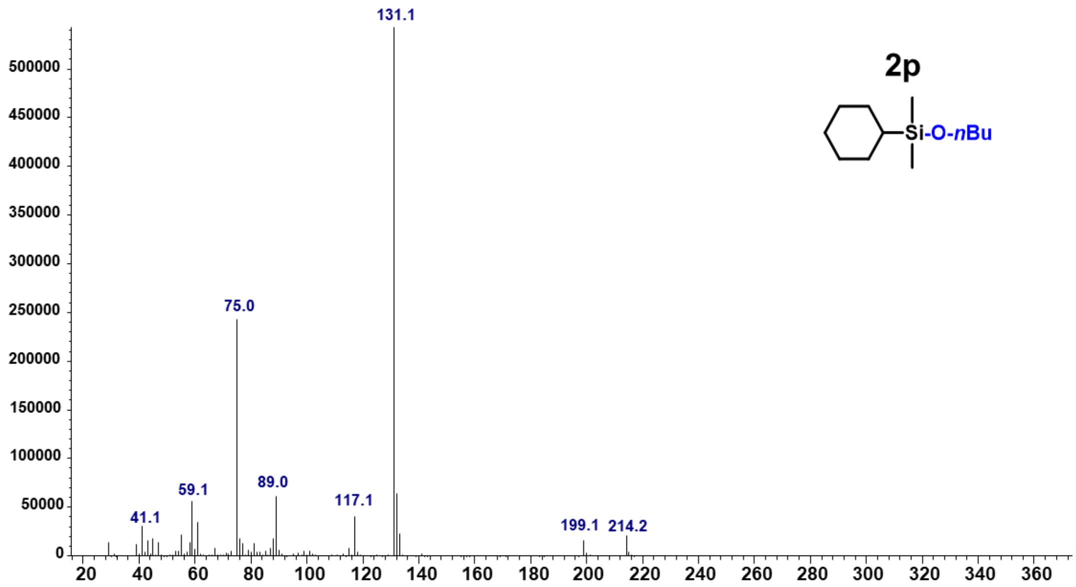
2p
Si-O-nBu
131.1
75.0
59.1
89.0
41.1
117.1
199.1
214.2
500000
450000
400000
350000
300000
250000
200000
150000
100000
50000
0
20 40 60 80 100 120 140 160 180 200 220 240 260 280 300 320 340 360

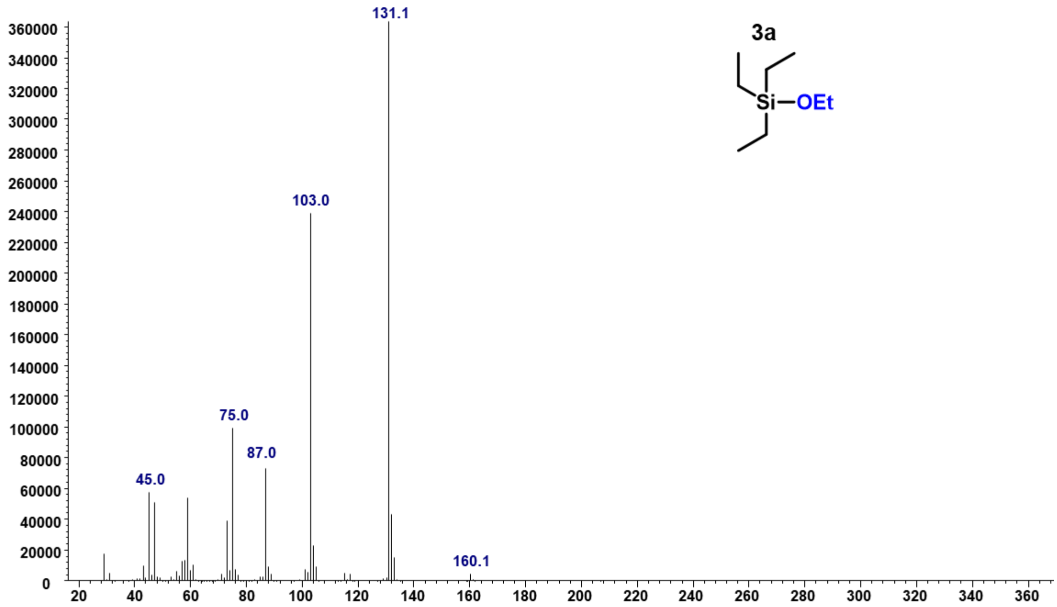

131.1
103.0
75.0
87.0
45.0
160.1
3a
Si
OEt

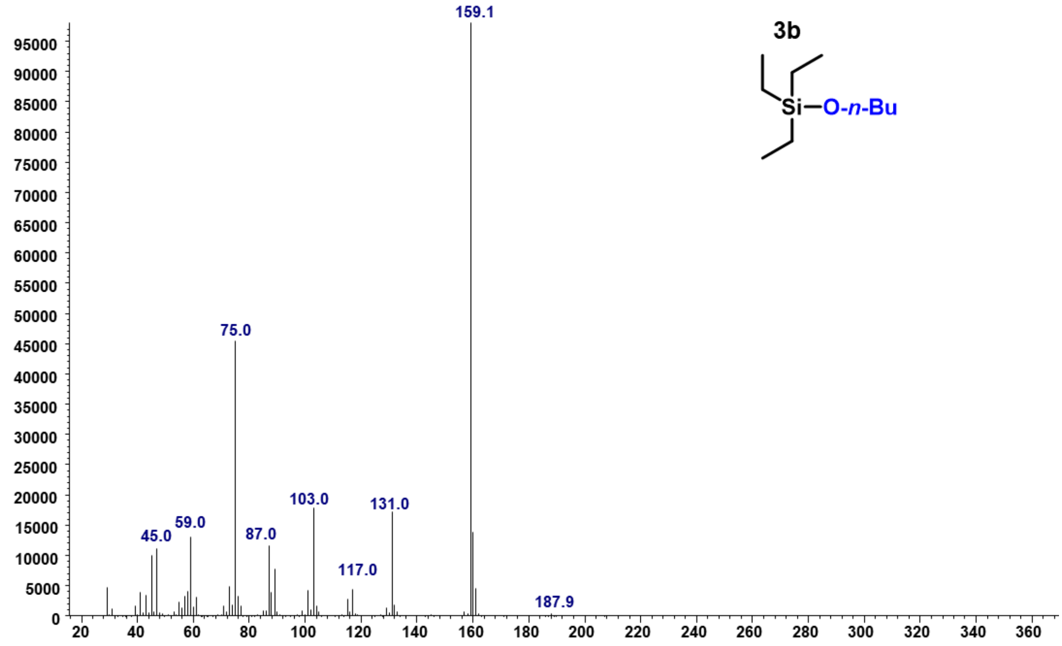

159.1
75.0
103.0
131.0
59.0
45.0
87.0
117.0
187.9
3b
Si
O-n-Bu

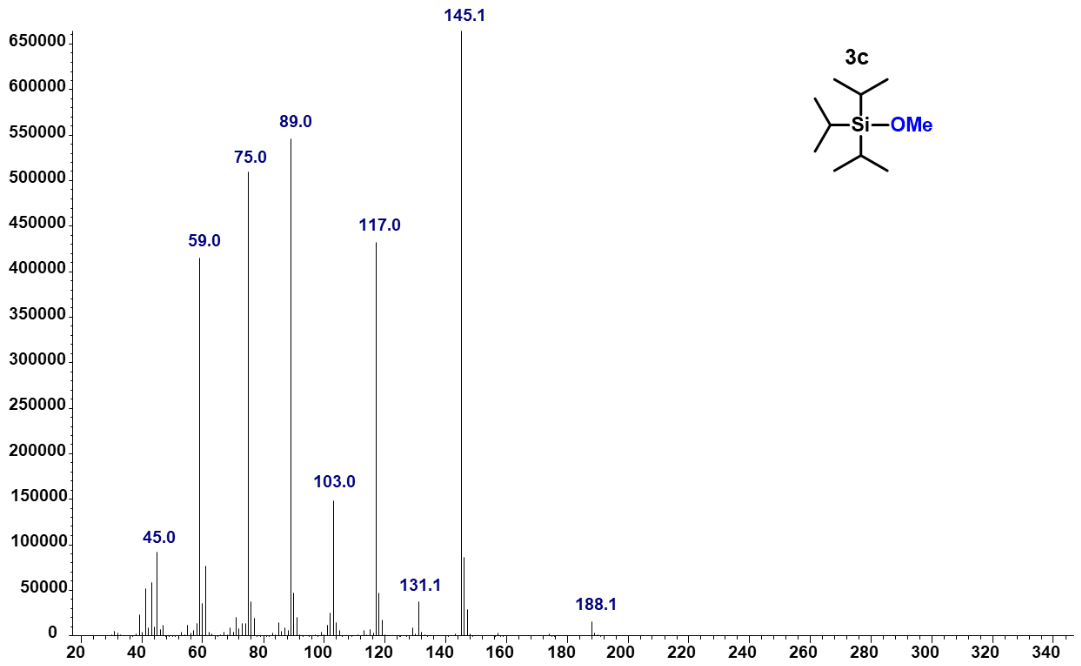
145.1
3c
Si
OMe
89.0
75.0
117.0
59.0
103.0
45.0
131.1
188.1
650000
600000
550000
500000
450000
400000
350000
300000
250000
200000
150000
100000
50000
0
20
40
60
80
100
120
140
160
180
200
220
240
260
280
300
320
340

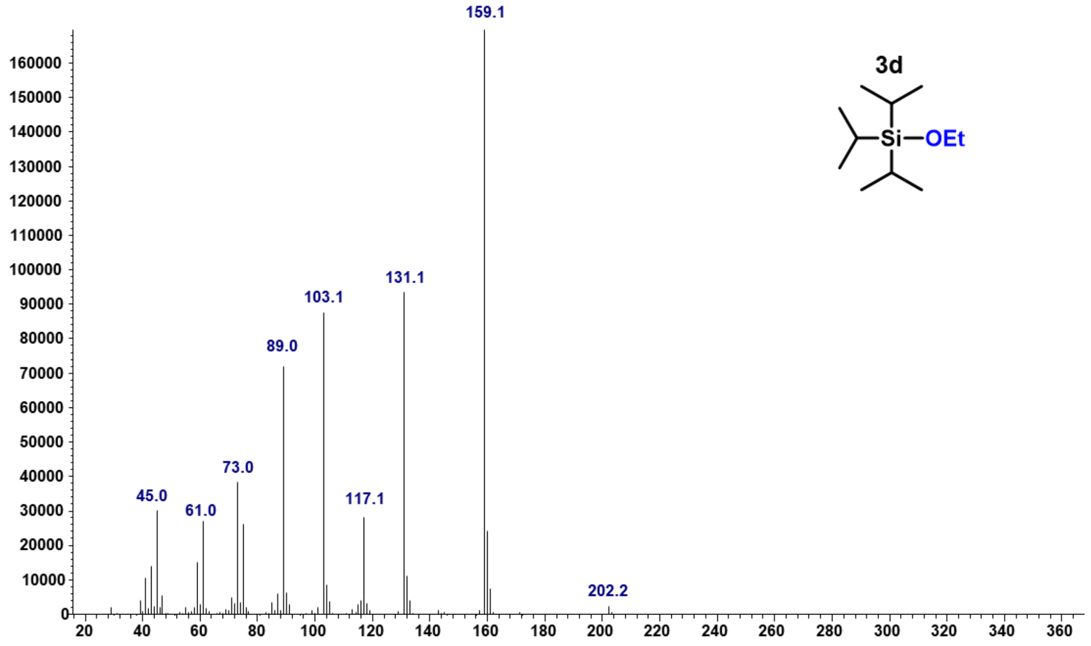
159.1
3d
Si
OEt
131.1
103.1
89.0
73.0
45.0
61.0
117.1
202.2
160000
150000
140000
130000
120000
110000
100000
90000
80000
70000
60000
50000
40000
30000
20000
10000
0
20
40
60
80
100
120
140
160
180
200
220
240
260
280
300
320
340
360

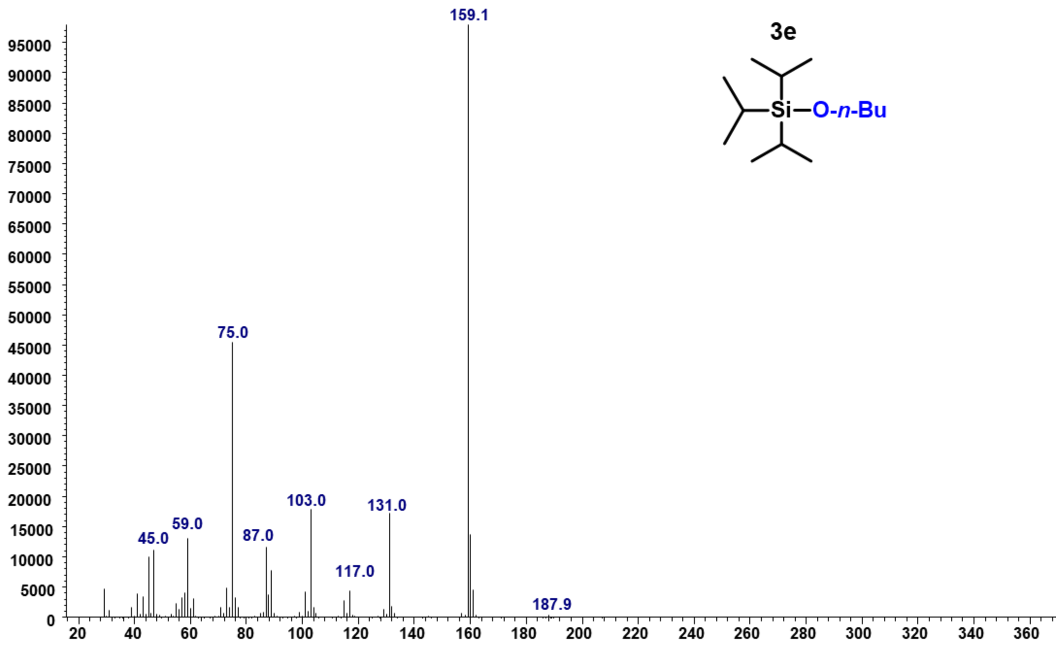
3e
Si
O-n-Bu
159.1
75.0
103.0
131.0
59.0
45.0
87.0
117.0
187.9

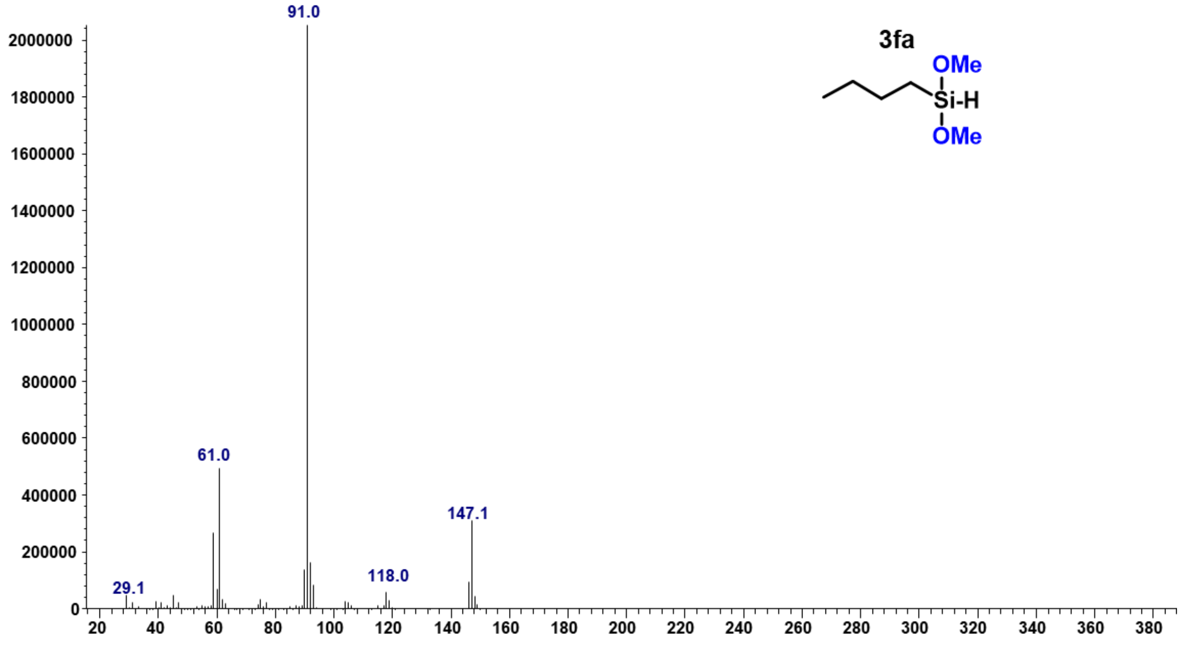
3fa
OMe
Si-H
OMe
91.0
61.0
147.1
118.0
29.1

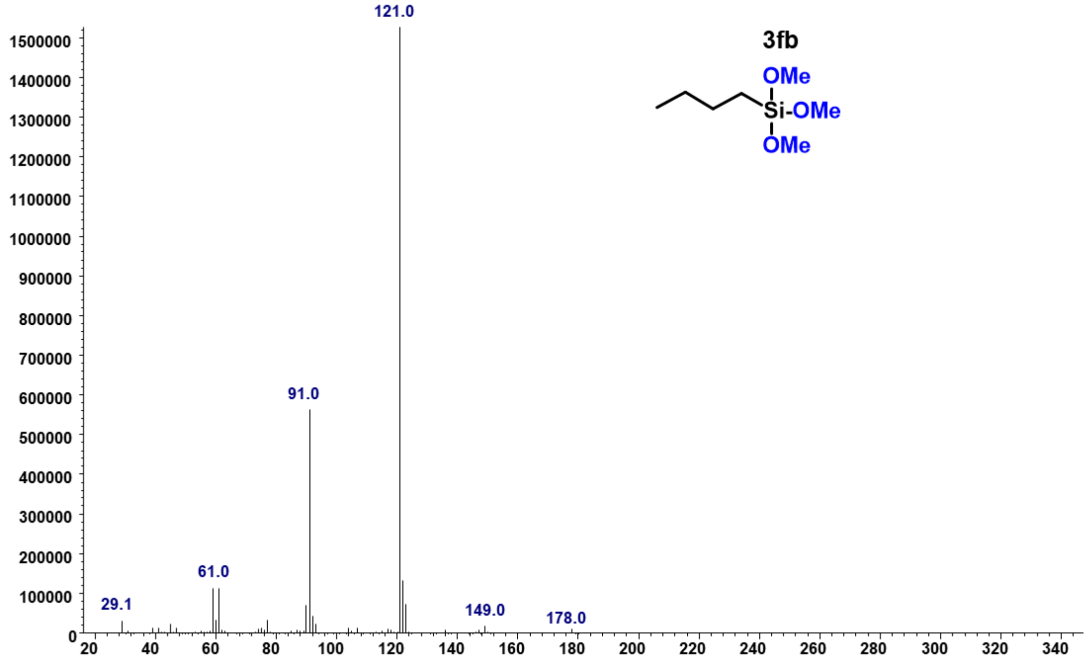

121.0
91.0
61.0
29.1
149.0
178.0
3fb
OMe
Si-OMe
OMe

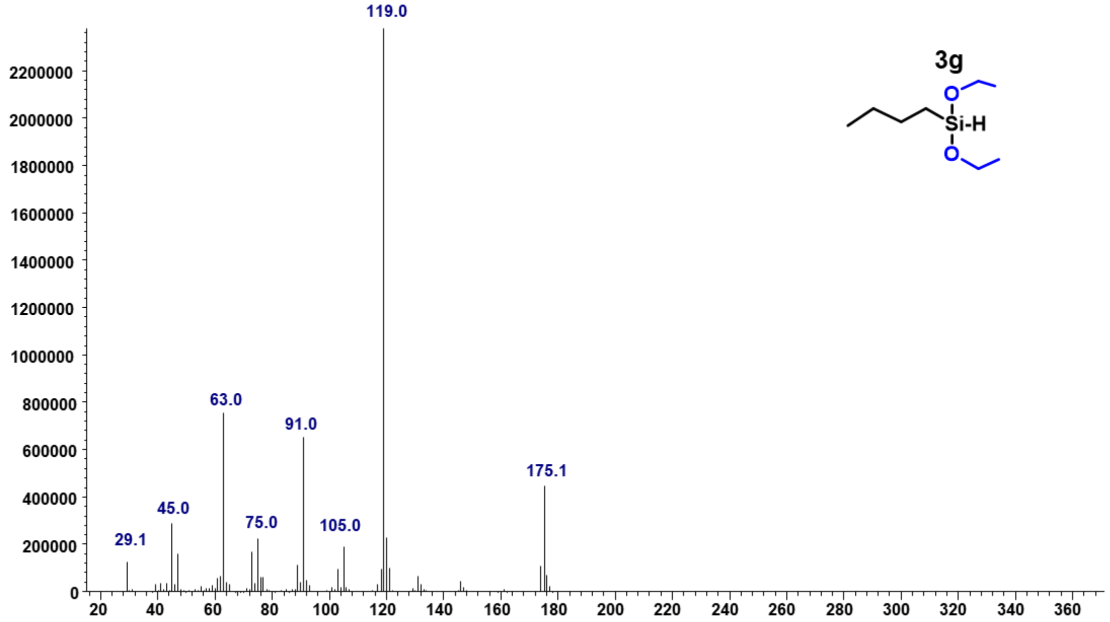

119.0
63.0
91.0
175.1
45.0
75.0
105.0
29.1
3g
O
Si-H
O

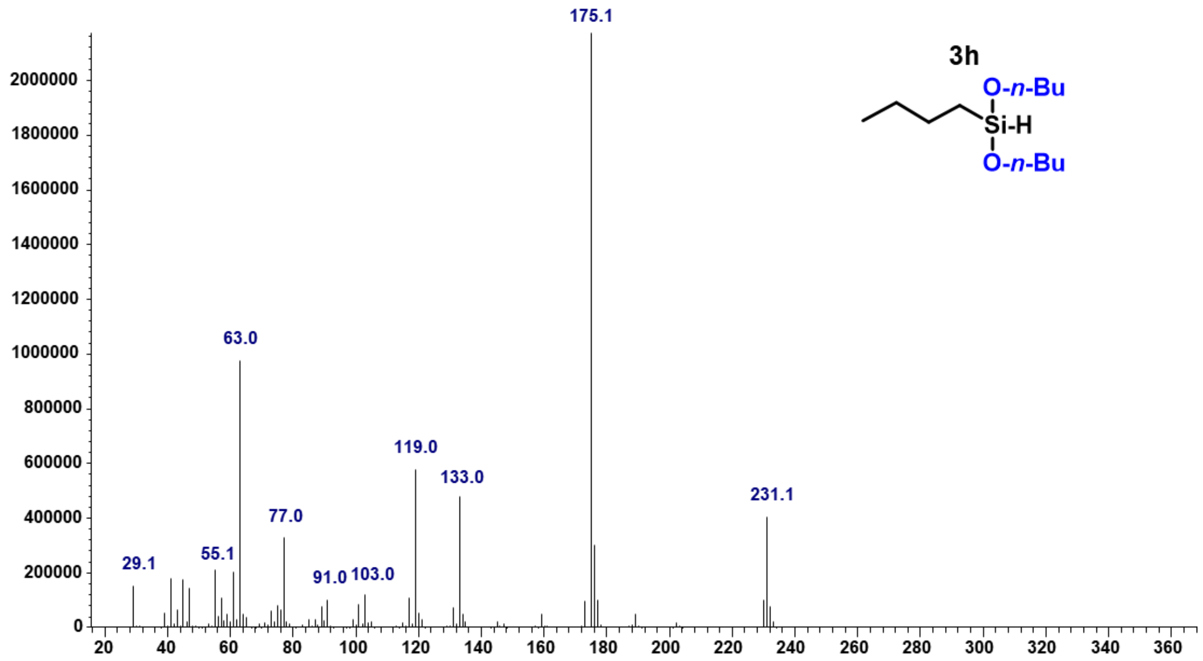
175.1
3h
O-n-Bu
Si-H
O-n-Bu
2000000
1800000
1600000
1400000
1200000
1000000
800000
600000
400000
200000
0
63.0
119.0
133.0
77.0
231.1
29.1
55.1
91.0
103.0
20
40
60
80
100
120
140
160
180
200
220
240
260
280
300
320
340
360

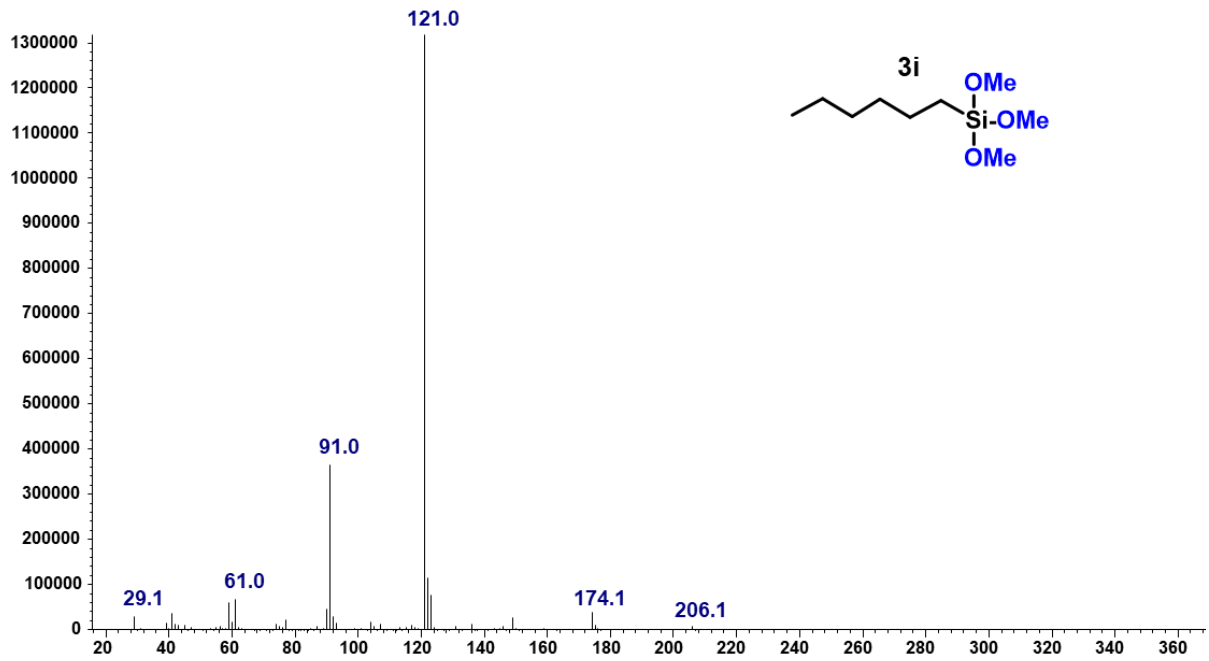
121.0
3i
OMe
Si-OMe
OMe
1300000
1200000
1100000
1000000
900000
800000
700000
600000
500000
400000
300000
200000
100000
0
91.0
29.1
61.0
174.1
206.1
20
40
60
80
100
120
140
160
180
200
220
240
260
280
300
320
340
360

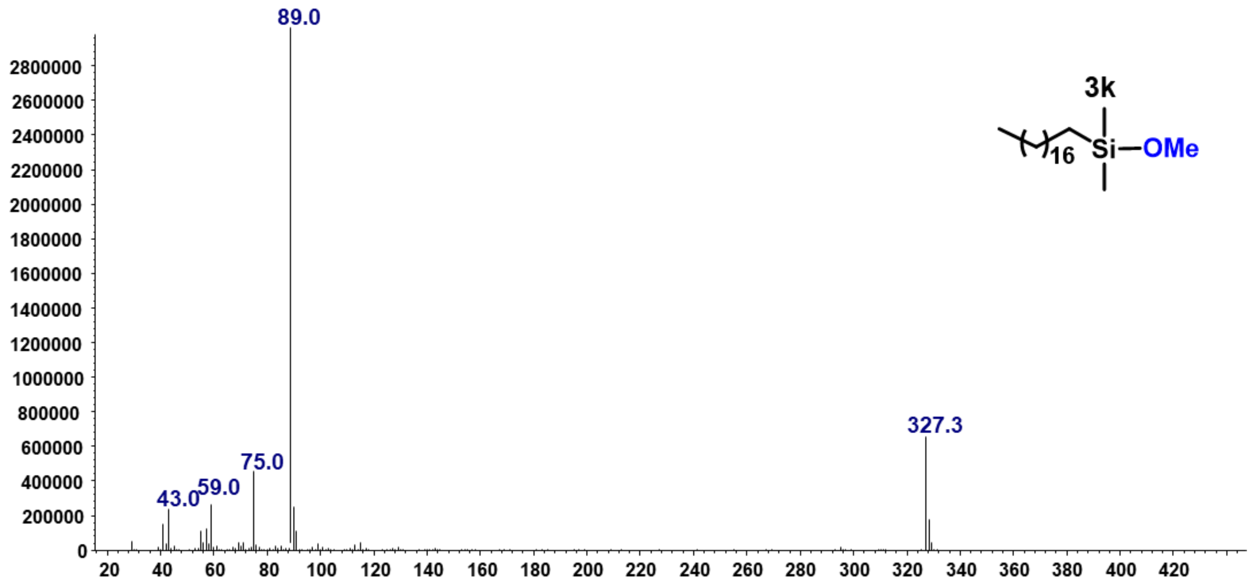
89.0
3k
16
Si
OMe
2800000
2600000
2400000
2200000
2000000
1800000
1600000
1400000
1200000
1000000
800000
600000
400000
200000
0
327.3
75.0
43.0
59.0
20 40 60 80 100 120 140 160 180 200 220 240 260 280 300 320 340 360 380 400 420

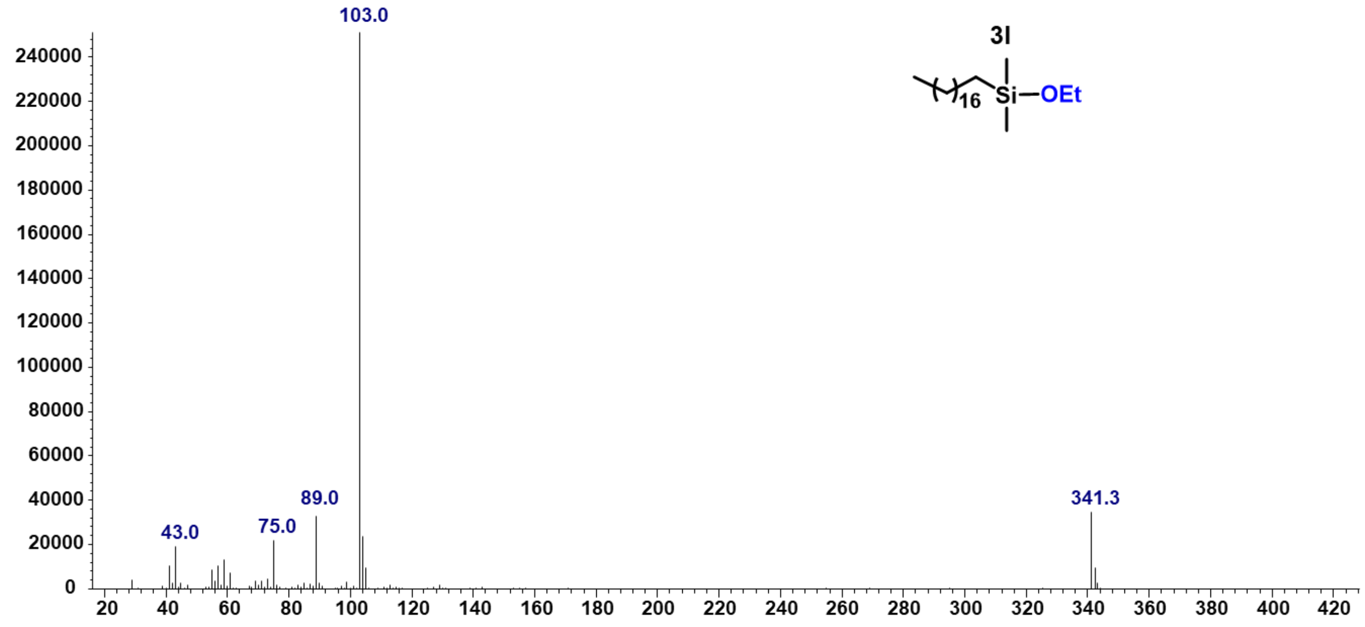
103.0
3l
16
Si
OEt
240000
220000
200000
180000
160000
140000
120000
100000
80000
60000
40000
20000
0
89.0
341.3
43.0
75.0
20 40 60 80 100 120 140 160 180 200 220 240 260 280 300 320 340 360 380 400 420

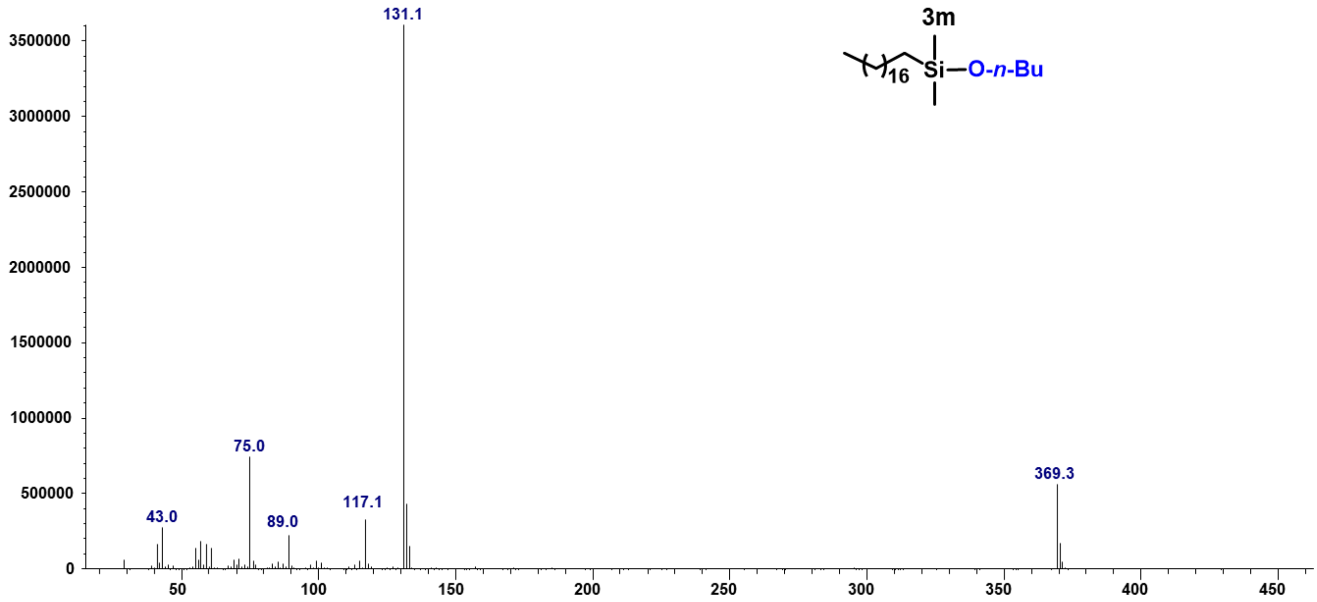
131.1
3m
16
Si
O-n-Bu
3500000
3000000
2500000
2000000
1500000
1000000
500000
0
75.0
43.0
89.0
117.1
369.3
50
100
150
200
250
300
350
400
450

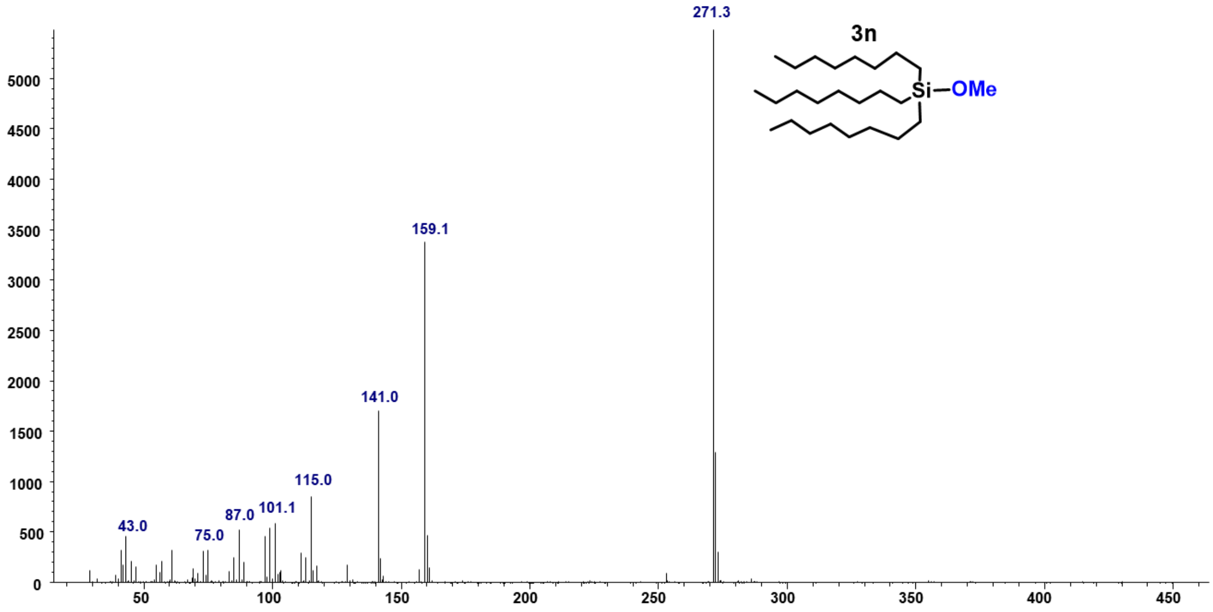
271.3
3n
Si
OMe
5000
4500
4000
3500
3000
2500
2000
1500
1000
500
0
159.1
141.0
115.0
43.0
75.0
87.0
101.1
50
100
150
200
250
300
350
400
450

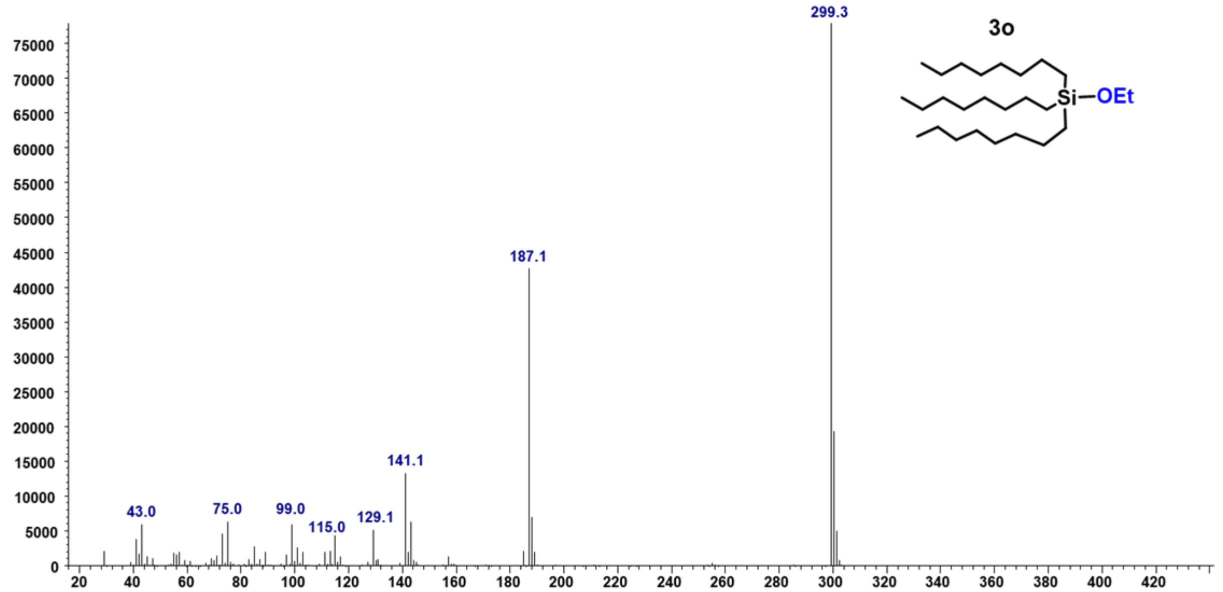
3o
Si—OEt
299.3
187.1
141.1
43.0
75.0
99.0
115.0
129.1

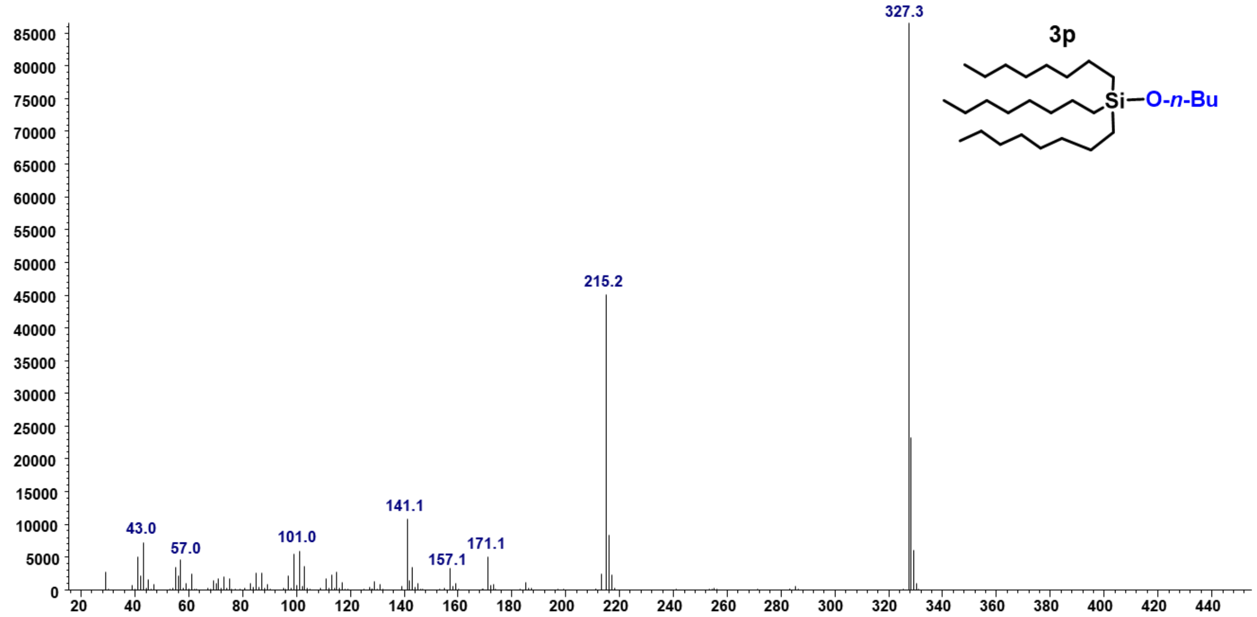
3p
Si—O-n-Bu
327.3
215.2
141.1
43.0
57.0
101.0
157.1
171.1

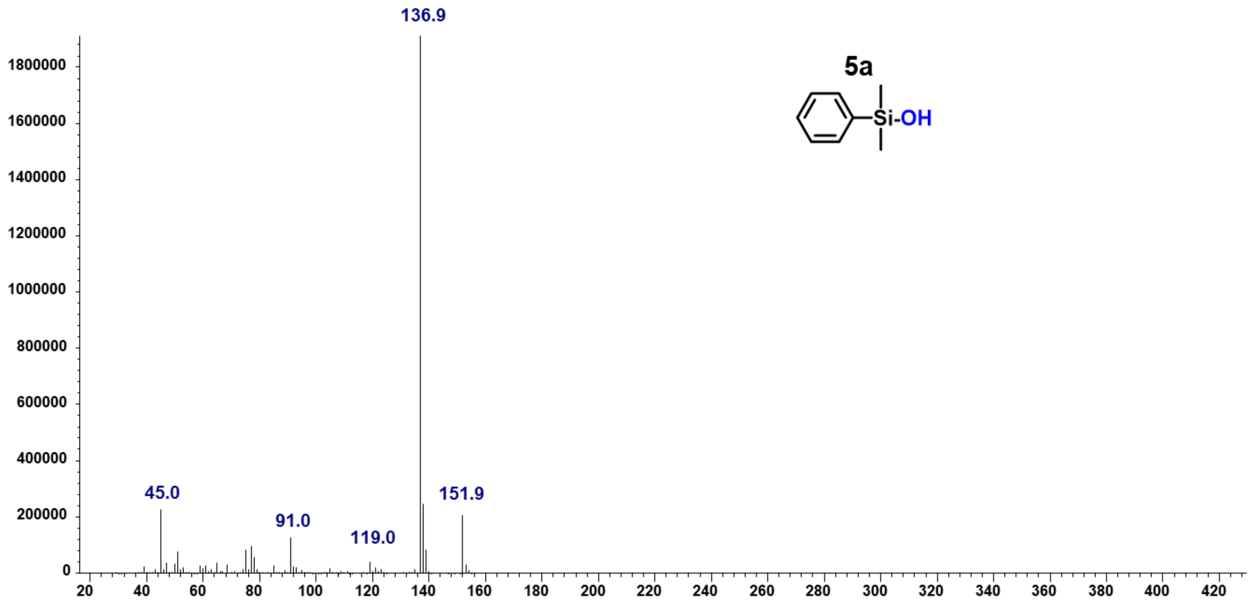
136.9
5a
Si-OH
45.0
91.0
119.0
151.9

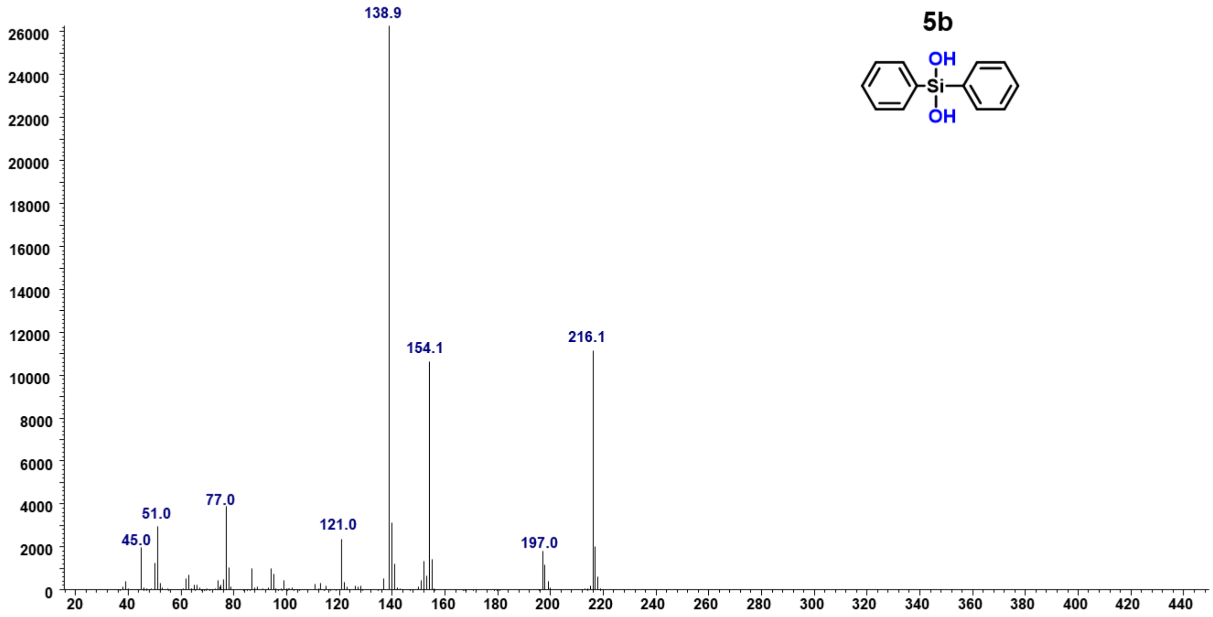
138.9
5b
OH
Si
OH
154.1
216.1
45.0
51.0
77.0
121.0
197.0

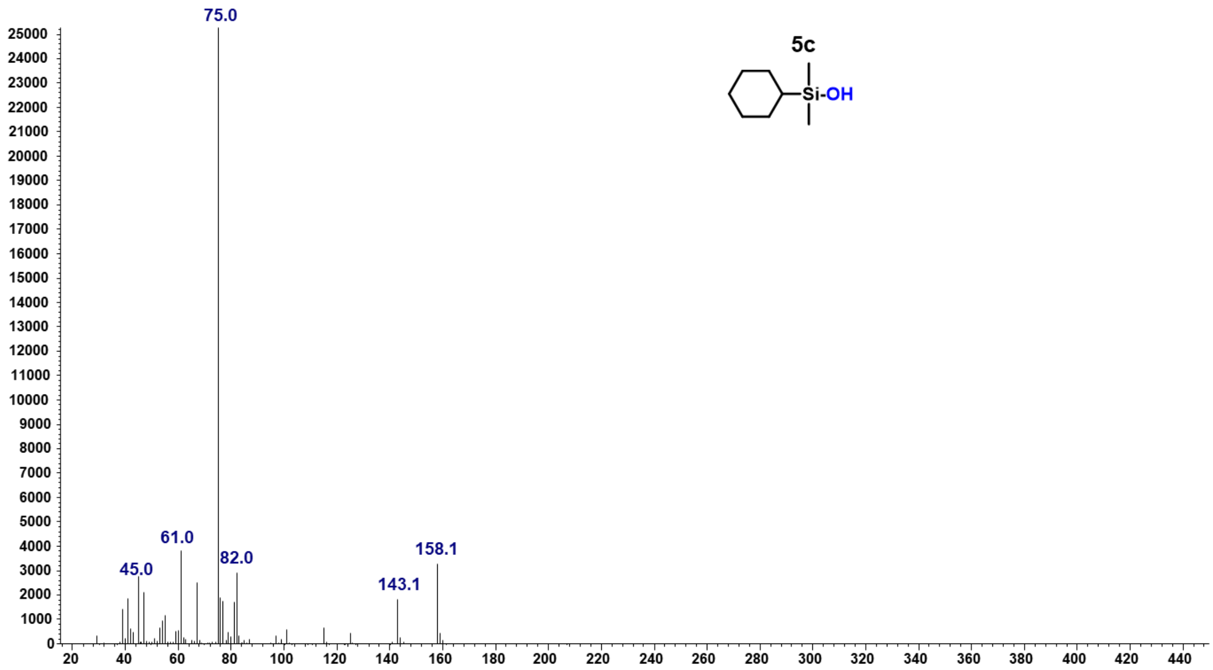
75.0
5c
Si-OH
61.0
45.0
82.0
158.1
143.1

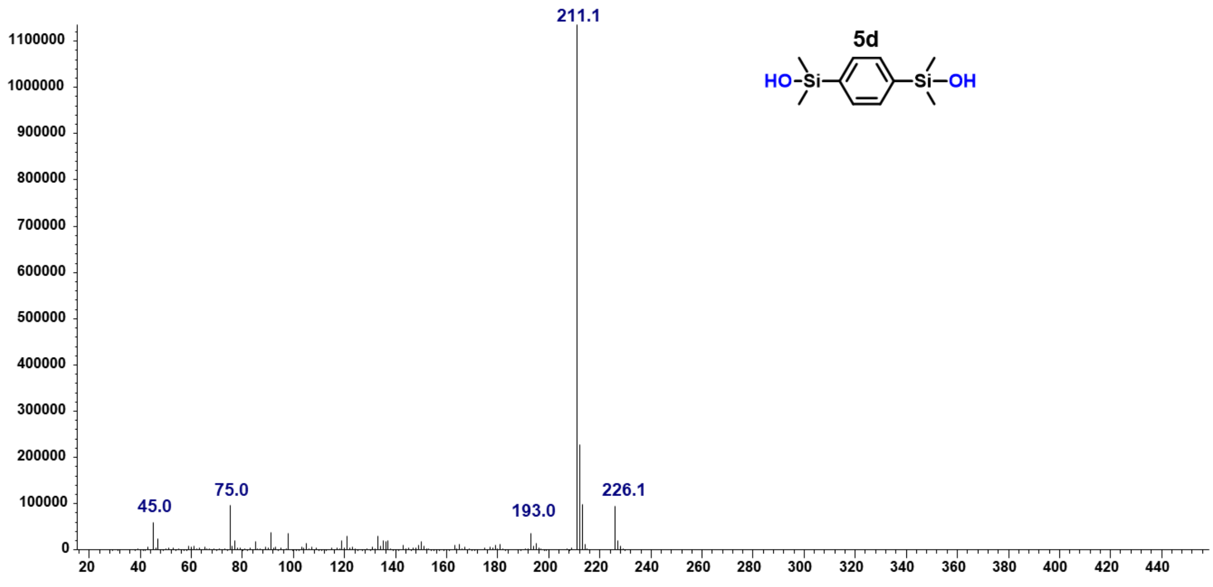
211.1
5d
HO-Si
Si-OH
45.0
75.0
193.0
226.1

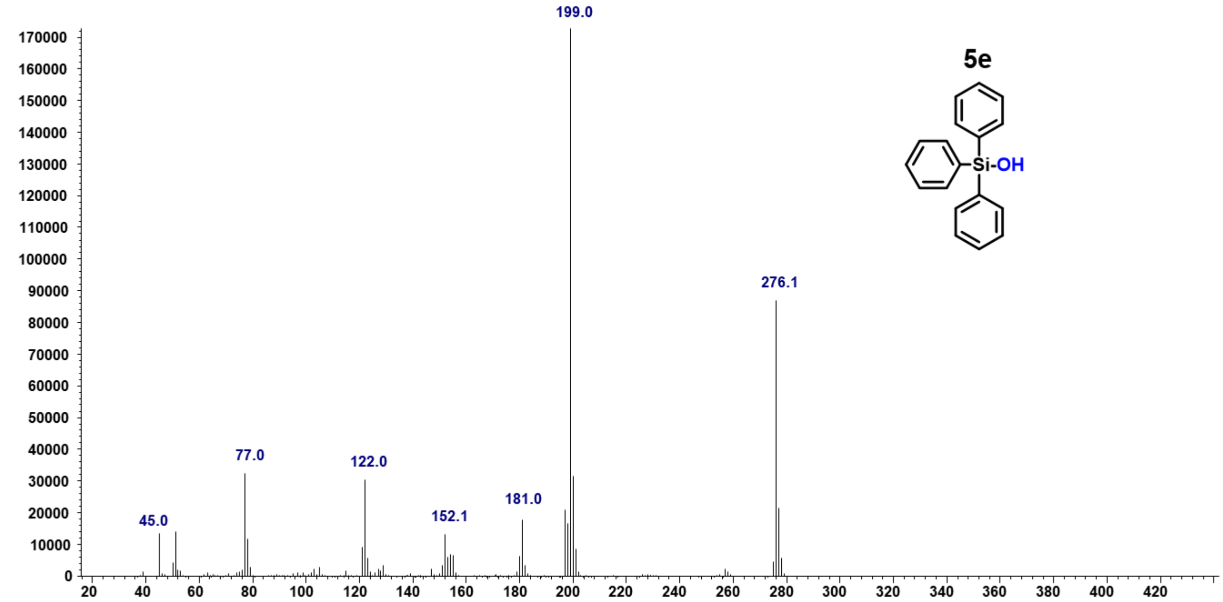
5e
Si-OH
199.0
276.1
77.0
122.0
152.1
181.0
45.0

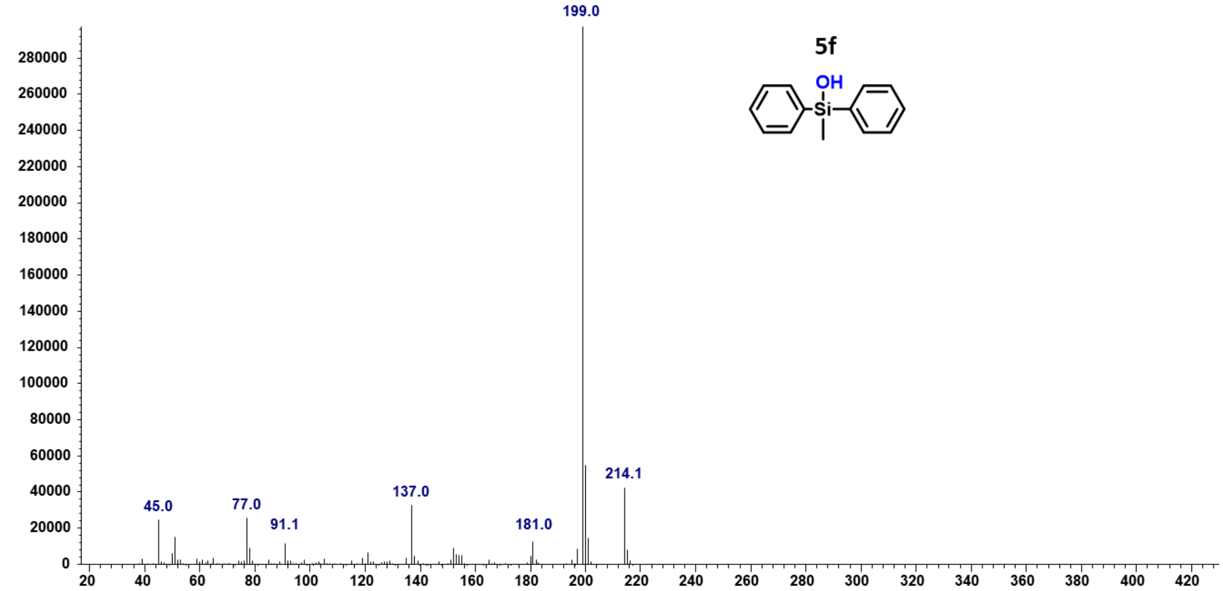
5f
OH
Si
199.0
214.1
137.0
45.0
77.0
91.1
181.0

7. References

(1) Bakandritsos, A.; Pykal, M.; Błoński, P.; Jakubec, P.; Chronopoulos, D. D.; Poláková, K.; Georgakilas, V.; Čépe, K.; Tomanec, O.; Ranc, V.; Bourlinos, A. B.; Zbořil, R.; Otyepka, M. Cyanographene and Graphene Acid: Emerging Derivatives Enabling High-Yield and Selective Functionalization of Graphene. *ACS Nano* **2017**, *11*, 2982-2991.

(2) Briois, V.; Fonda, E.; Belin, S.; Barthe, L.; La Fontaine, C.; Langlois, F.; Ribbens, M.; Villain, F. SAMBA: The 4–40 keV X-ray Absorption Spectroscopy Beamline at SOLEIL. *UVX 2010 - 10e Colloque sur les Sources Cohérentes et Incohérentes UV, VUV et X ; Applications et Développements Récents* **2011**, 41-47.

(3) Ravel, B.; Newville, M. ATHENA, ARTEMIS, HEPHAESTUS: data analysis for X-ray absorption spectroscopy using IFEFFIT. *J. Synchrotron Rad.* **2005**, *12*, 537-541.

(4) Adamo, C.; Barone, V. Toward Reliable Density Functional Methods Without Adjustable Parameters: The PBE0 model. *J. Chem. Phys.* **1999**, *110*, 6158-6170.

(5) Weigend, F.; Ahlrichs, R. Balanced Basis Sets of Split Valence, Triple Zeta Valence and Quadruple Zeta Valence Quality for H to Rn: Design and assessment of accuracy. *Phys. Chem. Chem. Phys.* **2005**, *7*, 3297-3305.

(6) Pašteka, L. F.; Rajský, T.; Urban, M. Toward Understanding the Bonding Character in Complexes of Coinage Metals with Lone-Pair Ligands. CCSD(T) and DFT Computations. *J. Phys. Chem. A* **2013**, *117*, 4472-4485.

(7) Marenich, A. V.; Cramer, C. J.; Truhlar, D. G. Universal Solvation Model Based on Solute Electron Density and on a Continuum Model of the Solvent Defined by the Bulk Dielectric Constant and Atomic Surface Tensions. *J. Phys. Chem. B* **2009**, *113*, 6378-6396.

(8) Marenich, A. V.; Jerome, S. V.; Cramer, C. J.; Truhlar, D. G. Charge Model 5: An Extension of Hirshfeld Population Analysis for the Accurate Description of Molecular Interactions in Gaseous and Condensed Phases. *J. Chem. Theory Comput.* **2012**, *8*, 527-541.

(9) Douglas, M.; Kroll, N. M. Quantum Electrodynamical Corrections To The Fine Structure Of Helium. *Ann. Phys.* **1974**, *82*, 89-155.

(10) Hess, B. A. Relativistic Electronic-Structure Calculations Employing a Two-Component No-Pair Formalism with External-Field Projection Operators. *Phys. Rev. A* **1986**, *33*, 3742-3748.

(11) Nakajima, T.; Hirao, K. The Douglas–Kroll–Hess Approach. *Chem. Rev.* **2012**, *112*, 385-402.

(12) Roos, B. O.; Lindh, R.; Malmqvist, P.-Å.; Veryazov, V.; Widmark, P.-O. New Relativistic ANO Basis Sets for Transition Metal Atoms. *J. Phys. Chem. A* **2005**, *109*, 6575-6579.

(13) Pritchard, B. P.; Altarawy, D.; Didier, B.; Gibson, T. D.; Windus, T. L. New Basis Set Exchange: An Open, Up-to-Date Resource for the Molecular Sciences Community. *J. Chem. Inf. Model.***2019**, *59*, 4814-4820.

(14) Frisch, M. J.; Trucks, G. W.; Schlegel, H. B.; Scuseria, G. E.; Robb, M. A.; Cheeseman, J. R.; Scalmani, G.; Barone, V.; Petersson, G. A.; Nakatsuji, H.; Li, X.; Caricato, M.; Marenich, A. V.; Bloino, J.; Janesko, B. G.; Gomperts, R.; Mennucci, B.; Hratchian, H. P.; Ortiz, J. V.; Izmaylov, A. F.; Sonnenberg, J. L.; Williams; Ding, F.; Lipparini, F.; Egidi, F.; Goings, J.; Peng, B.; Petrone, A.; Henderson, T.; Ranasinghe, D.; Zakrzewski, V. G.; Gao, J.; Rega, N.; Zheng, G.; Liang, W.; Hada, M.; Ehara, M.; Toyota, K.; Fukuda, R.; Hasegawa, J.; Ishida, M.; Nakajima, T.; Honda, Y.; Kitao, O.; Nakai, H.; Vreven, T.; Throssell, K.; Montgomery Jr., J. A.; Peralta, J. E.; Ogliaro, F.; Bearpark, M. J.; Heyd, J. J.; Brothers, E. N.; Kudin, K. N.; Staroverov, V. N.; Keith, T. A.; Kobayashi, R.; Normand, J.; Raghavachari, K.; Rendell, A. P.; Burant, J. C.; Iyengar, S. S.; Tomasi, J.; Cossi, M.; Millam, J. M.; Klene, M.;

Adamo, C.; Cammi, R.; Ochterski, J. W.; Martin, R. L.; Morokuma, K.; Farkas, O.; Foresman, J. B.; Fox, D. J.: Gaussian 16 Rev. B.01. Wallingford, CT, 2016.

(15) Grimme, S. Semiempirical GGA-Type Density Functional Constructed with A Long-Range Dispersion Correction. *J. Comput. Chem.* **2006**, *27*, 1787-1799.

(16) Blöchl, P. E. Projector Augmented-Wave Method. *Phys. Rev. B* **1994**, *50*, 17953-17979.

(17) Kresse, G.; Furthmüller, J. Efficient Iterative Schemes for Ab Initio Total-Energy Calculations Using a Plane-Wave Basis Set. *Phys. Rev. B* **1996**, *54*, 11169-11186.

(18) Monkhorst, H. J.; Pack, J. D. Special Points for Brillouin-Zone Integrations. *Phys. Rev. B* **1976**, *13*, 5188-5192.

(19) Kresse, G.; Furthmüller, J. Efficiency of ab-initio Total Energy Calculations for Metals and Semiconductors Using a Plane-Wave Basis Set. *Comp. Mater. Sci* **1996**, *6*, 15-50.

(20) Kresse, G.; Joubert, D. From Ultrasoft Pseudopotentials to the Projector Augmented-Wave Method. *Phys. Rev. B* **1999**, *59*, 1758-1775.

(21) Li, J.; Li, X.; Zhai, H.-J.; Wang, L.-S. Au_{20}: A Tetrahedral Cluster . *Science* **2003**, *299*, 864.

(22) Sorribes, I.; Ventura-Espinosa, D.; Assis, M.; Martín, S.; Concepción, P.; Bettini, J.; Longo, E.; Mata, J. A.; Andrés, J. Unraveling a Biomass-Derived Multiphase Catalyst for the Dehydrogenative Coupling of Silanes with Alcohols under Aerobic Conditions. *ACS Sustainable Chem. Eng.* **2021**, *9*, 2912-2928.

(23) Wang, X.; Li, P.; Li, Z.; Chen, W.; Zhou, H.; Zhao, Y.; Wang, X.; Zheng, L.; Dong, J.; Lin, Y.; Zheng, X.; Yan, W.; Yang, J.; Yang, Z.; Qu, Y.; Yuan, T.; Wu, Y.; Li, Y. 2D MOF Induced Accessible and Exclusive Co Single Sites for an Efficient O-Silylation of Alcohols with Silanes. *Chem. Commun.* **2019**, *55*, 6563-6566.

(24) Dhiman, M.; Chalke, B.; Polshettiwar, V. Organosilane Oxidation with a Half Million Turnover Number using Fibrous Nanosilica Supported Ultrasmall Nanoparticles and Pseudo-Single Atoms of Gold. *Journal of Materials Chemistry A* **2017**, *5*, 1935-1940.

(25) Dhakshinamoorthy, A.; Concepcion, P.; Garcia, H. Dehydrogenative Coupling of Silanes with Alcohols Catalyzed by $Cu_3(BTC)_2$. *Chem. Commun.* **2016**, *52*, 2725-2728.

(26) Blandez, J. F.; Primo, A.; Asiri, A. M.; Álvaro, M.; García, H. Copper Nanoparticles Supported on Doped Graphenes as Catalyst for the Dehydrogenative Coupling of Silanes and Alcohols. *Angew. Chem. Int. Ed.* **2014**, *53*, 12581-12586.

(27) Mitsudome, T.; Yamamoto, Y.; Noujima, A.; Mizugaki, T.; Jitsukawa, K.; Kaneda, K. Highly Efficient Etherification of Silanes by Using a Gold Nanoparticle Catalyst: Remarkable Effect of O_2. *Chem. Eur. J.* **2013**, *19*, 14398-14402.

(28) Taguchi, T.; Isozaki, K.; Miki, K. Enhanced Catalytic Activity of Self-Assembled-Monolayer-Capped Gold Nanoparticles. *Adv. Mater.* **2012**, *24*, 6462-6467.

(29) Hara, K.; Akiyama, R.; Takakusagi, S.; Uosaki, K.; Yoshino, T.; Kagi, H.; Sawamura, M. Self-Assembled Monolayers of Compact Phosphanes with Alkanethiolate Pendant Groups: Remarkable Reusability and Substrate Selectivity in Rh Catalysis. *Angew. Chem. Int. Ed.* **2008**, *47*, 5627-5630.

(30) Lazar, P.; Mach, R.; Otyepka, M. Spectroscopic Fingerprints of Graphitic, Pyrrolic, Pyridinic, and Chemisorbed Nitrogen in N-Doped Graphene. *J. Phys. Chem. C* **2019**, *123*, 10695-10702.

(31) Artyushkova, K.; Kiefer, B.; Halevi, B.; Knop-Gericke, A.; Schlogl, R.; Atanassov, P. Density Functional Theory Calculations of XPS Binding Energy Shift for Nitrogen-Containing Graphene-Like Structures. *Chem. Commun.* **2013**, *49*, 2539-2541.

(32) Zhang, N.; Qiu, H.; Liu, Y.; Wang, W.; Li, Y.; Wang, X.; Gao, J. Fabrication of Gold Nanoparticle/Graphene Oxide Nanocomposites and their Excellent Catalytic Performance. *J. Mater. Chem.* **2011**, *21*, 11080-11083.

(33) Zhang, X.; Shi, H.; Xu, B.-Q. Comparative Study Of Au/Zro_2 Catalysts in CO Oxidation And 1,3-Butadiene Hydrogenation. *Catal. Today* **2007**, *122*, 330-337.

(34) Sugie, A.; Somete, T.; Kanie, K.; Muramatsu, A.; Mori, A. Triethylsilane As A Mild And Efficient Reducing Agent For The Preparation Of Alkanethiol-Capped Gold Nanoparticles. *Chem. Commun.* **2008**, 3882-3884.

(35) Inagaki, Y.; Yonemura, H.; Sakai, N.; Makihara, Y.; Kawae, T.; Yamada, S. Magnetism of gold Nanorods Probed Using Electron Spin Resonance. *Appl. Phys. Lett.* **2016**, *109*, 072404.

(36) Casaletto, M. P.; Longo, A.; Martorana, A.; Prestianni, A.; Venezia, A. M. XPS study of Supported Gold Catalysts: the Role of Au^0 and $Au^{+\delta}$ Species As Active Sites. *Surf. Interface Anal.* **2006**, *38*, 215-218.
